# WAKE-mediated modulation of cVA perception via a hierarchical neuro-endocrine axis in *Drosophila* male-male courtship behaviour

**DOI:** 10.1038/s41467-022-30165-2

**Published:** 2022-05-06

**Authors:** Shiu-Ling Chen, Bo-Ting Liu, Wang-Pao Lee, Sin-Bo Liao, Yao-Bang Deng, Chia-Lin Wu, Shuk-Man Ho, Bing-Xian Shen, Guan-Hock Khoo, Wei-Chiang Shiu, Chih-Hsuan Chang, Hui-Wen Shih, Jung-Kun Wen, Tsuo-Hung Lan, Chih-Chien Lin, Yu-Chen Tsai, Huey-Fen Tzeng, Tsai-Feng Fu

**Affiliations:** 1 Department of Applied Chemistry, National Chi Nan University, Nantou, Taiwan; 2 Department of Biochemistry and Graduate Institute of Biomedical Sciences, College of Medicine, Chang Gung University, Taoyuan, Taiwan; 3 Institute of Biotechnology, National Tsing Hua University, Hsinchu, Taiwan; 4 Department of Neurology, Chang Gung Memorial Hospital, Linkou, Taiwan; 5 Brain Research Center, National Tsing Hua University, Hsinchu, Taiwan; 6 Department of Life Science and Life Science Center, Tunghai University, Taichung, Taiwan; 7 Institute of Systems Neuroscience, National Tsing Hua University, Hsinchu, Taiwan; 8 National Institute of Infectious Diseases and Vaccinology, National Health Research Institutes, Zhunan, Taiwan; 9 Institute of Biological Chemistry, Academia Sinica, Taipei, Taiwan; 10 Department of Psychiatry, School of Medicine, National Yang Ming Chiao Tung University, Taipei, Taiwan; 11 Tsaotun Psychiatric Center, Ministry of Health and Welfare, Nantou, Taiwan; 12 Department of Psychiatry, Taichung Veterans General Hospital, Taichung, Taiwan; 13 Center for Neuropsychiatric Research, National Health Research Institutes, Miaoli, Taiwan

**Keywords:** Olfactory receptors, Pheromone

## Abstract

The nervous and endocrine systems coordinate with each other to closely influence physiological and behavioural responses in animals. Here we show that WAKE (encoded by *wide awake*, also known as *wake*) modulates membrane levels of GABA_A_ receptor Resistance to Dieldrin (Rdl), in insulin-producing cells of adult male *Drosophila melanogaster*. This results in changes to secretion of insulin-like peptides which is associated with changes in juvenile hormone biosynthesis in the corpus allatum, which in turn leads to a decrease in 20-hydroxyecdysone levels. A reduction in ecdysone signalling changes neural architecture and lowers the perception of the male-specific sex pheromone 11-cis-vaccenyl acetate by odorant receptor 67d olfactory neurons. These finding explain why WAKE*-*deficient in *Drosophila* elicits significant male-male courtship behaviour.

## Abstract (web summary)

The authors show that the *Drosophila* master regulator WAKE modulates the secretion of insulin-like peptides, triggering a decrease in 20-hydroxyecdysone levels. This lowers the perception of a male-specific sex pheromone and explains why WAKE-deficient *Drosophila* flies show male-male courtship behaviour.

## Introduction

Olfaction is one of the primary sensory modality for chemical senses. The brain can detect and decode many external olfactory chemical cues for eliciting suitable behavioural responses. Therefore, many insects rely on various environmental and individually-derived olfactory chemical signals for mating decisions^1–3^. Variability in olfactory perception, associated with many factors such as genetics, age, sex, and nutritional status affects courtship behaviour^1–4^. Similar to all heterotrophic organisms, insects acquire nutrients that are essential for normal growth, development, and physiological maintenance from their food. However, evidence has shown that the nutrients could play a key role in modulating olfactory sensitivity including the adjustment of feeding behaviour according to metabolic demand^5–7^.

The nutrient signalling through the insulin/insulin-like growth factor 1 (IGF-1) pathways also serves to regulate physiological decisions associated with reproduction, growth, and ageing^8,9^. Growing evidence shows that the nutrient dependent insulin signalling pathway is crucial for female reproductive maturity and is associated with improved fertility in males^10^. Although insulin plays a major role in directly enhancing reproductive efficiency^11,12^, numerous other endocrine hormones affected by insulin signalling also play crucial roles in regulating reproduction. For example, the ecdysteroid hormone 20-hydroxyecdysone (20E, also known as ecdysone), coordinates with the sesquiterpenoid juvenile hormone (JH) and gonadotropic hormones to exert antagonistic effects on the regulation of metamorphosis and fertility^13,14^. JH and ecdysone signalling have also been demonstrated to regulate the courtship behaviour of several insects^15^, including *Drosophila*^16,17^, and influence male-male courtship behaviour in the case of ecdysone regulation^16,18,19^.

The *Drosophila melanogaster* gene, *wide awake* (Gene ID: 42676), an ortholog of human *ankfn1* and mouse *nmf9*, is known to be involved in the regulation of sleep by pigment-dispersing factor (PDF) expressing neurons^20^. GABAergic sleep-promoting neurons suppress the firing of both large and small ventral lateral neurons (l-LNvs and s-LNvs) expressing PDF through the GABA_A_ receptor resistant to dieldrin (Rdl) to control sleep onset^21,22^. Evidence indicates that WAKE interacts with Rdl to upregulate its levels and promote its localisation to the plasma membrane in l-LNvs, resulting in increased GABA sensitivity and decreased excitability, thereby promoting sleep at dusk^20,23^. In addition to circadian imbalances, mice with *nmf9* mutations exhibit vestibular function deficits and reduced fear conditioning^24^. Apart from pleiotropic physiological functions, there is an indication that WAKE-related neuronal functions are highly conserved across different species. As previous reported that WAKE contributes to up-regulating Rdl levels and promotes the localisation of RDL to the plasma membrane, to effectively maintain the neuron's sensitivity to GABA^20,25^. This study highlights the role of WAKE, as the hierarchical responsive master, through the insulin/insulin-like growth factor signalling (IIS) by modulating Rdl in insulin producing cells (IPCs) in the *Drosophila* brain, which produces three insulin-like peptides (Ilps)–Ilp2, 3 and 5. Further stabilisation of other endocrine hormones, JH and ecdysone, inhibits male-male courtship behaviour. The severe hormone imbalance caused by WAKE deficiency eventually decreases ecdysone signalling in Or67d olfactory sensory neurons (OSNs), in turn affecting their neural architecture and responsiveness to 11-cis-vaccenyl acetate (cVA). Physiological and behavioural responses often depend on endocrine hormone regulation of complex networks that affect neuronal biological processing^26–29^. This cascade may modulate *Drosophila* male-male courtship behaviour in the *wake* mutant. Here we proposed an interesting model whereby WAKE in IPCs, via a long course involving multiple molecular interactions, wherein imbalances in intricate neuro-endocrine networks affect specific nerve cells, which ultimately result male-male courtship behavioural responses.

## Results

### WAKE in the adult *Drosophila* nervous system modulates male-male courtship behaviour

**Fig. 1:**
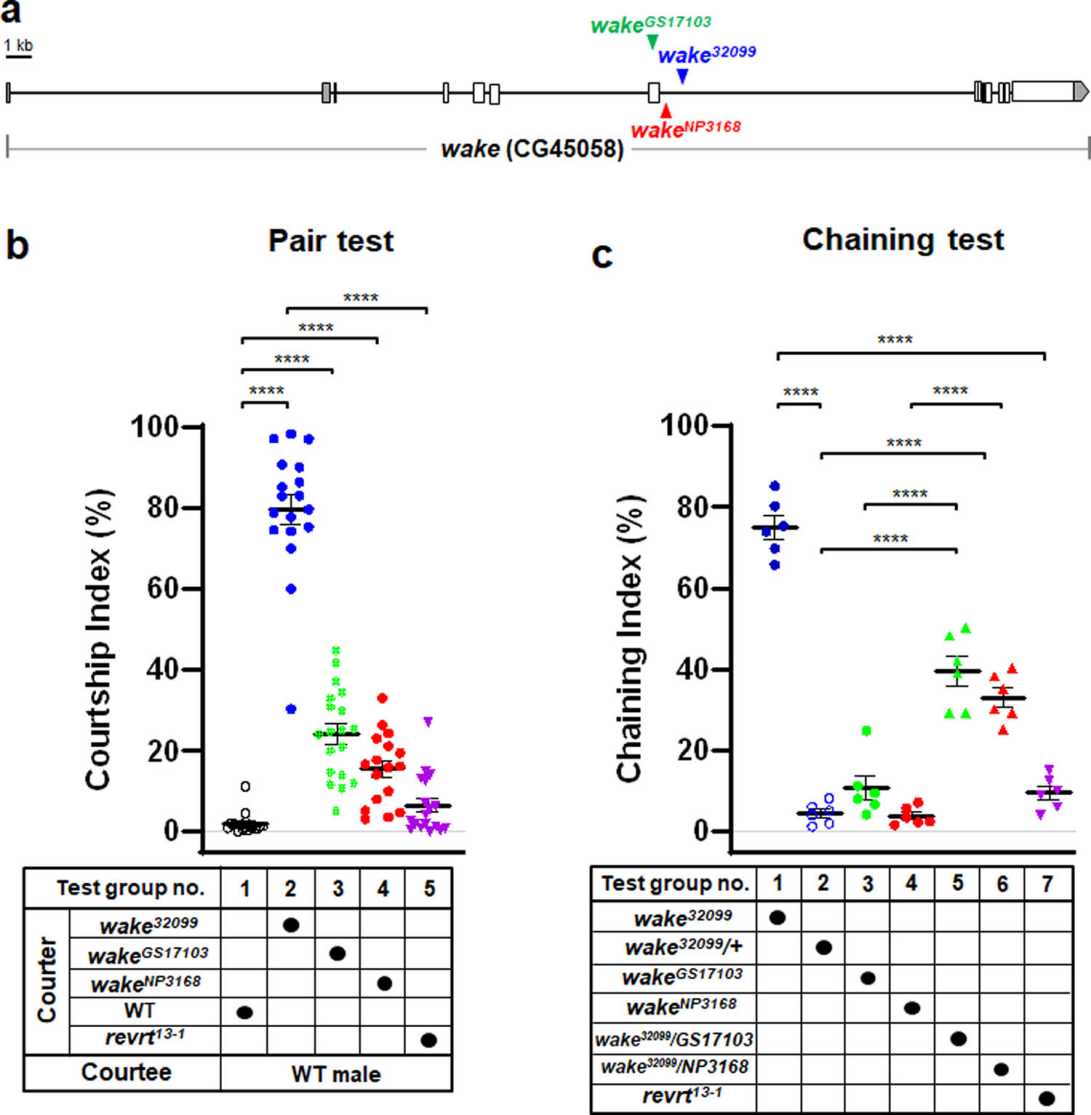
*wake* influences male-male courtship behaviour. The courtship and chaining indices were defined as the percentages of the 10-min observation period in which the corresponding male-male courtship behaviour was observed. **a** Locations of the insertions in the different *wake* mutants (*wake*^*32099*^, *wake*^*GS17103*^, and *wake*^*NP3168*^) are indicated by differently coloured arrowheads. **b** The *wake* insertion lines have higher male–male courtship behaviour indices than the wild-type (WT) or the *revrt*^*13-1*^ precise *wake*^*32099*^ transgene excision control lines. *n* = 18, 18, 19, 17 and 18 (from left to right) for each test, ^****^p < 0.0001, two-tailed Mann–Whitney U-test. **c**  Males homozygous for *wake*^*32099*^ also exhibited a higher level of male-male chaining behaviour than both *wake*^*32099*^ heterozygous (*wake*^*32099*^*/+*) and *revrt*^*13-1*^ males. In addition, males homozygous for *wake*^*GS17103*^ or *wake*^*NP3168*^ exhibited a very low level of chaining behaviour, which was significantly enhanced in *wake*^*32099*^*/wake*^*GS17103*^ and *wake*^*32099*^*/wake*^*NP3168*^ transheterozygotes. *n* = 6 for each. *p* < 0.0001, one–way ANOVA. ^****^p < 0.0001, *post hoc* Tukey’s multiple comparisons test. Scatterplots show error bars (± SEM) for all data points. Source data are provided as a Source Data file.

Increased male-male courtship chaining behaviour was observed in culture vials containing the *UAS-Mob2.eCFP* (BDSC #32099) stock from the Bloomington collection. Inverse PCR analysis was used to identify an insertion mutant in the *wake* (see Supplementary Fig. 1) in this line, which was designated as *wake*^*32099*^. To verify that *wake* mutations are associated with male-male courtship behaviour in *Drosophila*, we analysed two other *wake* lines (i.e., *wake*^*NP3168*^ and *wake*^*GS17103*^) with insertion sites near the *wake*^*32099*^ insertion site (Fig. 1a). We observed male-male courtship behaviour in these lines but not in wild-type flies with an identical genetic background as quantified by a Courtship Index (CI) (Fig. 1b; test group no. 1–4; Supplementary Movie 1). Additionally, while *wake*^*32099*^ flies exhibited significant male-male chaining behaviour when compared with heterozygous controls, as quantified by a Chaining Index (Fig. 1c; test group no. 1 vs. 2; Supplementary Movie 2), this behaviour was not observed in *wake*^*GS17103*^ and *wake*^*NP3168*^ males (Fig. 1c; test group no. 3 and 4). However, when these two mutant lines were crossed with *wake*^*32099*^ to generate transheterozygous flies, the chaining behaviour was observed (Fig. 1c; test group no. 5 vs. 2 or no. 5 vs. 3; test group no. 6 vs. 2 or no. 6 vs. 4; Supplementary Movie 3).

In the *P*-element inserted *wake*^*32099*^, the excision line (namely revertant #13-1; *revrt*^*13-1*^) was identified as a precise revertant line using nucleic acid sequencing (see Supplementary Fig. 2) and further used as a genetic control for the *wake*^*32099*^ mutant. Subsequent analysis of this revertant line exhibited that male-male courtship behaviour was significantly reduced (pair test, Fig. 1b, test group no. 2 vs. 5; chaining test, Fig. 1c, test group no. 1 vs. 7).

**Fig. 2:**
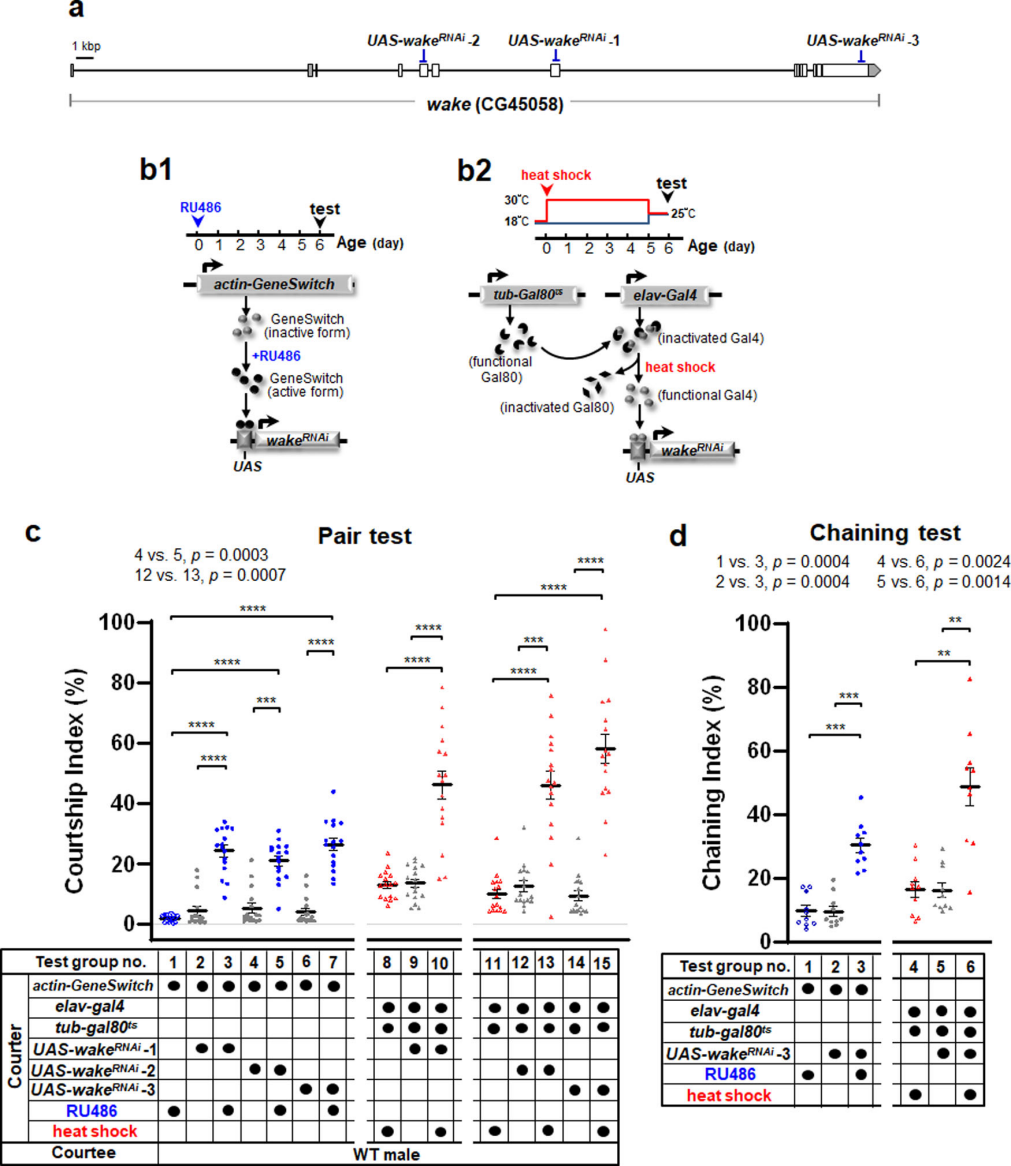
Downregulation of WAKE in early adulthood prompts male-male courtship behaviour. **a** The *wake* transcript target locations of *wake* dsRNA (*wake*^*RNAi*^) were elicited using the corresponding *UAS-wake* dsRNA stocks (*UAS-wake*^*RNAi*^−1, −2, and −3). **b1** Schematic representation of ubiquitous adult-onset activation of *wake*^*RNAi*^ expression using the *actin-GeneSwitch* and addition of the drug RU486 to food from eclosion to day 5. **b2** Schematic representation of the TARGET system. The pan-neuronal *elav-Gal4* driver was combined with ubiquitously expressed GAL80^ts^ to control the transcriptional activity of GAL4 and drive *wake*^*RNAi*^ expression in neurons upon heat induction after eclosion for 5 d. The strength of male-male courtship behaviour was tested at the 6-day time point in both regimens. **c** The scatterplots for the courtship indices include ± SEM for all data points, *n* = 16 for test group no. 1–7 and 11–15, *p* < 0.0001, Kruskal–Wallis test. ^***^p < 0.005 and ^****^p < 0.0001, *post hoc* Dunn’s multiple comparisons test. *n* = 16 for test group no. 8–10, *p* < 0.0001, one–way ANOVA. ^****^p < 0.0001, *post hoc* Tukey’s multiple comparisons test. **d** The chaining indices include ± SEM for all data points, *n* = 10 for each, *p* < 0.0001, Kruskal–Wallis test. ^**^p < 0.01, and ^***^p < 0.005, *post hoc* Dunn’s multiple comparisons test. Source data are provided as a Source Data file.

The *D. melanogaster* WT (2U) strain used for the pairs in this study is a *w*^*1118*^ (isoCJ1) Canton-S derivative^30^. We aimed to determine whether *wake*-induced male-male courtship behaviour was associated with a preference for specific genetic backgrounds. Further studies indicated that *wake*^*32099*^ flies exhibited male-male courtship behaviour toward the different genetic backgrounds despite white/red eye colouring (see Supplementary Fig. 3). To further verify the association between WAKE and male-male courtship behaviour, we downregulated *wake* using double-stranded RNA interference (*wake*^*RNAi*^). Three *UAS-wake*^*RNAi*^ lines (*UAS-wake*^*RNAi*^ −1, −2 and −3), based on independent constructs, were obtained from the Vienna *Drosophila* Resource Centre (VDRC), and different small dsRNA fragments were expressed to target various regions of the *wake* transcript (Fig. 2a), making it relatively easy to clarify and rule out the off-target RNAi effects. Following adult eclosion, expression of *wake* dsRNA was induced in most cells by feeding flies RU486 (*actin-GeneSwitch>UAS-wake*^*RNAi*^) (Fig. 2b1), and differing levels of male-male courtship behaviour were observed in induced males versus corresponding controls (pair test, Fig. 2c, test group no. 1–7, test group no. 3 vs. 1, test group no. 3 vs. 2, test group no. 5 vs. 1, test group no. 5 vs. 4, test group no. 7 vs. 1, test group no. 7 vs. 6; chaining test, Fig. 2d, test group no. 3 vs. 1, test group no. 3 vs. 2).

WAKE expressed in PDF neurons related to regulating sleep behaviour^20^. In addition, the mouse ortholog *Nmf9* is broadly expressed in the inner ear, amygdala, and suprachiasmatic nuclei^24^. Therefore, WAKE may be closely linked to the function of specific nerves. To further examine the roles of WAKE in the nervous system, the TARGET system^31^ was used for pan-neuronal spatiotemporal knock-down WAKE by increasing the temperature after adult eclosion (*elav-Gal4;tub-Gal80*^*ts*^*>UAS-wake*^*RNAi*^) (Fig. 2b2). Both pair tests (Fig. 2c, test group no. 8–15; test group no. 10 vs. 8, test group no. 10 vs. 9, and test group no. 13 vs. 11, test group no. 13 vs. 12, test group no. 15 vs. 11, test group no. 15 vs. 14) and chaining tests (Fig. 2d; test group no. 6 vs. 4, test group no. 6 vs. 5) demonstrated that WAKE in the adult nervous system is involved in the inhibition of male-male courtship behaviour. qPCR characterization of individual *UAS-wake*^*RNAi*^ lines revealed an effective reduction in relative *wake* RNA levels to Gal4-driver alone (see Supplementary Fig. 4).

### WAKE in IPCs modulates male-male courtship behaviour in *Drosophila*

**Fig. 3:**
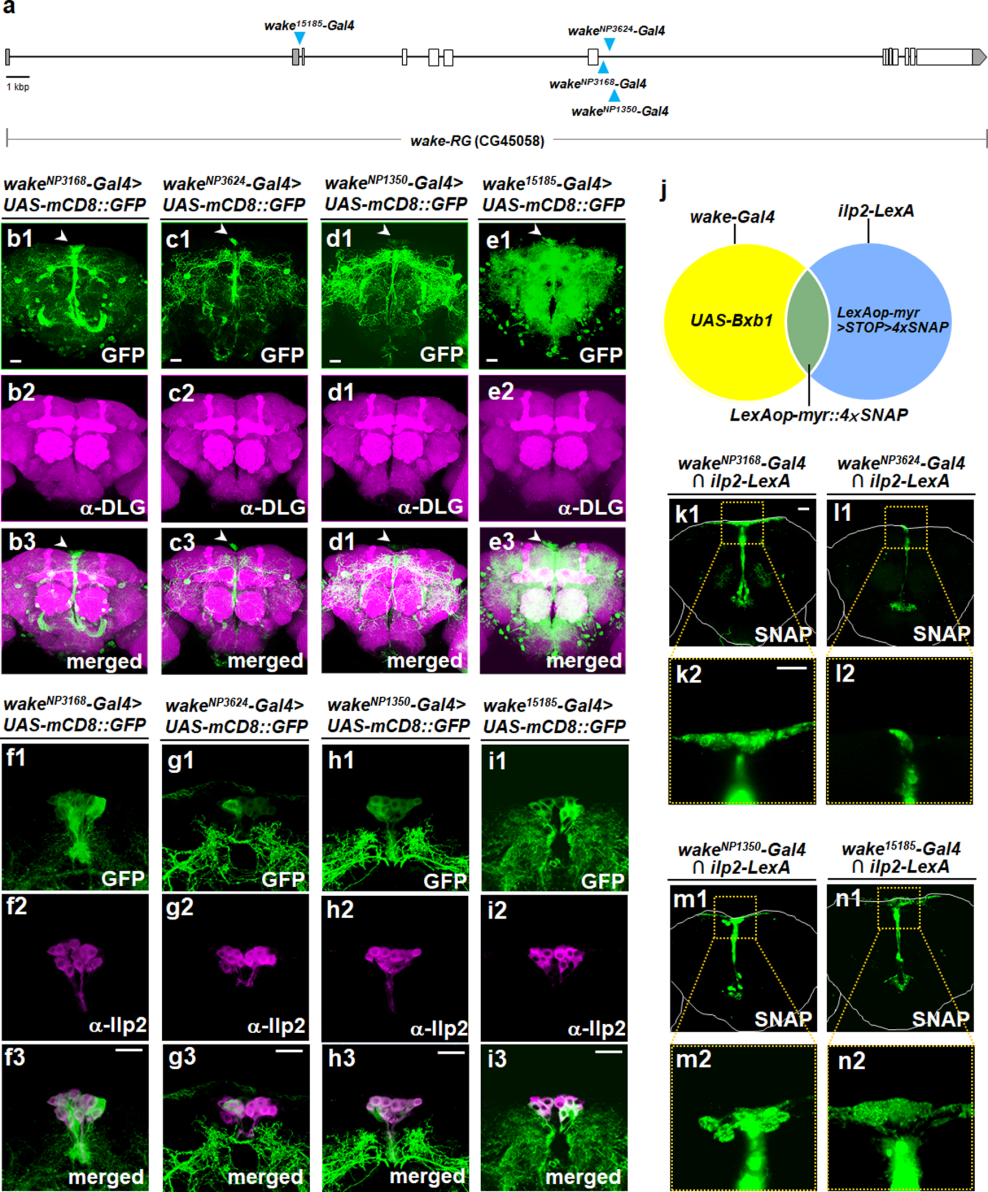
Identification of *wake-Gal4* drivers expressed in insulin-producing cells (IPCs) of the adult male brain. **a** *Gal4* transgene insertion locations on the *wake* gene in the different enhancer trap NP lines (*wake*^*NP3168*^, *wake*^*NP1350*^, and *wake*^*NP3624*^) and the *wake*^*15185*^*-Gal4* line generated using the CRISPR/Cas9 system are indicated using blue arrowheads. **b**–**e** Patterns of **b** *wake*^*NP3168*^, **c** *wake*^*NP3624*^, **d** *wake*^*NP1350*^, and **e** *wake*^*15185*^*-Gal4* expression, revealed using *UAS-mCD8::GFP*, in the adult male brain, represented in green in **b1**–**e1**, which show the cell bodies of median neurosecretory cells (MNCs; indicated by the arrowheads). The neuropil was immunostained using an anti-DLG antibody (magenta in **b2**–**e2**); merged in **b3**–**e3** (*n* = 6 for each). **f**–**i** The MNCs cell bodies exhibiting with four independent drivers (green in **f1**–**i1**) are IPCs, as evidenced by anti-insulin-like peptide 2 (anti-Ilp2) immunolabelling (magenta in **f2**–**i2**); merged in **f3**–**i3** (*n* = 6 for each). **j** Diagrammatic representation of the gene targeting strategy for restricting myr-4xSNAP expression to IPCs specifically using a Gal4 driven Bxb1 recombinase and a specific IPC-expression LexA intersectional method. The blue and yellow circles represent the patterns of *ilp2-LexA* and *wake-Gal4* expression, respectively. The resulting overlapping cell populations that express myr-4 × SNAPs are limited to the regions where Gal4 induced *UAS-Bxb1* expression, as the transcriptional stop cassette is removed (*LexAop-myr* > *STOP* > *4* × *SNAP* → *LexAop-myr::4* × *SNAP*) to allow LexA-induced expression, which represents the SNAP proteins that can be labelled specifically with molecular probes. **k**–**e** Representative images showing expression in IPCs with four independent drivers; green indicates the chemically labelled 4 × SNAPs expression pattern resulting from the intersectional *ilp2-LexA* and independent *wake-Gal4* drivers: **k** *wake*^*NP3168*^, **l** *wake*^*NP3624*^, **m** *wake*^*NP1350*^, and **n** *wake-Gal4*^*15185*^. Cell bodies of IPCs are indicated by dotted squares (**k1**–**n1**, respectively) (magnified in **k2**–**n2**; *n* = 8 for each). Scale bars, 20 µm.

**Fig. 4:**
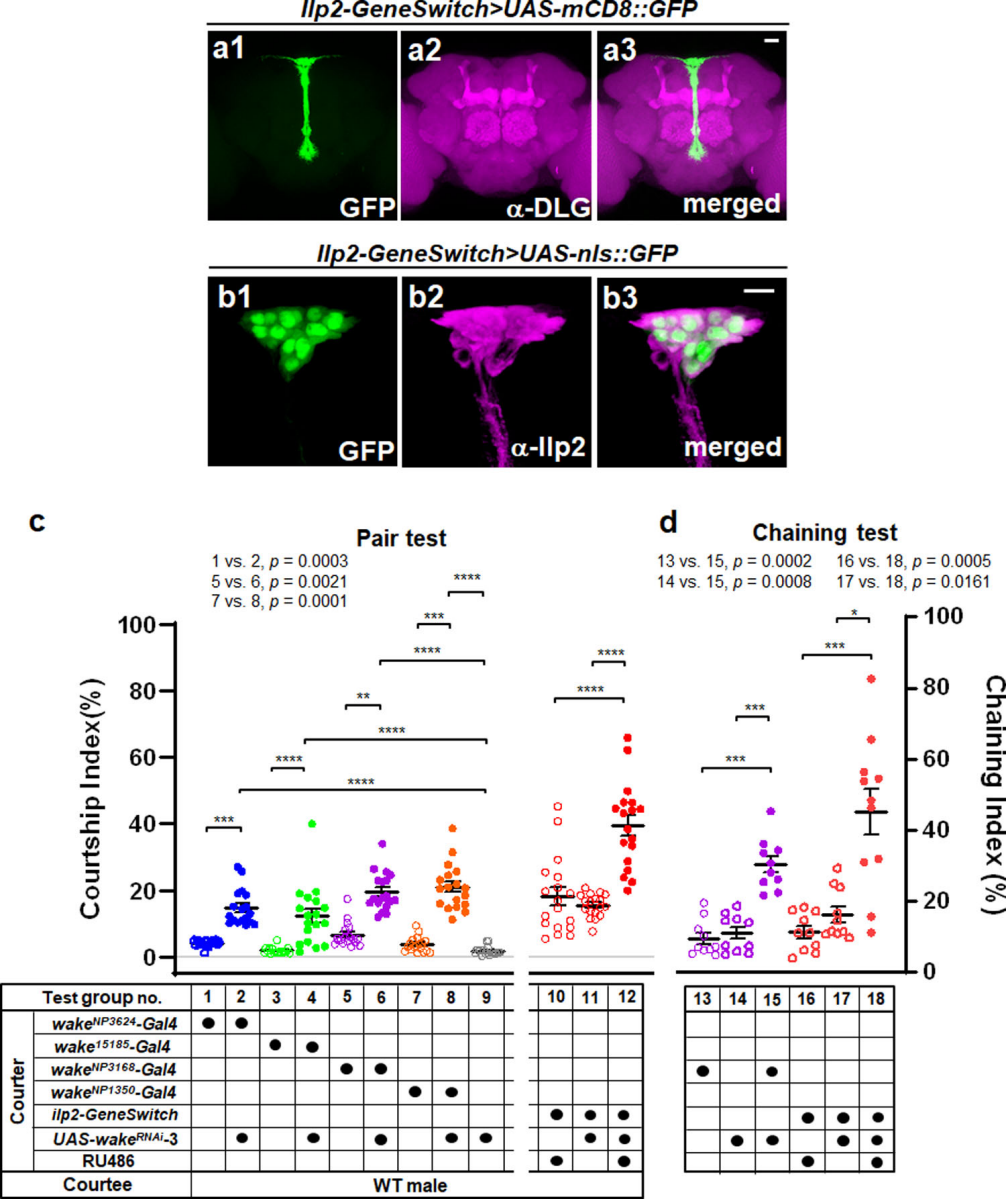
Downregulation of WAKE in insulin-producing cells in early adulthood modulates male-male courtship behaviour. **a** Representative images showing the pattern of expression for an RU486-inducible IPC-specific *ilp2-GeneSwitch* driver; expression patterns in the adult brain (10 days) revealed using *UAS-mCD8::GFP* are shown as green (*n* = 6 for each). The neuropil was immunostained using an anti-DLG antibody (magenta). **b** The cell bodies of IPCs can be observed due to *UAS-nls::GFP* for the nuclei (green) and were confirmed via anti-Ilp2 antibody staining (magenta) (*n* = 6 for each). Scale bars, 20 µm. **c**–**d** Comparison of genetically manipulated (*wake*^*NP3624*^*>UAS-wake*^*RNAi*^, *wake-Gal4*^*15185*^*>UAS-wake*^*RNAi*^, *wake*^*NP3168*^*>UAS-wake*^*RNAi*^, *wake*^*NP1350*^*>UAS-wake*^*RNAi*^) and RU486-treated (*ilp2-GeneSwitch>UAS-wake*^*RNAi*^) 5-day-old males with the corresponding controls. Scatter plots include ± SEM for all data points for **c** the Courtship Index (*n* = 18 for each) and **d** Chaining Index (*n* = 10, 10, 10, 10, 10 and 11; from left to right for test group no. 13–18), *p* < 0.0001, Kruskal–Wallis test. ^*^p < 0.05, ^**^p < 0.01, ^***^p < 0.005, and ^****^p < 0.0001, *post hoc* Dunn’s multiple comparisons test. Source data are provided as a Source Data file.

Apart from the *wake*^*NP3168*^ mutant described above, we also obtained two Gal4 enhancer trap lines (i.e., *wake*^*NP3624*^ and *wake*^*NP1350*^) adjacent to the insertion site of *wake*^*32099*^ and generated a *wake-Gal4* line (i.e., *wake*^*15185*^*-Gal4*) (Fig. 3a) by inserting the *Gal4* gene after the start codon of isoform-*wakeRG* using CRISPR/Cas9 technology (see Supplementary Fig. 5). A cluster of median neurosecretory cells (MNCs) (arrowheads in Fig. 3b–e) were observed in fluorescence images of intact central brains (Fig. 3b–e) and ventral nerve cords (VNC) in adult male flies (see Supplementary Fig. 6a–d) expressing mCD8::GFP driven by these drivers. Additionally, immunolabelling with an anti-Ilp2 antibody was used to verify the expression patterns of these Gal4 drivers and demonstrate that the MNCs included Ilps-expressing IPCs (Fig. 3f–i). Here we use another strategy involving a Gal4- and LexA-based intersectional genetic approach by a combination of the four *wake-*related Gal4 drivers and IPCs-specific *ilp2-LexA* driver (Fig. 3j). Then, *UAS-myr::SNAP* is expressed by GAL4 only in IPCs that are located in the regions targeted by *wake-*related Gal4 and *ilp2-LexA* (Fig. 3k–n). Further, we hypothesized that male-male courtship behaviour might be modulated by WAKE in IPCs. To verify this, we collected an RU486-inducible IPCs-specific driver (*ilp2-GeneSwitch*; Fig. 4a) and validated a cluster of 14 Ilp2-positive IPCs in the pars intercerebralis using anti-Ilp2 staining (Fig. 4b). For further study, these *wake*-related Gal4 and *ilp2-GeneSwitch* drivers were subsequently used to express *wake* dsRNA for WAKE downregulation and courtship behaviour assay. WAKE downregulation under the four *wake*-related Gal4 drivers, was associated with courtship behaviour in experimental flies when compared with control flies in both pair tests (Fig. 4c; test group no. 1–9; test group no. 2 vs. 1 or 9; test group no. 4 vs. 3 or 9; test group no. 6 vs. 5 or 9; test group no. 8 vs. 7 or 9) and chaining tests (Fig. 4d; test group no. 15 vs. 13 or 14). Notably, when WAKE downregulation was restricted to the IPCs of adults after eclosion, male-male courtship behaviour also occurred in RU486-treated flies when compared with the corresponding controls (pair tests, Fig. 4c; test group no. 12 vs. 10; test group no. 12 vs. 11 and chaining tests, Fig. 4d; test group no. 18 vs. 16 or 17). Overexpressing isoform WAKE-RG in IPCs on the WAKE-deficiency background significantly was associated with reduced male-male courtship behaviour (see Supplementary Fig. 7; test group no. 2 vs. 1); qPCR characterization of the *UAS-wake-RG* line revealed an effective increase in relative levels of *wake* RNA (see Supplementary Fig. 4). A previous study demonstrated that WAKE in PDF-expressing neurons affects sleep behaviour in *Drosophila*^20^; therefore, to further verify whether WAKE in PDF-expressing neurons also regulates male-male courtship behaviour, we specifically downregulated WAKE in PDF neurons, although this was not associated with increased male-male courtship behaviour (see Supplementary Fig. 7; test group no. 4 vs. 3). These results indicated that WAKE in IPCs at the adult stage is indeed involved in the inhibition of male-male courtship behaviour.

Moreover, in a competition test with one male test subject and two wild-type targets of different sexes, all male test subjects including *wake*^*32099*^ males and males with WAKE downregulation in IPCs exhibited significantly courtship behaviour towards the female target than the male target, with no change in their preference for females (see Supplementary Fig. 8a).

Given that motor activity and courtship behaviour are closely related, we next examined motor activity associated with WAKE expression in flies. Motor activity was assessed using a climbing test and a spontaneous locomotor test, which revealed that such motor abilities persist in *wake*^*32099*^ males and males with WAKE downregulation in IPCs (see Supplementary Fig. 8b).

### WAKE in IPCs affects Rdl to control male-male courtship behaviour in *Drosophila*

**Fig. 5:**
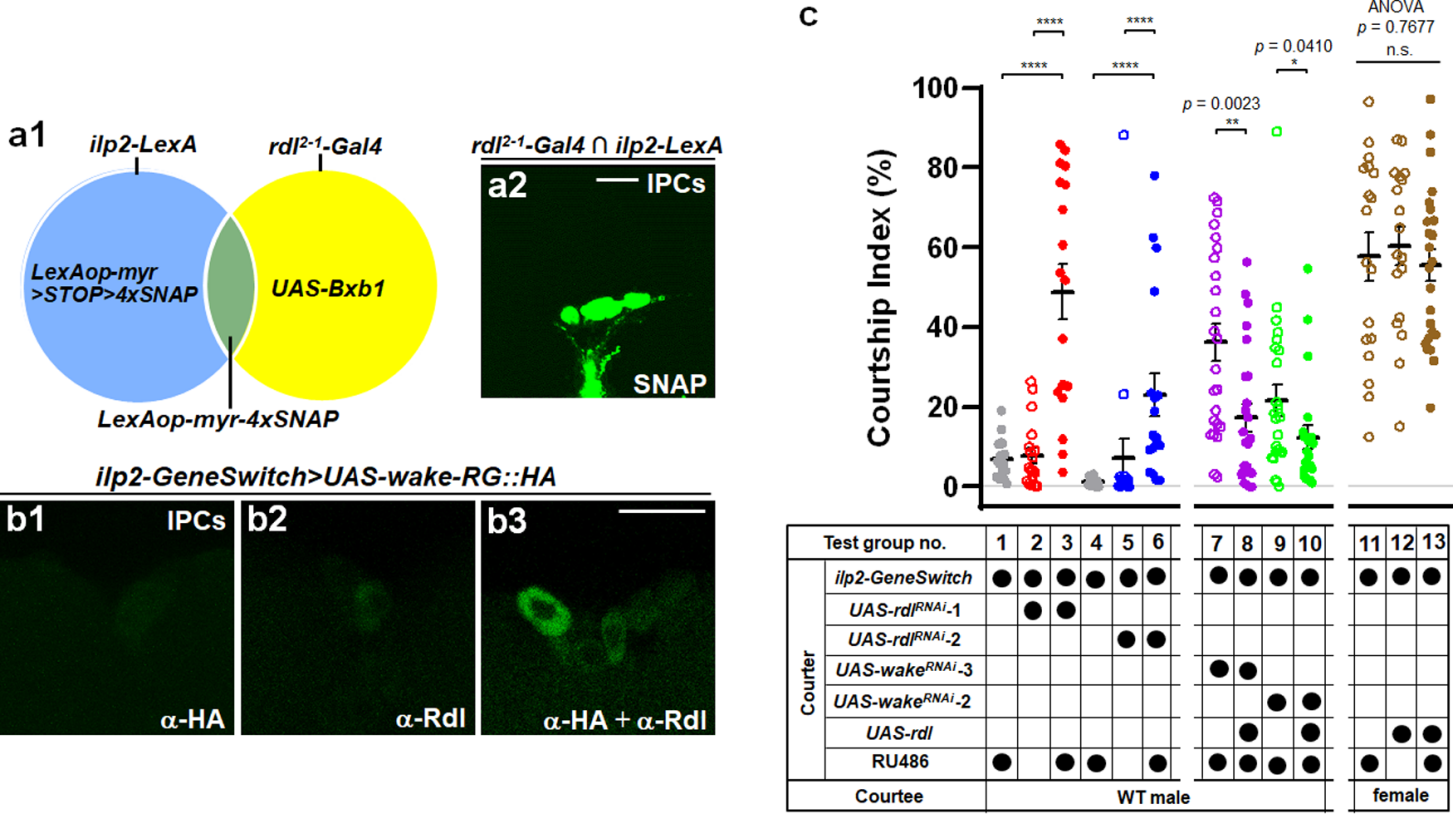
Rdl expressed in insulin-producing cells is involved in WAKE deficiency-dependent male-male courtship behaviour. **a1** The blue and yellow circles represent the patterns of *ilp2-LexA* and *rdl*^*2-1*^*-Gal4* expression, respectively. The overlapping cells that express 4 × SNAPs are limited to the regions where *rdl*^*2-1*^*-Gal4* drives *UAS-Bxb1* expression, as the transcriptional stop cassette is removed to allow LexA-induced expression, which represents the SNAP proteins that can be labelled specifically with molecular probes. **a2** Green indicates a pattern of expression for chemical labelling with 4 × SNAPs, zoomed in on the cell bodies of IPCs, resulting from the intersection of *ilp2-LexA* and the *rdl*^*2-1*^*-Gal4* driver (*n* = 6 for each). **b** Self-interaction of WAKE-RG::HA and Rdl was visualized in IPCs using an in situ proximity ligation assay (PLA). Comparison of representative images showing staining with anti-HA (**b1**) or anti-Rdl (**b2**) antibody as negative controls, respectively (*n* = 6 for each). Strong PLA signals for anti-HA and anti-Rdl (green in **b3**). Scale bars, 20 µm. **c** There was a significant difference between the Courtship index of untreated controls and *ilp2-GeneSwitch* tester males exhibiting *rdl* dsRNA expression due to treatment with RU486. *n* = 18 for test group no. 1–6, *p* < 0.0001, Kruskal–Wallis test. ^****^p < 0.0001, *post hoc* Dunn’s multiple comparisons test. Downregulation of WAKE in IPCs and simultaneous overexpression of Rdl significantly suppressed male–male courtship behavioural activity. *n* = 24, 24, 25, 22 (from left to right) for test group no. 7–10. ^*^p < 0.05, and ^**^p < 0.01, two-tailed Mann–Whitney U-test. The expression of Rdl alone in IPCs did not affect on male-female courtship behaviour. *n* = 18, 18, 25 (from left to right) for test group no. 11–13. *p* > 0.05 (n.s.), one-way ANOVA using F-test, F(2, 58) = 0.2656, *p* = 0.7677. Scatterplots include ± SEM for all data points. Source data are provided as a Source Data file.

As WAKE in PDF neurons affects sleep-related behaviour by regulating Rdl, a GABA_A_-receptor^20^, we next investigated whether WAKE-deficiency in IPCs also affects Rdl. A previous study reported that Rdl is not substantially expressed in IPCs^32^. Here, we performed immunolabelling with an anti-Ilp2 antibody to re-examine whether the *rdl*^*2-1*^*-Gal4* expression pattern includes IPCs. Preliminary results showed that in the extensive expression pattern of *rdl*^*2-1*^*-Gal4* (see Supplementary Fig. 9a), MNCs with nuclear labelling signals (*rdl*^*2-1*^*-Gal4>UAS-nls::GFP*) did not overlap perfectly with Ilp2-positive cells; however, several cells with a relatively weak GFP signal overlapped with Ilp2-positive cells (see Supplementary Fig. 9b). Similarly, using an intersectional recombination strategy, our findings demonstrated that *rdl*^*2-1*^*-Gal4* expression overlapped with *ilp2-LexA* expression in IPCs (Fig. 5a). Further, we performed an *in* *situ* proximity ligation assay (PLA)^33^, which provided a sensitive approach to validating WAKE-Rdl interactions in IPCs. We expressed the prey of WAKE-RG tagged with HA in IPCs (*ilp2-GeneSwitch>UAS-wake-RG*_*HA*_) using available HA and Rdl antibodies and succeeded in detecting PLA signals (Fig. 5b). The specificity of the anti-Rdl antibody was evaluated using immuno-labelling based on an ectopic expression study (see Supplementary Fig. 10). This evidence suggests that specifically expressed HA-tagged WAKE physically interacts with endogenous Rdl in IPCs. Whether WAKE is also involved in the stabilisation and localisation of Rdl requires confirmation in further studies. This implies that there is a significant drop in total intensity of Rdl::GFP fluorescence in IPCs with simultaneous down-regulation of WAKE (see Supplementary Fig. 11; quantification results in Supplementary Fig. 11c; test group no. 1 vs. 2). Similarly, we also found that WAKE deficiency significantly affects Rdl::GFP trafficking to the cell surface of IPCs when compared with the corresponding control (see Supplementary Fig. 11c; test group no. 3–6; test group no. 3 vs. 4).

Next, two *UAS-rdl*^*RNAi*^ lines (*UAS-rdl*^*RNAi*^−1 and −2) obtained from the VDRC were used for spatiotemporal control of *rdl* dsRNA expression in IPCs (*ilp2-GeneSwitch>UAS-rdl*^*RNAi*^), which was achieved via 5 d of RU486 treatment after eclosion. Male-male courtship behaviour was clearly observed in the RU486-treated flies when compared with corresponding controls (Fig. 5c; test group no. 1–6; test group no. 3 vs. 1; test group no. 3 vs. 2; test group no. 6 vs. 4; test group no. 6 vs. 5). Furthermore, the strength of male-male courtship behaviour was significantly suppressed upon synchronous overexpression of Rdl in IPCs (Fig. 5c; test group no. 7–10; test group no. 8 vs. 7; test group no. 10 vs. 9). However, the CIs of male-female courtship behaviour did not increase when Rdl was overexpressed in IPCs (Fig. 5c; test group no. 11–13; test group no. 13 vs. 11; test group no. 13 vs. 12). These results indicate that WAKE in IPCs specifically inhibits male-male courtship behaviour, which may be related to the modulation of Rdl.

### WAKE in IPCs modulates Ilp homoeostasis

**Fig. 6:**
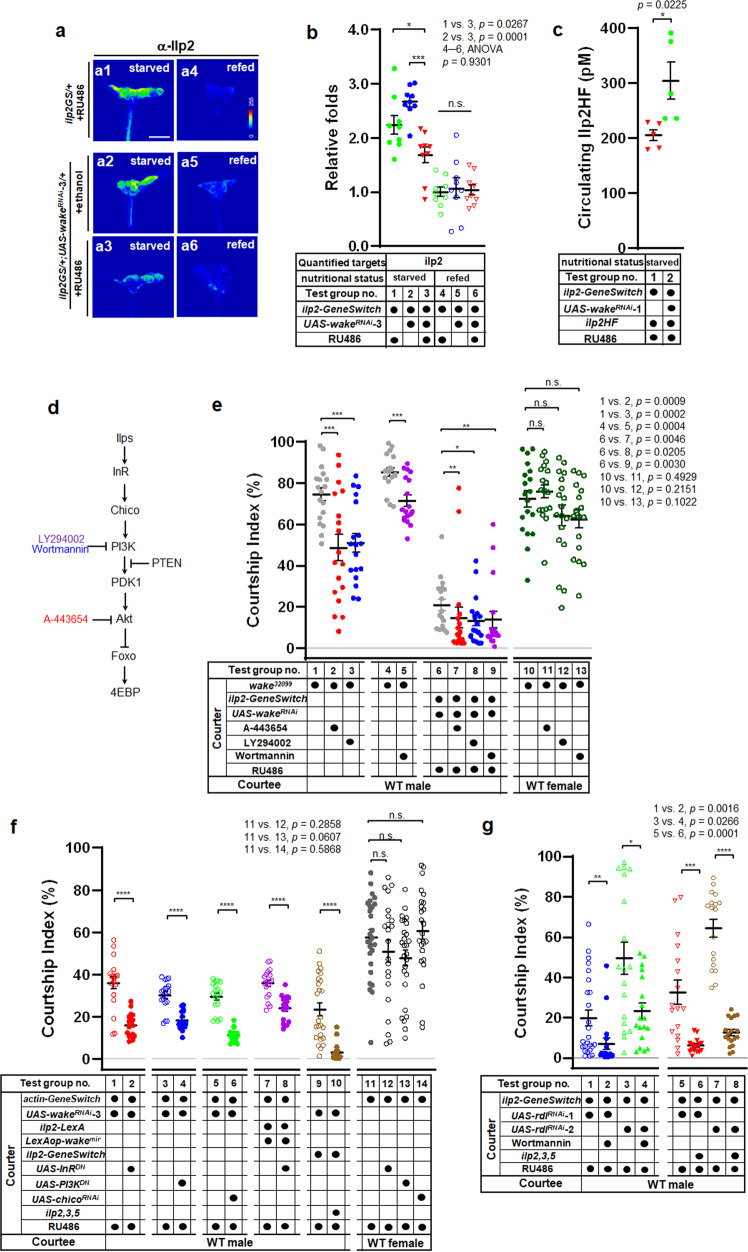
WAKE modulates male-male courtship behaviour through IIS. **a** Relative Ilp2 immunofluorescence signals in IPCs with WAKE downregulation after 24 h of starvation (**a1**–**a3**) (*n* = 9 for each), and after feeding for 30 min (**a4**-**6**) (*n* = 9 for each); quantification in **b**; *n* = 9 for test group no. 1–3, *p* = 0.0002, one-way ANOVA using F-test, *post hoc* Tukey’s multiple comparisons test, ^*^p < 0.05, and ^***^p < 0.005, *n* = 9 for test group no. 4–6, *p* > 0.05 (n.s.), one-way ANOVA using F-test, F(2, 24) = 0.07273, *p* = 0.9301. **c** The concentration of Ilp2HF in haemolymph with WAKE-deficient in IPCs. *n* = 5 for each, ^*^p < 0.05, two-tailed unpaired *t*-test. **d** Insulin signalling pathways indicating the targets of inhibitors used in further experiments. **e** In *wake*^*32099*^ flies or those with WAKE-deficient in IPCs, simultaneous treatment with different inhibitors. *n* = 18, 18, 18, 17, 18 (from left to right) for test group no. 1–5, *n* = 19, 18, 18, 17 for test group no. 10–13. *p* > 0.05 (n.s.), ^***^p < 0.005, two-tailed unpaired *t-*test. *n* = 18, 18, 19, 18 for test group no. 6–9. ^*^p < 0.05 and ^**^p < 0.01, two-tailed Mann–Whitney U-test. **f** In WAKE-deficient background simultaneous expression of InR^DN^, PI3K^DN^, or *chico* dsRNA, respectively. *n* = 18 for test group no. 1, 2, 7, 8, ^****^p < 0.0001, two-tailed unpaired *t-*test. *n* = 18 for test group no. 3–6, *n* = 25, 17 for test group no. 9, 10, ^****^p < 0.0001, two-tailed Mann–Whitney U-test. *n* = 27, 22, 27, 24 for test group no. 11–14, *p* > 0.05 (n.s.), two-tailed unpaired *t-*test. **g** Downregulation of Rdl in IPCs, simultaneous treatment with Wortmannin or those with heterozygous *ilp2, 3, 5* mutants. *n* = 25, 18, 18, 18, 18, and 18 for test group no. 1–4, 7 and 8. ^*^p < 0.05, ^**^p < 0.01 and ^****^p < 0.0001, two-tailed Mann–Whitney U-test. *n* = 18, 16 for test group 5 and 6, ^****^p < 0.0001, two-tailed unpaired *t-*test. Scatterplots include ± SEM. Source data are provided as a Source Data file.

Thus far, our results have suggested that WAKE inhibits male-male courtship behaviour by upregulating Rdl in IPCs. We also aimed to determine whether Ilp release is affected when Rdl levels in IPCs are insufficient to receive inhibitory GABA signals. In the fasting state, insulin secretion can be synchronised and maintained at low levels from individuals. Thus, we aimed to reduce individual variation and effectively quantify the insulin-related reactions during fasting in the subsequent assays. Immunolabelling of brain tissue using an anti-Ilp2 antibody under starvation conditions revealed that the Ilp2 signal in IPCs after WAKE downregulation was significantly lower than that in the corresponding controls (Fig. 6a1–3; quantification results in Fig. 6b; test group no. 1–3; test group no. 3 vs. 1 and test group no. 3 vs. 2). However, if the flies were then allowed to feed for 30 min, secretion of Ilp2 remained normal (Fig. 6a4–6; quantification results in Fig. 6b; test group no. 4–6).

In *Drosophila*, ligand-activated insulin receptor (InR) phosphorylates a *chico* encoded insulin receptor substrate (IRS), to induce the phosphorylation cascade of phosphoinositide-3-kinase (PI3K), phosphoinositide-dependent-kinase-1, and AKT (protein kinase B) (Fig. 6d). We further analysed fluorescence of tGPH, a PH-GFP fusion protein used as an indicator of PI3K activity, to evaluate insulin signalling^34^. Membrane localisation of tGPH was observed in the fat body, and the *wake*^*32099*^ background was associated with significant recruitment of the tGPH reporter to the cell membrane, suggesting that PI3K signalling is activated by IIS even in starved flies (see Supplementary Fig. 12a1, 2; quantification results in Supplementary Fig. 12b; test group no. 1 vs. 2). Similarly, these flies were then allowed to feed for 30 min, and a significant increase in tGPH fluorescence at the membrane was still observed in wild-type flies (see Supplementary Fig. 12a3, 4; quantification results in Supplementary Fig. 12b; test group no. 3 and 4). Moreover, enzyme-linked immunosorbent assay (ELISAs) for endogenous dual HA- and flag-tagged Ilp2 (Ilp2HF^35^) also showed that circulating Ilp2HF levels in the haemolymph were significantly higher in IPCs with WAKE downregulation than in the corresponding controls after 24 h of fasting (Fig. 6c; test group no. 1 vs. 2). In *Drosophila*, increased IIS results in a decrease in lifespan and reduced resistance to starvation stress^36,37^. Here, our analysis of *wake*^*32099*^ flies and those with WAKE downregulation in IPCs or Rdl expression also indicated a substantial reduction in lifespan and starvation tolerance in males (see Supplementary Fig. 13 and Supplementary Table 1).

### WAKE modulates IIS to evoke male-male courtship behaviour

We then verified whether enhanced IIS after WAKE deficiency in IPCs is what prompts male-male courtship behaviour in *Drosophila*. First, significant male-male courtship behaviour was observed in males overexpressing Ilp2 in IPCs for 5 d after eclosion (see Supplementary Fig. 12c; test group no. 3 vs. 1; test group no. 3 vs. 2). Next, to examine whether augmented secretion of Ilp2 in WAKE*-*deficient males leads to male-male courtship behaviour, we inhibited IIS in the canonical PI3K-PKB/AKT pathway using suitable enzyme inhibitors (Fig. 6d); 20 μM A-443654 to inhibit Akt activity^38^ and 300 nM LY294002 or 5 mM wortmannin to inhibit PI3K activity^39^. In *wake*^*32099*^ males (Fig. 6e; test group no. 1–5; test group no. 2 vs. 1; test group no. 3 vs. 1; and test group no. 5 vs. 4) and males with WAKE downregulation in IPCs (Fig. 6e; test group no. 6–9; test group no. 7 vs. 6; test group no. 8 vs. 6; and test group no. 9 vs. 6), the strength of male-male courtship behaviour after 5 days of treatment following eclosion was significantly decreased when compared with that in untreated controls. Although *wake*^*32099*^ flies were also treated with different IIS inhibitors, this did not substantially influence the CIs of male-female courtship behaviour (Fig. 6e; test group no. 10–13; test group no. 11 vs. 10; test group no. 12 vs. 10; test group no. 13 vs. 10), suggesting that drug treatment did not result in a general inhibition of courtship behavioural activity. Thus, pharmacological inhibition of IIS effectively reduced male-male courtship behaviour even in flies with WAKE deficiency.

Moreover, *actin-GeneSwitch* was used to induce WAKE downregulation at the adult stage (*actin-GeneSwitch>UAS-wake*^*RNAi*^) while simultaneously inducing the overexpression of dominant-negative insulin receptor (InR^DN^), dominant-negative PI3K (PI3K^DN^), or *chico* dsRNA (*chico*^*RNAi*^) for IIS inhibition. Male-male courtship behaviour was also significantly reduced in flies subjected to these genetic regimens when compared with the corresponding controls (Fig. 6f; test group no. 1–6; test group no. 2 vs. 1; test group no. 4 vs. 3; and test group no. 6 vs. 5). Similarly, a reduction in male-male courtship behaviour was observed when overexpression of InR^DN^ was induced by *actin-GeneSwitch* at the adult stage when WAKE was downregulated in IPCs only (*ilp2-LexA>LexAop-wake*^*mir*^) (Fig. 6f; test group no. 8 vs. 7). In the *ilp2,3,5* heterozygous background, WAKE downregulation in IPCs at the adult stage (*ilp2-GeneSwitch>UAS-wake*^*RNAi*^) also significantly reduced male-male courtship behaviour when compared with that in the corresponding controls (Fig. 6f; test group no. 10 vs. 9). Dominant InR^DN^, PI3K^DN^, or *chico*^*RNAi*^ expression induced in adults by *actin-GeneSwitch* did not significantly alter the CIs of male-female courtship behavioural activity (Fig. 6f; test group no. 11–14; test group no. 12 vs. 11; test group no. 13 vs. 11; test group no. 14 vs. 11). These findings again indicate that decreases in male-male courtship behaviour due to genetic inhibition of IIS are not reflective of general courtship suppression. To further demonstrate that the male-male courtship behaviour observed after Rdl downregulation in IPCs (*ilp2-GeneSwitch>UAS-rdl*^*RNAi*^) is caused by the increased secretion of Ilps, flies were treated with wortmannin, which also significantly reduced male-male courtship behaviour (Fig. 6g; test group no. 1–4; test group no. 2 vs. 1; test group no. 4 vs. 3). In the heterozygous *ilp2,3,5* mutant background, Rdl downregulation in IPCs also significantly reduced male-male courtship behaviour when compared with the corresponding controls (Fig. 6g; test group no. 5–8; test group no. 6 vs. 5; test group no. 8 vs. 7).

### WAKE deficiency in IPCs promotes JH biosynthesis via IIS in the corpus allatum, resulting in male-male courtship behaviour in *Drosophila*

Based on the evidence described above, we assumed that WAKE in IPCs modulates the activity of Rdl to maintain Ilps homoeostasis; thus, our next focus was to explore how Ilps evoke male–male courtship behaviour. In *Drosophila*, IIS is triggered by eight different Ilps that have varying spatiotemporal expression patterns and functions, and its pleiotropic effects regulate growth, development, metabolism, ageing, and stress responses^40^. However, only two Ilp receptors (InR and Lgr3) have been identified^41,42^. The ovarian development of female insects is associated with IIS in a programmed response by which they initiate their reproductive function after nutrient acquisition^43^—Ilps modulate the corpus allatum (CA) to synthesize JH via IIS, which in turn initiates ovarian development^44^. If this mechanism also modulates JH expression in male flies at the adult stage, WAKE-deficient males may also exhibit increased JH production from the CA via enhanced insulin signals. Indeed, a previous study reported that JH is required for courtship behavioural activity in adult *Drosophila* males^17^, and mutation of *methoprene-tolerant* (*met*), a JH receptor gene, also results in defects in mating behaviour and reproduction^45^.

**Fig. 7:**
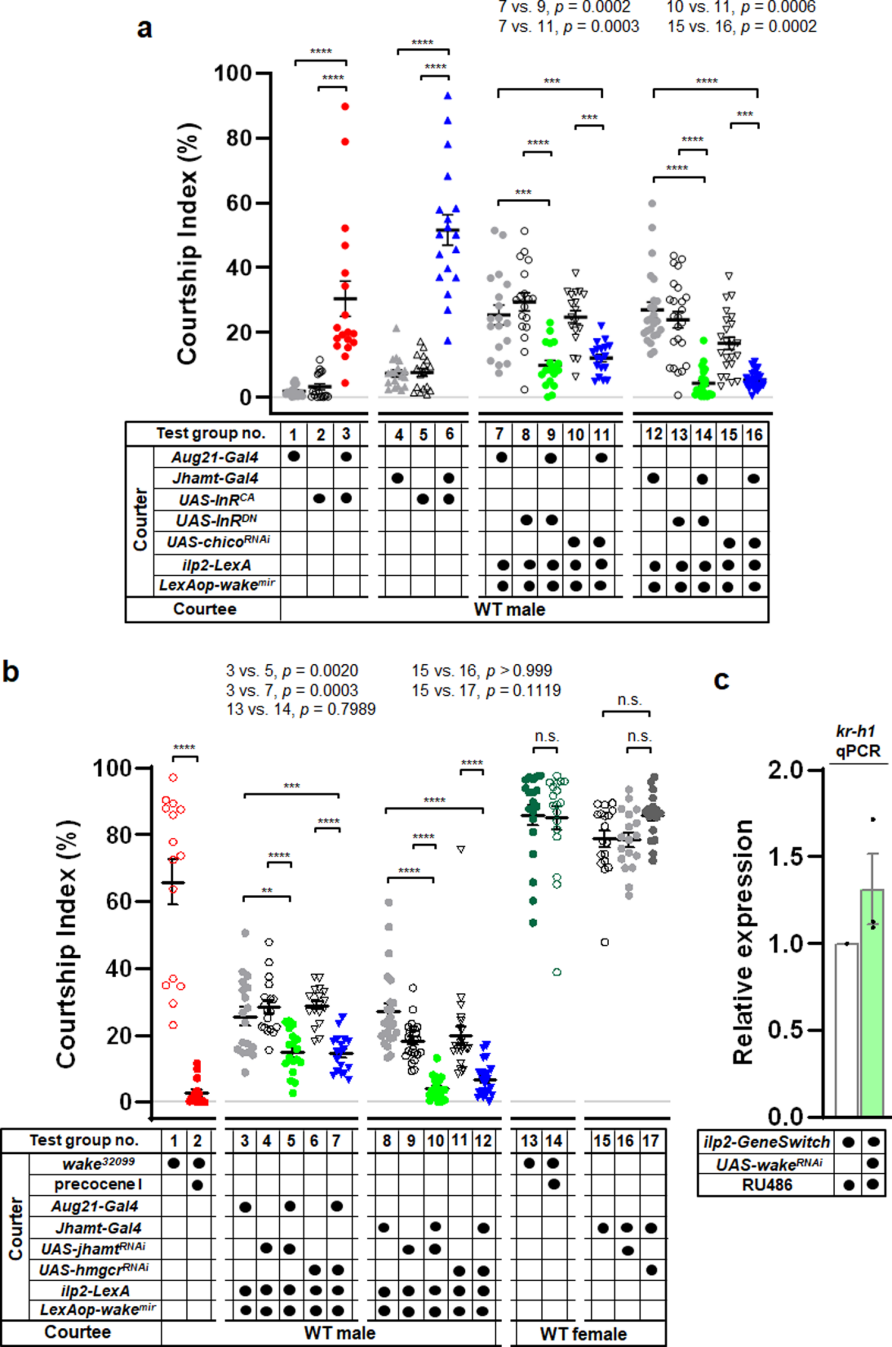
WAKE modulates male-male courtship behaviour via IIS-regulated JH biosynthesis in the CA. **a** Over-expression of InR^CA^ in the CA effectively evoked male-male courtship behaviour. WAKE downregulation in IPCs simultaneous expression of dominant negative-InR or *chico* dsRNA in the CA significantly suppressed male-male courtship behaviour. *n* = 18 for each test group no. 1–6, *n* = 24 for each test group no. 12–16. *p* < 0.0001, Kruskal–Wallis test. ^***^p < 0.005 and ^****^p < 0.0001, *post hoc* Dunn’s multiple comparisons test. *n* = 18 for each test group no. 7–11, *p* < 0.0001, one-way ANOVA using F-test, *post hoc* Tukey’s multiple comparisons test, ^***^p < 0.005 and ^****^p < 0.0001. **b** After eclosion, *wake*^*32099*^ treated with precocene I significantly reduced male-male courtship behaviour. *n* = 15 for test group no. 1 and 2. ^****^p < 0.0001, two-tailed Mann–Whitney U-test. WAKE-deficient in IPCs and simultaneous expression of *jhamt* or *hmgcr* dsRNA in the CA significantly suppressed male-male courtship behaviour. *n* = 18 for test group no. 3–7, *p* < 0.0001, one-way ANOVA using F-test, *post hoc* Tukey’s multiple comparisons test, ^**^p < 0.01, ^***^p < 0.005 and ^****^p < 0.0001. *n* = 24 for test group no. 8–12. *p* < 0.0001, Kruskal–Wallis test. ^****^p < 0.0001, *post hoc* Dunn’s multiple comparisons test. *wake*^*32099*^ males treated with precocene I or simultaneous expression *jhamt* or *hmgcr* dsRNA in the CA did not affect male-female courtship behaviour. *n* = 19, 18 for test group no. 13 and 14. *p* > 0.05 (n.s.), two-tailed Mann–Whitney U-test. *n* = 18, 18, 17 (from left to right) for test group no. 15–17. *p* = 0.0413, Kruskal–Wallis test, *p* > 0.05 (n.s.), *post hoc* Dunn’s multiple comparisons test. **c** Relative expression of the juvenile responsive-gene *kr-h1* via qPCR, *n* = 3 for each. Scatterplots include ± SEM. Source data are provided as a Source Data file.

The role of JH signalling in male-male courtship behaviour has rarely been discussed in *Drosophila*^46^. We observed increased activity of IIS in the CA through the expression of constitutively active insulin receptor (InR^CA^) by CA-expressing drivers (*Aug21-Gal4* or *Jhamt-Gal4*). Both drivers significantly prompted male-male courtship behaviour when compared with the corresponding controls (Fig. 7a; test group no. 1–6; test group no. 3 vs. 1; test group no. 3 vs. 2; and test group no. 6 vs. 4; test group no. 6 vs. 5). Conversely, WAKE downregulation in IPCs (*ilp2-LexA* > *LexAop-wake*^*mir*^) and simultaneous overexpression of InR^DN^ or *chico* dsRNA to block IIS in the CA led to significant inhibition of male-male courtship behaviour (Fig. 7a; test group no. 7–16; test group no. 9 vs. 7; test group no. 9 vs. 8; test group no. 11 vs. 7; test group no. 11 vs. 10; and test group no. 14 vs. 12; test group no. 14 vs. 13; test group no. 16 vs. 12; test group no. 16 vs. 15). These results demonstrate that WAKE deficiency in IPCs is likely to cause male-male courtship behaviour in *Drosophila* by facilitating IIS expression in the CA.

Because the CA promotes JH biosynthesis via IIS, we further aimed to determine whether enhanced JH signalling evokes male-male courtship behaviour. We first treated *wake*^*32099*^ flies with precocene I, an anti-juvenoid that inhibits JH synthesis^47^, for 5 days immediately after eclosion. The strength of male-male courtship behaviour was significantly suppressed in the treated flies when compared with that in the corresponding controls (Fig. 7b; test group no. 2 vs. 1). Alternatively, WAKE downregulation in IPCs (*ilp2-LexA* *>* *LexAop-wake*^*mir*^) and simultaneous overexpression of two dsRNAs of JH biosynthetic enzymes—3-hydroxy-3-methylglutaryl CoA reductase (*hmgcr*)^48^ or juvenile hormone acid *o*-methyltransferase (*jhamt*)^49^ in the CA were used to inhibit JH production, leading to a significant reduction in male–male courtship behaviour (Fig. 7b; test group no. 3–12; test group no. 5 vs. 3; test group no. 5 vs. 4; test group no. 7 vs. 3; test group no. 7 vs. 6; and test group no. 10 vs. 8; test group no. 10 vs. 9; test group no. 12 vs. 8; test group no. 12 vs. 11). In *wake*^*32099*^ males treated with precocene I, the expression of *jhamt*^*RNAi*^ or *hmgcr*^*RNAi*^ in the CA did not lead to significant changes in the CIs of male-female courtship behaviour (Fig. 7b; test group no. 14 vs. 13; test group no. 16 vs. 15; test group no. 17 vs. 15), again indicating that the observed in male-male courtship behaviour was not reflective of general courtship behaviour suppression. Interestingly, a direct comparison of relative JH action using qPCR was associated with slight increases in the expression of the early response *kr-h1* in flies with WAKE downregulation in IPCs (*ilp2-GeneSwitch>UAS-wake*^*RNAi*^) on the 5th day after induction (Fig. 7c). Thus, the male-male courtship behaviour in *Drosophila* prompted by WAKE deficiency in IPCs is likely due to the enhancement of JH biosynthesis by the CA via IIS.

### WAKE deficiency in IPCs modulates JH signalling to reduce ecdysone signalling

During insect growth and development, JH and ecdysone signalling exert antagonistic effects on many biological processes, including moulting and reproduction. Especially in the developmental period from the larval stage to adulthood, precise regulation of JH and ecdysone levels is necessary for normal metamorphosis^50^. However, although extremely low ecdysone levels are maintained during the adult stage^51^, the ecdysone receptor (EcR) is still widely expressed in various tissues, including the brain and other peripheral tissues^52^. Consistent with a previous report^53^, our immunolabelling experiments indicated that EcR type A (EcRA)-positive cells were also detected throughout the adult brain (see Supplementary Fig. 14a–c). Therefore, ecdysteroids may retain their physiological functions in adult *Drosophila*, and they are involved in several physiological responses, including those related to oogenesis (i.e., germline development)^54,55^, the circadian clock^55,56^, stress resistance and longevity^57^. Moreover, male-male courtship behaviour has been observed in *Drosophila* upon the inhibition of ecdysteroid biosynthesis or mutation of the EcR in male flies^16,18,19^. Additional research has demonstrated that courtship behaviour is regulated by EcR in *fruitless* (*fru*) *P1-*expressing neurons^16^.

**Fig. 8:**
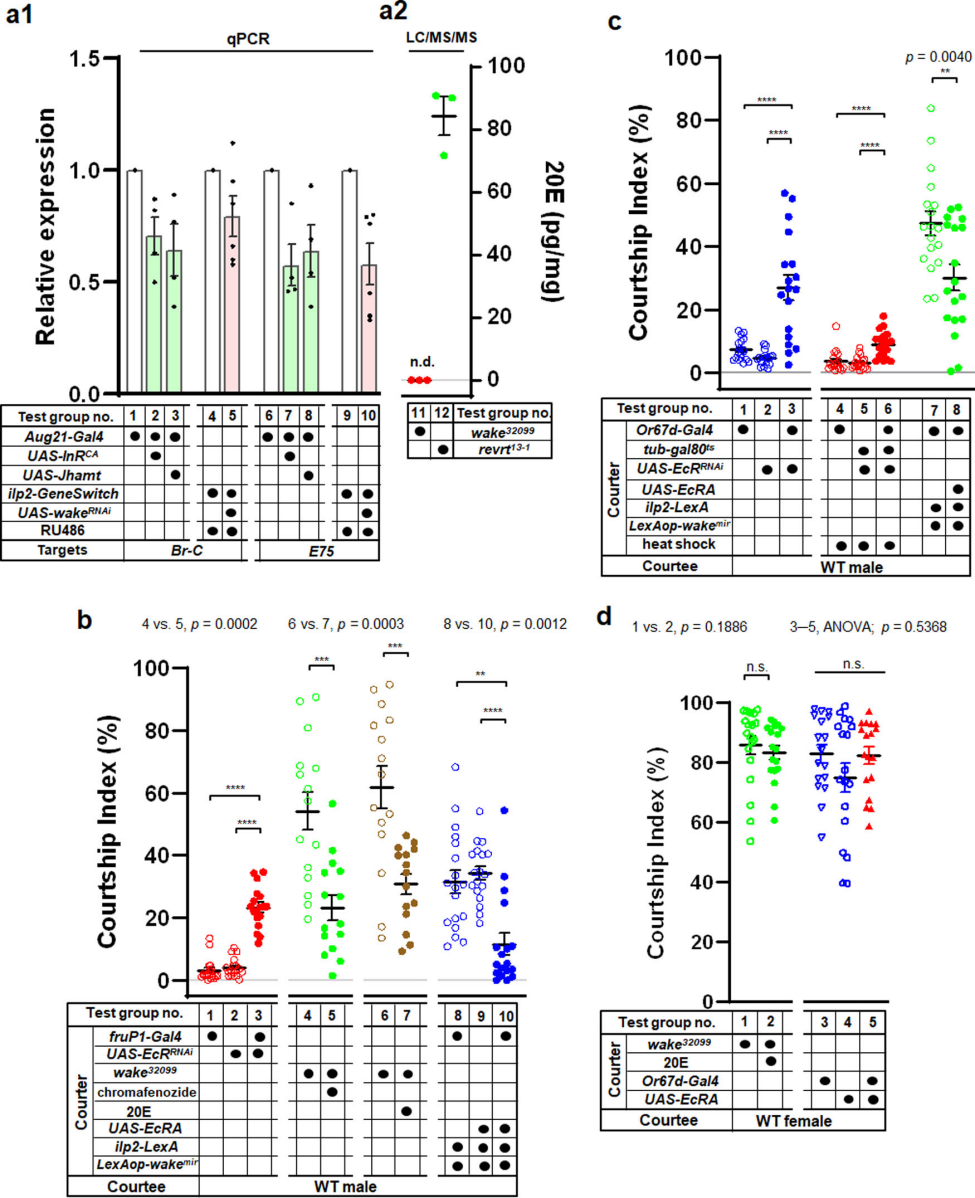
WAKE modulates male-male courtship behaviour by ecdysone signalling in Or67d neurons. **a1** Activation of IIS enhancement of JH biosynthesis in the CA significantly decreased the relative expression of the ecdysone responsive-genes *Br-C* and *E75*, *n* = 4 for each group. Moreover, relative expression of *Br-C* and *E75* also significantly decreased with WAKE deficiency in IPCs, *n* = 5 and 6 for each group. **a2** Levels of 20E in *wake*^*32099*^ flies were significantly lower than those in the revertant control (n.d. means not detected). **b** EcR knockdown in *fru-P1* neurons blocked ecdysone-dependent signalling, effectively prompting male-male courtship behaviour. Downregulation of WAKE in IPCs and simultaneous expression of EcRA in *fru-P1* neurons significantly suppressed male-male courtship behaviour. *n* = 18 for test group no. 1–3 and 8–10. *p* <  0.0001, Kruskal–Wallis test. ^**^p < 0.01 and ^****^p < 0.0001, *post hoc* Dunn’s multiple comparisons test. *n* = 15 for test group no. 4–7. ^***^p < 0.001, two-tailed unpaired *t*-test. **c** Directly or conditional knockdown of EcRs in Or67d neurons blocked ecdysone-dependent signalling, effectively prompting male-male courtship behaviour. *n* = 18 for test group no. 1–3. *p* < 0.0001, one-way ANOVA. ^****^p < 0.0001, *post hoc* Tukey’s multiple comparisons test. *n* = 18 for test group no. 4–6. *p* < 0.0001, Kruskal–Wallis test. ^****^p < 0.0001, *post hoc* Dunn’s multiple comparisons test. Downregulation of WAKE in IPCs and simultaneous expression of EcRA in Or67d neurons significantly suppressed male-male courtship behaviour. *n* = 18 for test group no. 7 and 8. ^**^P <  0.01, two-tailed unpaired *t*-test. **d** There were no significant effects on male–female courtship behaviour in *wake*^*32099*^ males treated with 20E or the males expressing EcRA in Or67d neurons. *n* = 19 and 18 for test group no. 1 and 2. *p* > 0.05 (n.s.), two-tailed Mann–Whitney U-test. *n* = 17, 18, 18 for test group no. 3–5. *p* > 0.05 (n.s.), Kruskal–Wallis test. Scatterplots include ± SEM. Source data are provided as a Source Data file.

The present findings indicate that activation of IIS in the CA may lead to male-male courtship behaviour in WAKE-deficient flies promoting JH signalling (Fig. 7). Since JH and ecdysone exhibit antagonistic actions in the regulation of many physiological processes, we then examined whether JH biosynthesis promoted by IIS in the CA results in male-male courtship behaviour due to the inhibition of ecdysone signalling. Relative quantification of the expression of the early response genes *Br-C* and *E75* by qPCR was used to verify the strength of ecdysone signalling^13^. Ecdysone signalling was significantly reduced under conditions of IIS activation in the CA (*Aug21-Gal4>UAS-InR*^*CA*^), increased JH synthesis (*Aug21-Gal4>UAS-Jhamt*^58^) (Fig. 8a1; test group no. 1–3 and 6–8), and WAKE downregulation in IPCs (*ilp2-LexA>LexAop-wake*^*mir*^) (Fig. 8a1; test group no. 4, 5 and 9, 10). Moreover, quantitative analysis using liquid chromatography with tandem mass spectrometry (LC-MS-MS) clearly showed that 20E levels were much lower than the detection limit in terms of per individual or per milligram of dry weight in *wake*^*32099*^ flies. In the controls (*revrt*^*13-1*^), however, 20E levels were approximately 22 ± 3pg/fly or 84 ± 6 pg/mg (Fig. 8a2; test group no. 11 vs. 12). These results demonstrate that WAKE deficiency induces the CA to increase JH levels via IIS, which may reduce 20E synthesis via JH signalling and thereby reduce ecdysone signalling.

### WAKE deficiency reduces ecdysone signalling in Or67d OSNs and affects their responsiveness to cVA

In adult male *Drosophila*, *fruP1-*expressing neurons participate in the inhibition of male-male courtship behaviour via ecdysone signalling^16^. This phenomenon was also clearly observed in the current study upon expression of *EcR* dsRNA in *fruP1-*expressing neurons (*fruP1-Gal4>UAS-EcR*^*RNAi*^), which was used to downregulate ecdysone signalling (Fig. 8b; test group no. 1–3; test group no. 3 vs. 1; test group no. 3 vs. 2). To verify that the reduction in ecdysone signalling causes male-male courtship behaviour after WAKE deficiency, we treated *wake*^*32099*^ males with 20E or chromafenozide^59^ (a non-steroidal ecdysteroid agonist) just after eclosion for 5 d, which significantly reduced the strength of male-male courtship behaviour (Fig. 8b; test group no. 5 vs. 4; test group no. 7 vs. 6). Alternatively, in the genetic background of WAKE downregulation in IPCs (*ilp2-LexA>LexAop-wake*^*mir*^), EcR-A was overexpressed in *fruP1-*expressing neurons to enhance ecdysone signalling, which also suppressed male-male courtship behaviour when compared with that in the corresponding controls (Fig. 8b; test group no. 8–10; test group no. 10 vs. 8; test group no. 10 vs. 9).

**Fig. 9:**
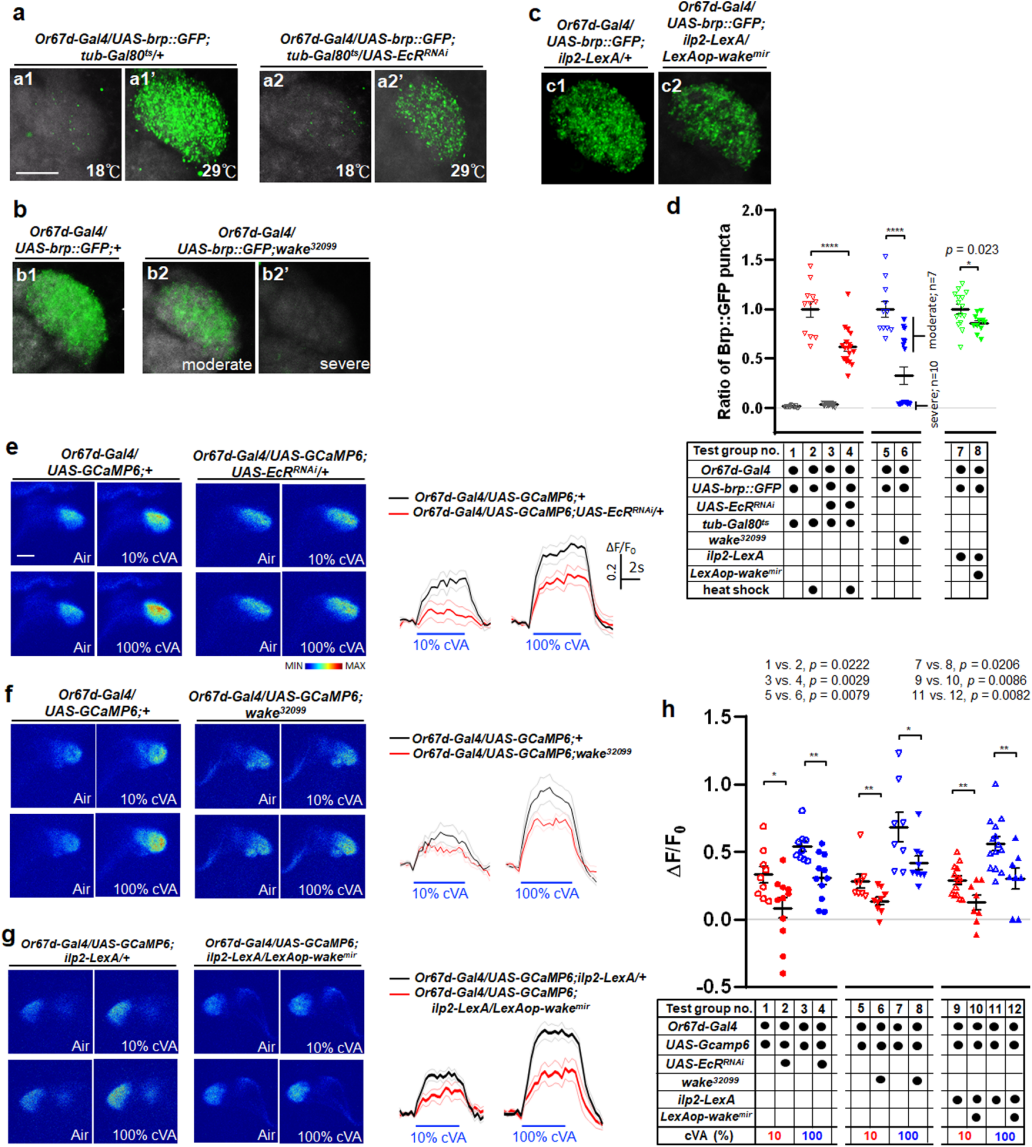
WAKE modulates male-male courtship behaviour by affecting the perception of cVA by ecdysone signalling in Or67d neurons. High-magnification confocal stack images of a single DA1 glomerulus with putative synapses labelled using brp::GFP in the adult male brain (*Or67d-Gal4>UAS-brp::GFP*). The neuropil was immunostained using an anti-DLG antibody (magenta). There were significant reductions in the number of brp::GFP puncta signals (green) compared to those in adult flies simultaneously specific expressing EcRi (**a**) and in *wake*^*32099*^ mutant flies (**b**) when compared with the corresponding controls, respectively. Moreover, brp::GFP puncta signals were also significantly decreased in flies with WAKE deficiency in IPCs (*ilp2-LexA>LexAop-wake*^*mir*^) (**c**) when compared with the corresponding control, *n* = 10, 11, 19, 18, 11, 10, 7, 15 and 11 (from **a**1 to **c**2); data quantified in **d**; *n* = 10, 11, 19 and 18 (from left to right) for test group no. 1–4. ^****^p < 0.0001, two-tailed unpaired *t-*test. *n* = 11 and 17 for test group no. 5 and 6. ^****^p <  0.0001, two-tailed Mann–Whitney U-test; showing the distribution of data points for moderate and severe levels within the bar chart of test group no. 6. *n* = 15 and 11 for test group no. 7 and 8. ^*^p < 0.05, two-tailed unpaired *t*-test. **e**–**g** Representative images focusing on the DA1 glomerulus showing the Ca^2+^ response signals for *Or67d-Gal4* using *UAS-GCaMP6* in the adult male DA1 before (pre) and after simultaneous stimulation with 10% or 100% cVA. **e** Summary of Ca^2+^ response data from controls (*Or67d/UAS-GCaMP6*) compared with those from *wake*^*32099*^ flies (**f**) with downregulated ecdysone signalling in Or67d neurons due to *EcR* dsRNA expression and those from flies with WAKE-deficient in IPCs (**g**). Responsiveness to cVA significantly increases when compared with that in flies with EcR knock-down, *wake*^*32099*^ mutants, and flies with WAKE-deficient IPCs; quantified in (**h**); *n* = 9, 11, 9, 11 and 14, 8, 14, 8 (from left to right) for test group no. 1–4 and 9–12. ^*^p < 0.05 and ^**^p < 0.01, two-tailed unpaired *t*-test. *n* = 8, 9, 8, and 9 for test group no. 5–8. ^*^p < 0.05 and ^**^p < 0.01, two-tailed Mann–Whitney U-test. Scatterplots include ± SEM. Source data are provided as a Source Data file. Scale bar, 20 μm.

Previous evidence has shown that *fruP1-Gal4* is expressed in subsets of OSNs that innervate different glomeruli, including the sexually dimorphic DA1, VA1lm, and VL2a glomeruli in the antennal lobes; moreover, these OSNs may be regulated by Fru to result in obvious differences in their volumes between sexes^60^. In the case of EcR-A deficiency in *fruP1-*expressing neurons, evident male-male courtship behaviour occurs in *Drosophila*, along with a reduction in the volume of the DA1 glomerulus^16^; it is assumed that Or67d OSNs, which are responsible for receiving the male-specific pheromone cVA, reduce neural transmission due to a decrease in neural fibres projecting into the DA1 glomerulus. We focused on the antennal DA1 glomerulus, in which clear EcR-immunolabelling signals were also observed (see Supplementary Fig. 14d–e). To investigate whether WAKE deficiency can ultimately result in male-male courtship behaviour due to reduced ecdysone signalling in Or67d OSNs, we first expressed *EcR* dsRNA in Or67d neurons (*Or67d-Gal4>UAS-EcR*^*RNAi*^) to downregulate ecdysone signalling, which induced male-male courtship behaviour in these flies (Fig. 8c; test group no. 1–3; test group no. 3 vs. 1; test group no. 3 vs. 2). Next, temporary downregulation of EcR in Or67d neurons (*Or67d-Gal4;tub-Gal80*^*ts*^*>UAS-EcR*^*RNAi*^) by increasing the temperature for 5 d after adult eclosion only slightly prompted male-male courtship behaviour in these flies (average CI ~9.71% ± 0.96; Fig. 8c; test group no. 4–6; test group no. 6 vs. 4; test group no. 6 vs. 5). This suggests that ecdysone signalling plays an important role in Or67d neurons even outside of the adult stage. Furthermore, in the genetic background of WAKE downregulation in IPCs (*ilp2-LexA>LexAop-wake*^*mir*^), EcR-A was concurrently overexpressed in Or67d OSNs to rescue ecdysone signalling, which resulted in a significant reduction in male-male courtship behaviour (Fig. 8c; test group no. 8 vs. 7). However, when *wake*^*32099*^ males were treated with 20E or overexpressed EcR in Or67d neurons, there were no significant changes in the average CIs for basal male-female courtship behaviour (Fig. 8d; test group no. 2 vs. 1; test group no. 5 vs. 3; test group no. 5 vs. 4), confirming that the reduction in male-male courtship behaviour was not due to general suppression of courtship behaviour. Moreover, when ecdysone signalling was inhibited in Or67d neurons (*Or67d-Gal4>UAS-EcR*^*RNAi*^) or WAKE was downregulated in IPCs (*ilp2-LexA>LexAop-wake*^*mir*^), we simultaneously expressed mCD8::GFP in Or67d to quantify the fluorescence intensity (see Supplementary Fig. 15a1–d1) and voxel size (for screenshots of three-dimensional renderings of Z-stack images, see Supplementary Fig. 15a2–d2, which shown relatively obvious thinning) of the DA1 glomerulus. The two quantification results were significantly lower than those in the corresponding controls (see Supplementary Fig. 15e), suggesting that there were specific changes in the axonal fibres of Or67d neurons within the DA1 glomerulus. Next, we examined the subcellular distribution of Bruchpilot (brp), which forms a scaffold at the presynaptic active zone (AZ) and is correlated with synaptic transmission. We utilized Bruchpilot-GFP (brp::GFP)^61^ to quantitatively measure the number of AZs in the DA1 glomerulus and elucidate the pattern of synaptic assembly and connectivity between Or67d neurons and downstream neurons. Strikingly, temporary inhibition of ecdysone signalling in Or67d neurons at the permissive temperatures from adult emergence for 5 d (*Or67d-Gal4; tub-Gal80*^*ts*^*>UAS-EcR*^*RNAi*^) (Fig. 9a1–2) drastically reduced the number of brp::GFP fluorescence puncta signals within the DA1 glomerulus when compared with that in the corresponding controls. The quantification results were also significantly lower than those in the corresponding controls (Fig. 9d; test group no. 1–4; test group no. 4 vs. 2). Moreover, in the context of different genetic backgrounds of *wake*^*32099*^ (Fig. 9b) or WAKE downregulation in IPCs (*ilp2-LexA>LexAop-wake*^*mir*^) (Fig. 9c), the number of brp::GFP fluorescence puncta signals was also obviously reduced within the DA1 glomerulus. The quantification results were again significantly lower than those in the corresponding controls (Fig. 9d; test group no. 5–8; test group no. 6 vs. 5, and test group no. 8 vs. 7). The most pronounced effects were observed in *wake*^*32099*^ flies, with most affected individuals exhibiting moderate to severe signal levels (Fig. 9b2 and 2’), although some exhibited almost no signals. In *Drosophila*, the volatile male-specific pheromone cVA acts through the receptor Or67d to regulate both male and female mating decisions. The influence of WAKE on male-male courtship behaviour be mediated by structural changes in Or67d neurons due to ecdysone-dependent regulation, thereby affecting cVA perception. Thus, the neural activity sensor GCamp6.0 was expressed in Or67d neurons to analyse ΔF/F_0_ values obtained from the DA1 perfumed with cVA. This resulted in direct inhibition of ecdysone signalling in Or67d neurons (*Or67d-Gal4>UAS-EcR*^*RNAi*^) (Fig. 9e) in both *wake*^*32099*^ flies (Fig. 9f) and those with WAKE downregulation in IPCs (*ilp2-LexA>LexAop-wake*^*mir*^) (Fig. 9g). Quantitative in vivo calcium imaging further revealed that Or67d neurons within the DA1 glomerulus exhibited significantly decreased responses to 10% or 100% cVA when compared with the corresponding controls (Fig. 9h; inhibition of ecdysone signalling in Or67d neurons analyzed in test group no. 1–4 of the test group; test group no. 2 vs. 1; test group no. 4 vs. 3; *wake*^*32099*^ background analyzed in no. 5–8 of the test group; group no. 6 vs. 5; group no. 8 vs. 7; and WAKE downregulation in IPCs analyzed in no. 9–12 of the test group; group no. 10 vs. 9; group no. 12 vs. 11).

**Fig. 10:**
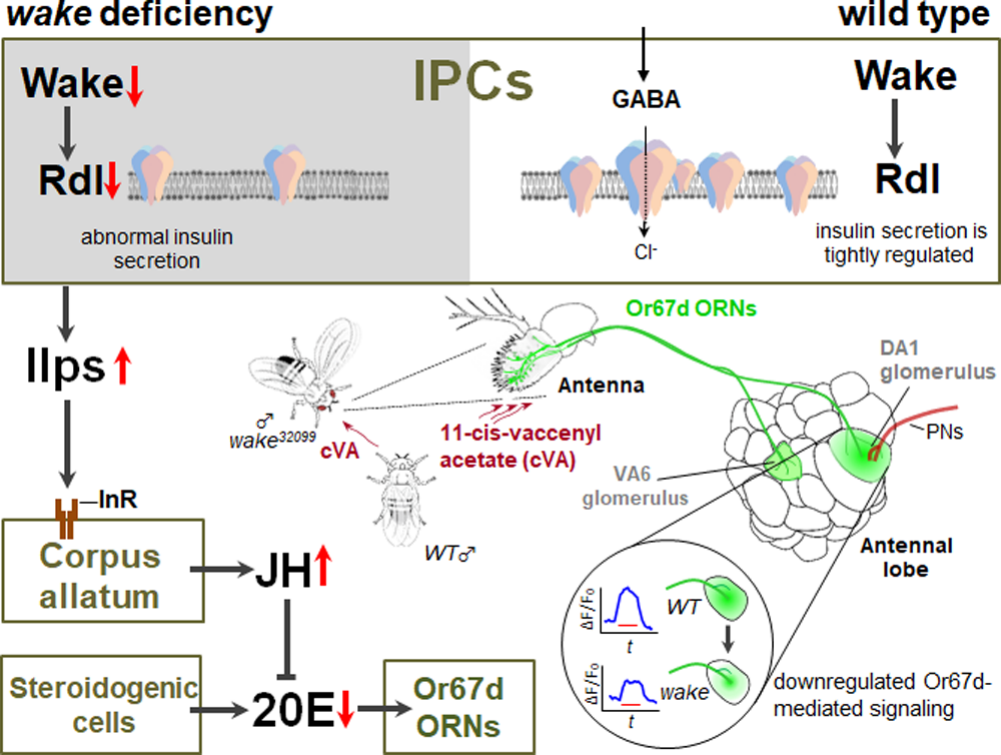
WAKE in IPCs modulates male-male courtship behaviour in *Drosophila* through endocrine network effects on Or67d neurons. WAKE in IPCs of the adult brain affects Rdl to modulate Ilp2 homoeostasis. WAKE in adult male IPCs modulates JH biosynthesis from the CA via IIS stabilization. It may reduce 20E synthesis via the continuous JH signalling, thereby reducing ecdysone signalling. Our results indicate that reduced ecdysone signalling results in modulating the neural architecture of Or67d neurons (nerve fibres indicated in green) and leads to reduced responsiveness to the male-specific cVA pheromone (blue lines), prompting significant male-male courtship behaviour.

In summary, in the male-male courtship behaviour phenotype observed in *Drosophila* with *wake* mutation, WAKE regulated Ilps secretion via the regulation of Rdl in IPCs, which in turn affects JH and ecdysone signalling. These findings suggest a hierarchical neuro-endocrine axis, with WAKE deficiency in IPCs inducing a series of changes in the endocrine network (Fig. 10). Finally, a decline in ecdysteroid signalling leads to morphological alterations and reduced sensitivity in cVA-detecting Or67d neurons, which may underlie the increased male-male courtship behaviour observed in WAKE-deficient flies.

## Discussion

Evidence shows that WAKE in the nervous system of larval and adult *Drosophila* participates in several essential biological processes^20,62^. Our results indicate that the functional maintenance of WAKE in IPCs is necessary for inhibiting the male-male courtship behaviour phenotype. Regardless of the WAKE-silencing efficiency of *wake* dsRNA, *wake*^*32099*^ flies displayed more male–male courtship behaviours (average CI ~79%; Fig. 1b) than flies with conditional knockdown of WAKE in IPCs (*ilp2-GeneSwitch>UAS-wake*^*RNAi*^) 5 d after emergence (average CI ~39%; Fig. 4c). Moreover, following adult eclosion, when expression of *wake* dsRNA was induced in most cells by *actin-GeneSwitch*, the strength of male-male courtship behaviour (average CI ~24%; Fig. 2c) was still lower than in *wake*^*32099*^ flies. Although *wake*^*32099*^ mutants exhibited significantly reduced male-male courtship behaviour through exogenous overexpression of WAKE in IPCs after eclosion, this was still insufficient for full rescue (see Supplementary Fig. 7). This suggests that WAKE may act in concert with other subpopulations of tissues to regulate male-male courtship behavioural responses. Furthermore, the male-male courtship behaviours observed in *wake* mutant flies may be attributable to a WAKE deficiency outside IPCs, and it is possible that WAKE deficiency during larval development can evoke male-male courtship behaviour in adults. Based on these assumptions, the effect of WAKE in PDF-expressing neurons can be ruled out; however, WAKE expression outside of IPCs maybe still involved in the regulation of male-male courtship behaviour (see Supplementary Fig. 7). This result also excludes the possibility that *wake* dsRNA interference can result in this courtship behaviour due to leaky expression, supporting the role of WAKE in IPCs in regulating male-male courtship behaviour. Nonetheless, further studies are required to confirm whether the biological processing of WAKE during development is associated with male-male courtship behaviour in adult *Drosophila*.

Researchers have also suggested that there is a close relationship between nutritional status and egg/offspring production. However, effects beyond those induced by nutritional status on insulin signalling are required for oocyte development, vitellogenesis, and egg production, which vary greatly with diets^12,63^. Insulin signalling has also been shown to affect various physiological processes that enable behavioural plasticity in both sexes of *Drosophila* and many other insect species. Many studies have demonstrated that insulin signalling mediates sexual attractiveness in *Drosophila* females and is involved in the regulation of cuticular hydrocarbons (CHCs) synthesis^64,65^. More interestingly, recent studies suggest that *Drosophila* males exhibit an innate preference for courting females raised on protein-rich diets, which may be related to altered insulin signalling in oenocytes, as this may drive female sexual attractiveness via changes in CHCs biosynthesis^66^. However, few studies have demonstrated a direct effect of insulin on male courtship behaviour or reproduction.

The biogenic amines serotonin and octopamine can regulate insulin secretion, which can indirectly affect male courtship behaviour^67^. The present findings suggest the involvement of a hierarchical neuro-endocrine axis in the inhibition of male-male courtship behaviour in *Drosophila*. In this pathway WAKE deficiency in neurosecretory IPCs of the brain modulates the GABA_A_ receptor, thereby promoting the secretion of Ilps. Subsequent enhancement of IIS and stimulation of JH biosynthesis in the CA lead to a decrease in ecdysone levels, which in turn induces morphological changes in Or67d neurons that reduce their ability to sense male-specific sex pheromone cVA. This may ultimately lead to increased male-male courtship behaviours. This effect is characterized by a long course involving multiple molecular interactions between neural and endocrine networks. Nonetheless, our findings highlight WAKE as the master regulator that stabilizes this overall network to maintain normal physiological responses. Further research is required to determine whether WAKE is modulated in IPCs by known nutritional cues or other mediators such as neurotransmitters and neuropeptides. Future studies must examine whether accurately modulating Rdl via WAKE can control changes in insulin signalling.

The genetic programming influenced by ecdysone signalling is involved in the control of nervous system remodelling during the developmental process, helping to promote various structural and functional changes considered favourable for the formation and stability of the nervous system^68–70^. Ecdysone is the major steroid hormone in insects, and it is known to be involved in the coordination of developmental transitions (e.g., larval moulting) and metamorphosis^71^. In *Drosophila*, specific pruning of axons and dendrites during metamorphosis is also controlled by ecdysone^72,73^. Although its function in adult insects is still largely unknown, there is considerable evidence to show that ecdysone can inhibit the proliferation of mushroom body neuroblasts and trigger their differentiation into interneurons in the brain of the adult house cricket (*Acheta domesticus*)^74^. Additionally, the canonical ecdysteroid transcriptional cascade has been detected in MBs of adult worker honeybees (*Apis mellifera* L.) after stimulation of ecdysone signalling^75^. Ecdysone may also participate in the remodelling of MB neurons at the adult stage, and studies have demonstrated that ecdysone signalling in the MBs of adult *Drosophila* participates in sleep maintenance^56^ and long-term courtship memory formation^53^, as well as in the inhibition of male-male courtship behaviour^16,18,19^. Future studies must examine the types of changes in molecular processing caused by inhibition of ecdysone signalling and the effects that these changes have on the neural architecture of Or67d neurons.

Courtship behaviour is dependent on several distinct subcomponents of specific circuits responsible for coordinating, performing, and processing information to elicit the appropriate behavioural responses. Using a variety of manipulations, we concluded that WAKE deficiency can lead to male–male courtship behaviour via an impairment in the ability of Or67d neurons to sense cVA. Similar manipulations may be required to identify and determine the influence of unexpected subpopulations within the courtship circuitry. Fru-expressing circuitry is broadly distributed in different sensory, central, and motor regions, indicating that information from such circuitry may be integrated to determine the courtship behaviour outcome.

The current results suggest that male-specific courtship behaviour is inhibited by a flow of information from Or67d sensory neurons that receive external cVA cues to DA1 projection neurons, which then pass information to DC1 interneurons in the lateral horn (LH). The LH then relays the cVA-induced signal to a descending neuron, DN1, which sends an axon to a thoracic ganglion to inhibit male courtship behaviour^76^. It is worth noting that all neurons within this circuit are Fru-positive neurons. Moreover, the male-specific P1 cluster is also composed of Fru-positive neurons, representing a potent activation of male courtship behaviour^77^. P1 neurons may arborize their neurites connect with P2b interneurons in the middle superior protocerebrum and extend a descending fibre to the thoracic ganglia to generate the pattern of courtship behaviours^77,78^. Herein, we demonstrated that WAKE, via regulation of Rdl in IPCs, influences the secretion of Ilps, which in turn increases circulating levels of JH. This sequence leads to decreases in ecdysone signalling, which induces morphological alterations in Or67d neurons that reduced their responsiveness to cVA. Throughout the current study, this phenomenon was also observed upon expression of *EcR* dsRNA in *fruP1-*expressing neurons (*fruP1-Gal4>UAS-EcR*^*RNAi*^) (Fig. 8b; test group no. 1–3). Although Or67d neurons represent a subset of *fruP1-*expressing neurons, the EcR expression pattern was observed in labelling experiments using three independent anti-EcR antibodies that are widely expressed in the adult brain (see Supplementary Fig. 14a–c). The widespread distribution of EcR expression suggests that ecdysone signalling plays an important role in the adult brain, although its function in adult insects remains largely elusive. However, it cannot be ruled out that ecdysone signalling affects other components of Fru circuitry to control additional aspects of courtship behaviour. In this study, *wake*^*32099*^ flies, in particular, exhibited higher levels male-male courtship behaviour than those with inhibition of ecdysone signalling in Or67d neurons. This indicates that *wake* may affect other ecdysone-sensitive subpopulations, leading to a synergistic effect that increases male-male courtship behaviour.

In addition, *Drosophila* females adjust their sexual behaviour to match their nutritional state, such that fed females are more receptive to male courtship^66,79^. Interestingly, cerebral insulin can also promote olfactory sensitivity after feeding^6,7^. More specifically, insulin signalling in Or67d OSNs is necessary for fed females to be attracted to a blend of food odours and cVA^79^, which will in turn help promote mating success and egg-laying^1,80^. Even if there is no evidence of a direct link between male courtship behaviour and the actions of insulin, several studies have shown that malnourished fruit flies preserve genital size to ensure reproductive success^81^. Moreover, short neuropeptide F receptor 1 (sNPFR1) in OSNs is regulated by insulin signalling and is linked with food-related fruity-smelling esters, the detection of which is involved in the regulation of feeding behaviour^7,82,83^. Interestingly, knock-down of neuropeptide F (NPF) leads to suppression of courtship behaviour^84^ and promotion of aggressive behaviour^85^. Furthermore, JH has been shown to regulate sexual behaviour^15,17,86^. JH coordinates the timing of female reproductive maturation in most insects^87^. However, whether the increases in Ilps and JH levels caused by WAKE deficiency also help to modulate distinct hormone-sensitive subpopulations involved in the regulation of male-male courtship behaviour remains to be determined.

## Methods

### Fly strains

Flies were raised on standard cornmeal food and housed at 25 °C in 70% relative humidity on a 12:12-h light:dark cycle. The wild type—2U strain used in this study is a *w*^*1118*^ (isoCJ1) Canton-S derivative^30^. Details regarding all fly strains used in this study and their sources are provided in Supplementary Table 2. For all brain images and behavioural analyses after transgenic expression, progeny obtained from Gal4- or LexA-based flies crossed to the flies with the indicated reporter or effector transgenes were used. For experiments involving the inducible GeneSwitch drivers, the progeny was first pre-treated with food containing 2 mM RU486 for at least 5 d for induction, after which subsequent imaging or behavioural analysis was conducted; control flies were provided with food containing only the solvent (2% EtOH). Heat shock treatment for Gal80 dysfunction in Gal80^ts^ mutants was performed in adult flies, which were placed in vials placed in a 29 °C incubator for 5 d, following which they were adapted to 25 °C for 1 d before analysis.

### Courtship behaviour assays

Naïve males with no pretested social experience were collected on the day of eclosion and kept in individual test tubes in a 25 °C incubator on a 12:12-h light:dark cycle for 5–8 days. Target males were stored in groups (25 males per vial). Courtship assays were performed between hours 2 and 6 of the light cycle every day. The paired courtship test, chaining test, and competitive courtship assay were conducted as previously described^88,89^. The courtship index (CI) was defined as the percentage of the 10-min recording period that the courter male spent courting the courtee. For the chaining test, a chain was defined as a group of at least three males exhibiting courtship behaviour with each other. The chaining index (ChI) was defined as the percentage of time that groups of males spent courting during a 10-min observation period. For the competitive courtship assay, the courtship indices toward females and males were simultaneously measured and then quantified by calculating preference index (PI) defined as [CI_female_–CI_male_]/[CI_female_ +  CI_male_].

### Genotypes

The genotypes used in this study are listed in Supplementary Data 1 along with the sample sizes used for the behavioural- and imaging-related analyses.

### Statistical analysis

Experimental flies and relative controls were employed at the same condition, and data are collected from at least two independent experiments. Statistical analyses were performed as indicated in the figure legends using the GraphPad Prism 9 software. Data distributions were assessed for normality using the Shapiro–Wilk test. If normally distributed, Student’s *t*-test was used for pairwise comparisons, while one-way analyses of variance (ANOVAs) were used for comparisons among multiple groups, followed by Tukey’s test for multiple comparisons. For data that sometimes deviated from a normal distribution, the Mann–Whitney U-test was used for pairwise comparisons, while the Kruskal–Wallis test was used for comparisons among multiple groups, followed by Dunn’s test for multiple comparisons.

### Additional methods

Details of the protocols used for inverse PCR, transgenic fly generation, immunohistochemistry, chemical labelling, in vivo calcium imaging, pharmacological manipulation, quantitative measurement of 20E, motor activity analysis, analyses of lifespan and tolerance to starvation stress, quantitative RT-PCR, ELISA, PLA, and image analysis are provided in the Supplementary Online Methods.

### Reporting summary

Further information on research design is available in the Nature Research Reporting Summary linked to this article.

## Supplementary information

Supplemental Information

Description to Supplementary Information

Supplementary Data 1

Supplementary Data 2

Supplementary Data 3

Supplementary Data 4

Supplementary Movie 1

Supplementary Movie 2

Supplementary Movie 3

Reporting Summary

## Data Availability

All data generated or analysed during this study are included in this published article (and its supplementary information files). The raw data are provided as a Source Data file and available from the corresponding author upon request.
