# Supplementary material for: WAKE-mediated modulation of cVA perception via a hierarchical neuro-endocrine axis in Drosophila male-male courtship behaviour
Source: Nat Commun. 2022 May 6;13:2518. doi: 10.1038/s41467-022-30165-2 (PMC9076693; doi:10.1038/s41467-022-30165-2)
Supplement: Supplementary file 1 — Supplemental Information [file 41467_2022_30165_MOESM1_ESM.pdf]

**Supplemental Information**

# WAKE-mediated modulation of cVA perception via a hierarchical neuro-endocrine axis in *Drosophila* male-male courtship behaviour

Shiu-Ling Chen, Bo-Ting Liu, Wang-Pao Lee, Sin-Bo Liao, Yao-Bang Deng, Chia-Lin Wu, Shuk-Man Ho, Bing-Xian Shen, Guan-Hock Khoo, Wei-Chiang Shiu, Chih-Hsuan Chang, Hui-Wen Shih, Jung-Kun Wen, Tsuo-Hung Lan, Chih-Chien Lin, Yu-Chen Tsai*, Huey-Fen Tzeng*, Tsai-Feng Fu*

*Corresponding authors. Email addresses: tffu@ncnu.edu.tw, hftzeng@ncnu.edu.tw, yctsaibio@thu.edu.tw

Supplementary Results
Figure 1
Figure 2
Figure 3
Figure 4
Figure 5
Figure 6
Figure 7
Figure 8
Figure 9
Figure 10
Figure 11
Figure 12
Figure 13, related to Supplementary Table 1
Figure 14
Figure 15

Table 1, related to Supplementary Figure 13
Table 2
Table 3

Supplementary Methods

Supplementary References

## Supplementary Results

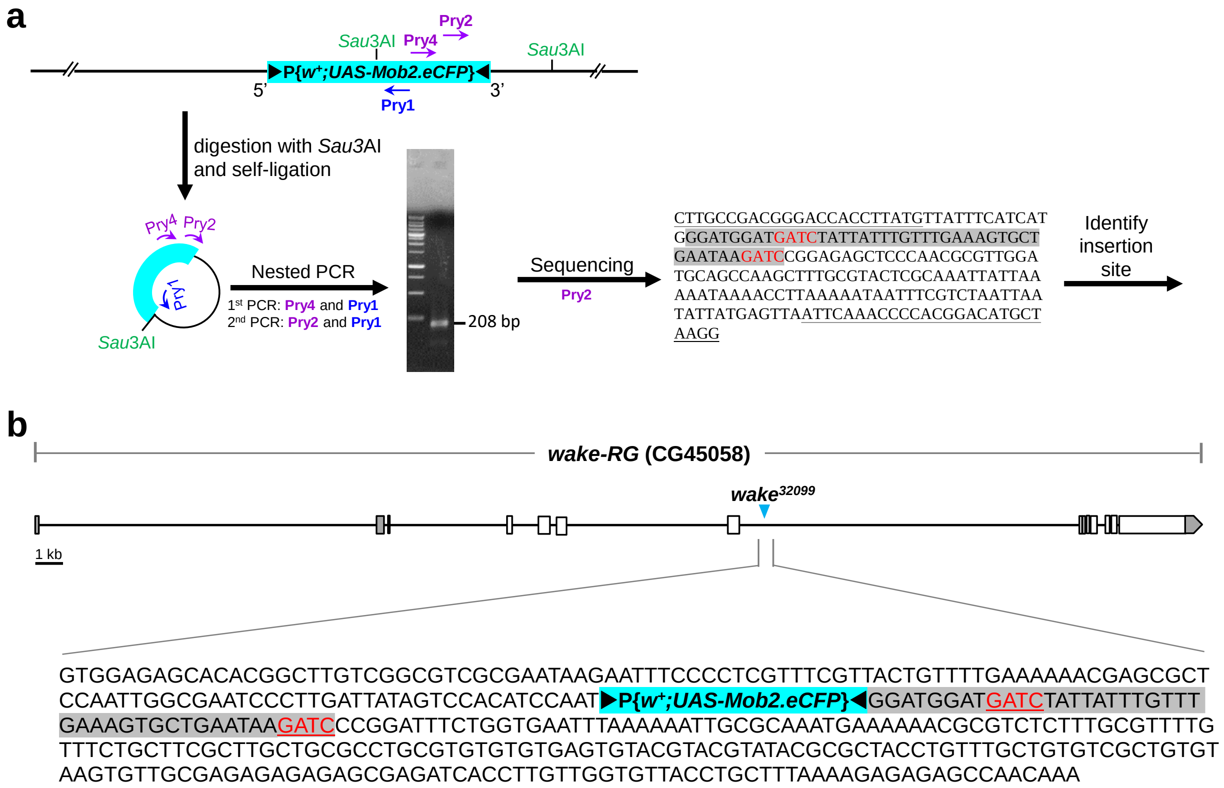


**Supplementary Figure 1. Schematic of PCR methods for mapping p{*w*$^+$;*UAS-Mob2.eCFP*} transposable element.** (**a**) Genomic DNA isolated from *wake*$^{32099}$ containing a p{*w*$^+$; *UAS-Mob2.eCFP*} transposable element is indicated as a blue bar, which is progressively digested with *Sau*3AI and circularized by a self-ligation reaction. A nested-PCR reaction with primers (Pry1, 2, 4) designed to the transposon end and an internal sequence amplify the flanking genomic region. This PCR product was sequenced using a nested primer. (**b**) p{*w*$^+$; *UAS-Mob2.eCFP*} in the *wake* gene in *wake*$^{32099}$ is characterized and represented as sequences of the junction sites within *Sau*3AI. The restriction enzyme recognizes GATC sites (printed in red).

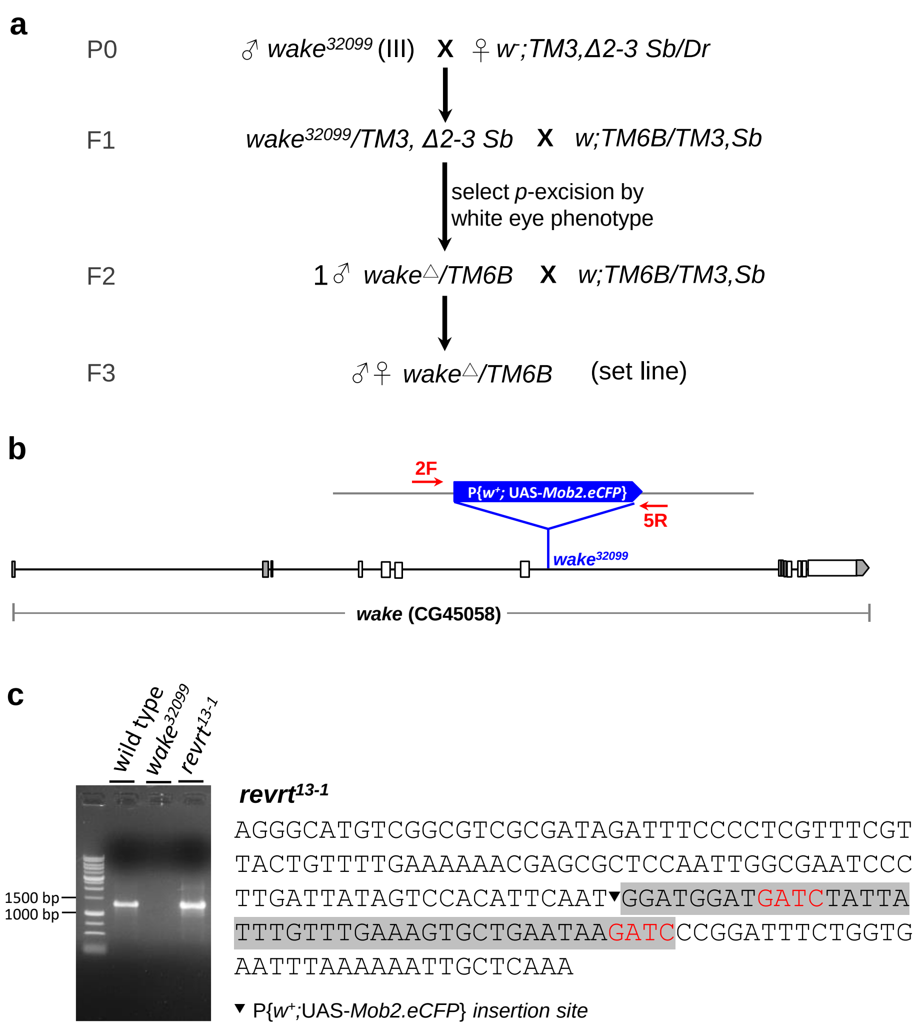


**Supplementary Figure 2. Generation of revertant by P-excision.** (**a**) Schematic representation of P-excision. *wake*$^{32099}$ contains a P{*w*$^{+}$*;UAS-Mob2.eCFP*} transposable element (blue bar). The P-element of *wake*$^{32099}$ was mobilized by introducing transposase △2-3, following which P-excision flies were selected based on the white-eye phenotype and set lines. (**b**) Relative positions and orientations of genomic PCR primers (2F, 5R; red) were used to confirm the precision excision. (**c**) PCR fragments amplified from wild-type, *wake*$^{32099}$ and named *revrt*$^{13\text{-}1}$ lines are shown in the left panel. The right panel shows corresponding PCR fragments amplified from the *revrt*$^{13\text{-}1}$ line, characterized and represented as the sequences of the junction sites within *Sau*3AI. The restriction enzyme recognizes GATC sites (printed in red). The sequence analysis indicated that the *revrt*$^{13\text{-}1}$ line was a precise excision line.

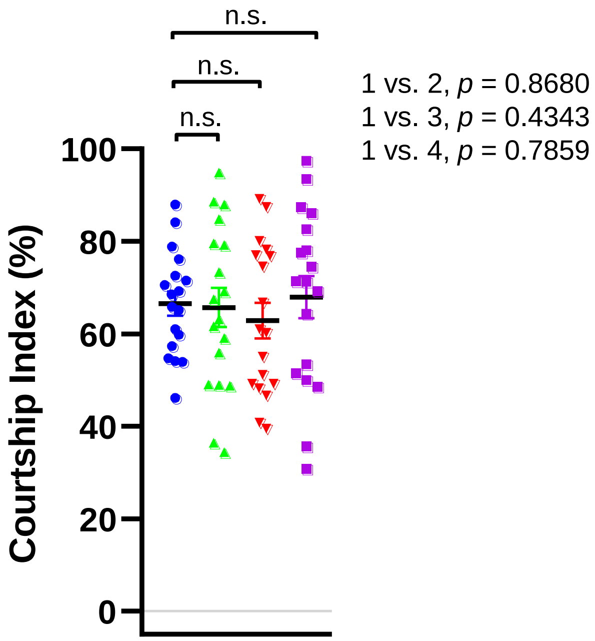


| Test group no. | | 1 | 2 | 3 | 4 |
|---|---|---|---|---|---|
| Courter male | *wake*$^{32099}$ | ● | ● | ● | ● |
| Courtee male (strain) | 2U | ● | | | |
| | w1118 | | ● | | |
| | Canton-S | | | ● | |
| | Oregon-R | | | | ● |

**Supplementary Figure 3. WAKE-deficient males were tested toward males with different genetic backgrounds.** Scatterplots for Courtship Index (n = 18 for each) with error bars indicating ± SEM for all data points. Test group no. 1–4, $p > 0.05$ (n.s.), two-tailed unpaired *t*-test. Source data are provided as a Source Data file.

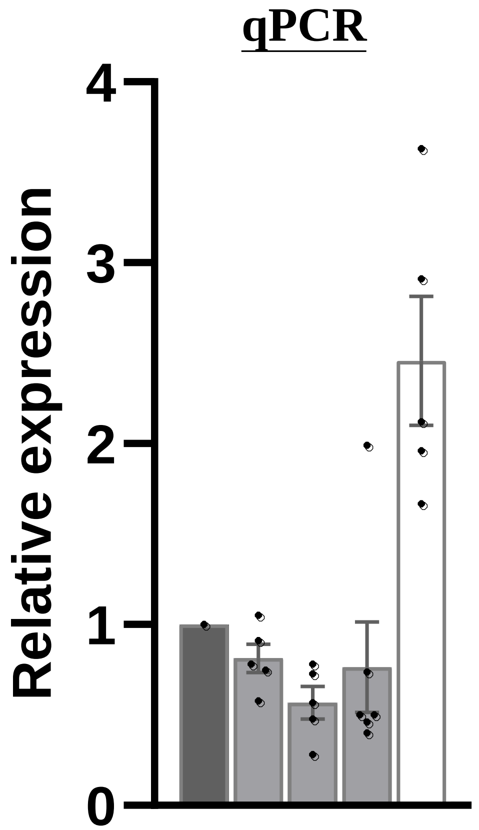


| Test group no. | 1 | 2 | 3 | 4 | 5 |
|---|---|---|---|---|---|
| *elav-Gal4* | ● | ● | ● | ● | ● |
| *UAS-wake*$^{RNAi}$ -1 | | ● | | | |
| *UAS-wake*$^{RNAi}$ -2 | | | ● | | |
| *UAS-wake*$^{RNAi}$ -3 | | | | ● | |
| *UAS-wake-RG*$_{HA}$ | | | | | ● |

**Supplementary Figure 4. qRCR validation of the efficiency of *wake* dsRNA silencing and WAKE overexpression.** Quantitative RT-PCR confirmed altered expression of *wake* transcripts in *UAS-wake*$^{RNAi}$ and *UAS-wake-RG::HA* derived *elav-Gal4* males. Fold changes were calculated using ΔΔCt values relative to the reference gene *rpl32* and the corresponding *elav-Gal4* heterozygous controls, n = 5, 5, 5, 6 and 5 (from left to right) for test group no. 1–5. Scatterplots with bar include ± SEM for all data points. Source data are provided as a Source Data file.

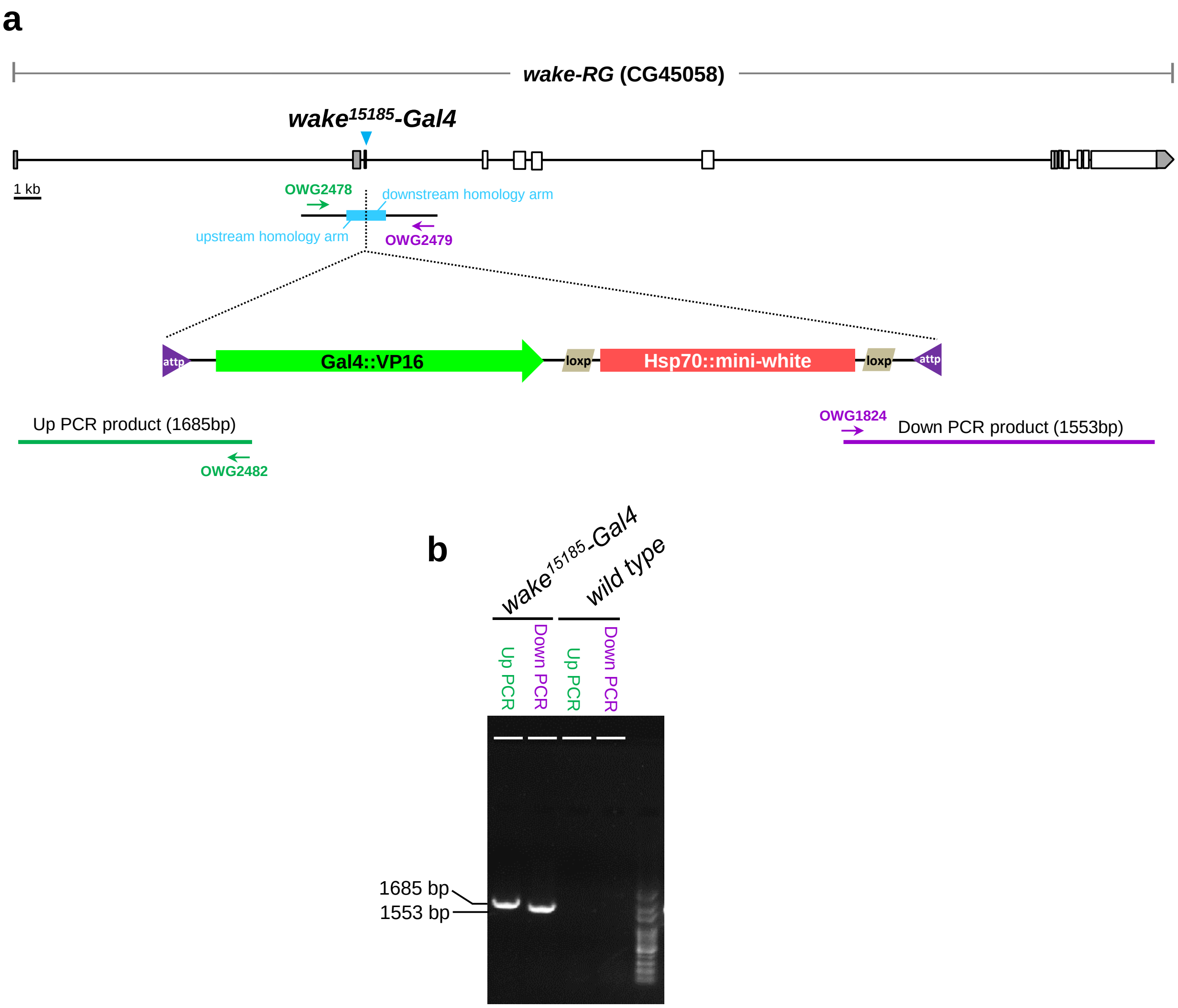


**Supplementary Figure 5.** (**a**) The seven isoforms of *wake* in FlyBase. Using *wake-RG* as the reference, bases 12596 to 12589 before ATG were deleted (a 7 bp deletion) and replaced with the RMCE-Gal4-w knock-in cassette (for the detailed nucleotide sequence, see Supplementary Data 2), consisting of an attP, a Gal4::VP16, a loxed hsp70::*mini-white*, and an inverted attP. (**b**) PCR products were analyzed using agarose gel electrophoresis for knock-in detection. Knock-in fly genomic DNA was isolated and subjected to locus-specific PCR amplification. A PCR reaction with two primer pairs (OWG2478 and OWG2482, green arrows; OWG2479 and OWG1824, purple arrows) designed to the knock-in site and an internal knock-in cassette sequence were used to amplify the flanking genomic region.

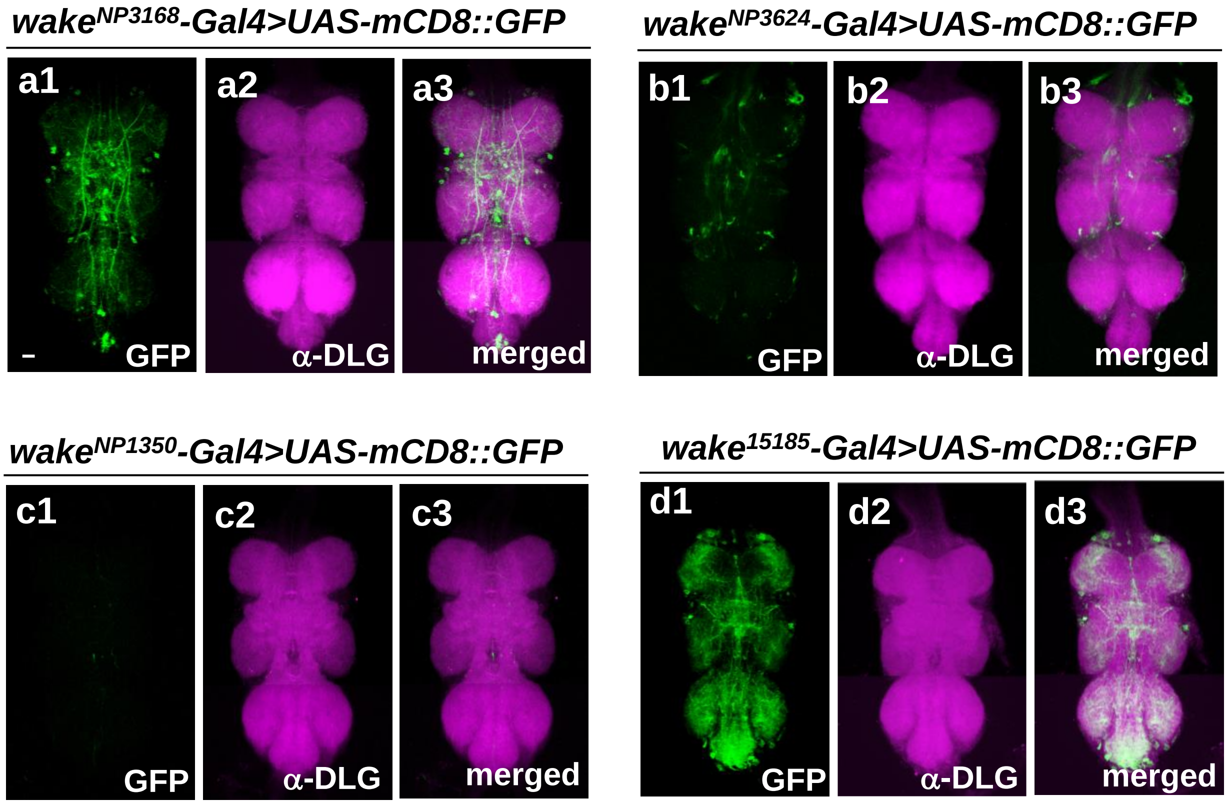


**Supplementary Figure 6. The expression patterns of *wake-Gal4* drivers in the ventral nerve cord (VNC) of male *Drosophila*.** Representative images showing the expression patterns of four *Gal4* drivers in the VNC. (**a**) $wake^{NP3168}$, (**b**) $wake^{NP3624}$, (**c**) $wake^{NP1350}$, and (**d**) $wake^{15185}$-*Gal4* expression patterns revealed using *UAS-mCD8::GFP* in the adult male brain (green in **a1**–**d1**). The neuropil was immunostained using an anti-DLG antibody (magenta in **a2**–**d2**); merged in **a3**–**d3**, n = 6 for each. Scale bars, 20 µm.

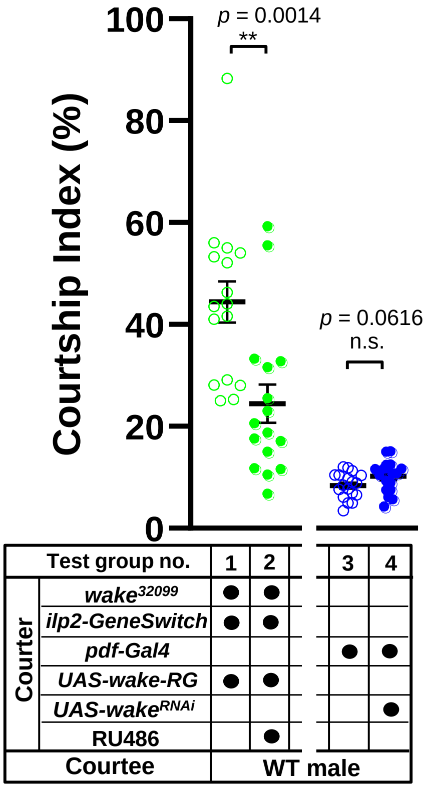


**Supplementary Figure 7. WAKE in IPCs can suppress male–male courtship behaviour but is not involved in modulating male–male courtship behaviour in PDF neurons.** Comparison of RU486-treated 5-day-old males (*ilp2-GeneSwitch>UAS-wake-RG*) with an untreated control in the *wake*$^{32099}$ genetic background. Significantly different courtship indices were observed. *pdf-Gal4* driven *wake* dsRNA were used to downregulate WAKE in PDF-expressing neurons; however, no significant difference was observed in the corresponding controls. n = 16 and 16 for test group no. 1 and 2, $^{**}P < 0.01$, two-tailed Mann–Whitney U-test. n = 18, and 18 for test group no. 3 and 4, $p > 0.05$ (n.s.), two-tailed unpaired $t$-test. Error bars indicate ± SEM. Source data are provided as a Source Data file.

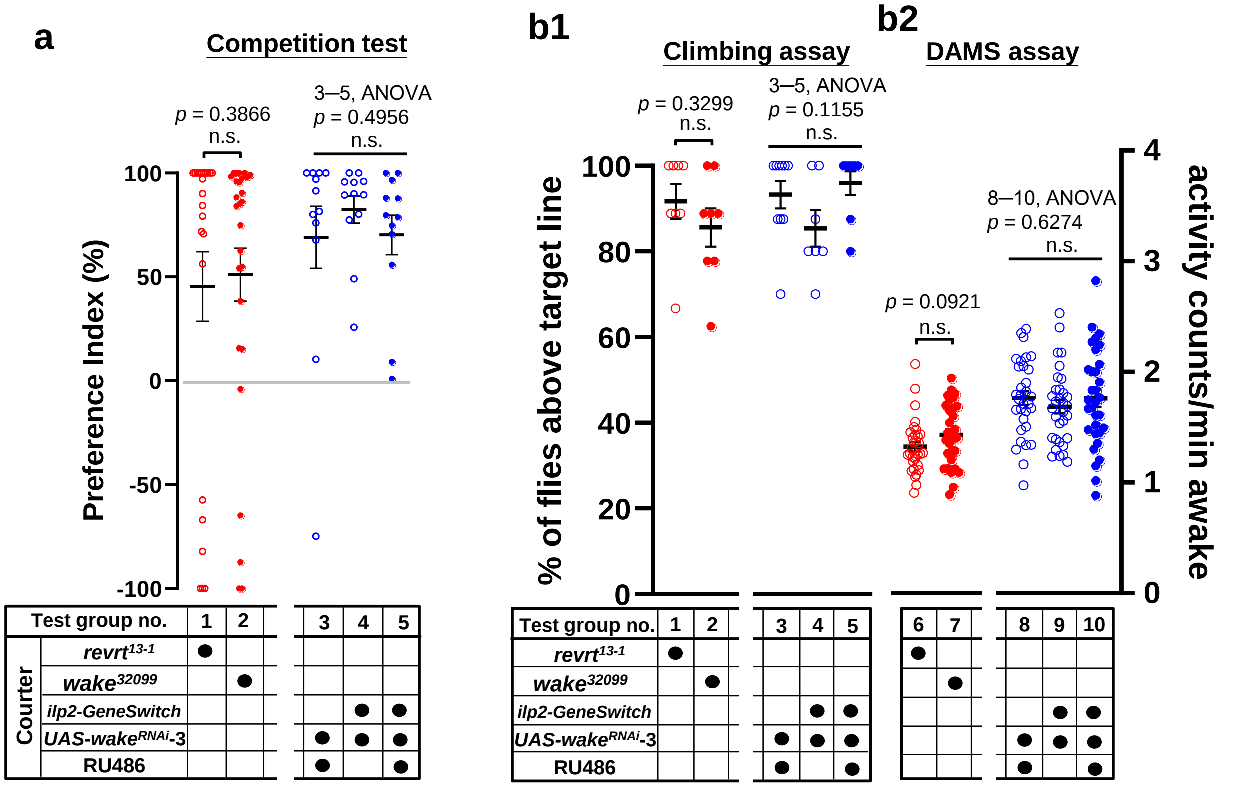


**Supplementary Figure 8. Courtship sustainment and motor activity in WAKE-deficient *Drosophila* male.** (**a**) Courtship competition tests indicated that the preference indices in 6-day-old *wake*$^{32099}$ or RU486-treated males (*ilp2-GeneSwitch*>*UAS-wake*$^{RNAi}$) were significantly higher toward females than that toward males when compared with those in the corresponding controls. n = 23 and 27 for test group no. 1 and 2. $p > 0.05$ (n.s.), two-tailed Mann–Whitney U-test. n = 12, 12 and 12 for test group no. 3–5, $p = 0.4956$ (n.s.), Kruskal–Wallis test. (**b1**) Motor activity was assessed using the negative gravitaxis assay to ensure that differences were not due to motor impairments. The number of flies that climbed up to a vertical distance of > 8 cm was recorded at the end of each trial. WAKE-deficient males exhibited no significant difference in climbing performance irrespective of *wake*$^{32099}$ background or RU486 treatment (*ilp2-GeneSwitch*>*UAS-wake*$^{RNAi}$) when compared with the corresponding controls. n = 8 and 8 for test group no. 1 and 2, $p > 0.05$ (n.s.), two-tailed unpaired *t*-test. n = 10, 7 and 8 for test group no 3–5, $p > 0.05$ (n.s.), Kruskal–Wallis test. (**b2**) Spontaneous motor activities in WAKE-deficient male flies were recorded using the *Drosophila* activity monitoring system (DAMS; TriKinetics, Waltham, MA) for 24 h in each fly. During the waking state, there was no significant difference in average count per minute between the WAKE-deficient males and the corresponding controls. n = 32, 32 for test group no. 1 and 2, $p > 0.05$ (n.s.), two-tailed Mann–Whitney U-test. n = 32, 32, 32 for test group no 3–5, $p > 0.05$ (n.s.), one-way ANOVA using F-test, $F(2, 93)=0.4684$, $p = 0.6274$. Error bars indicate ± SEM. Source data are provided as a Source Data file.

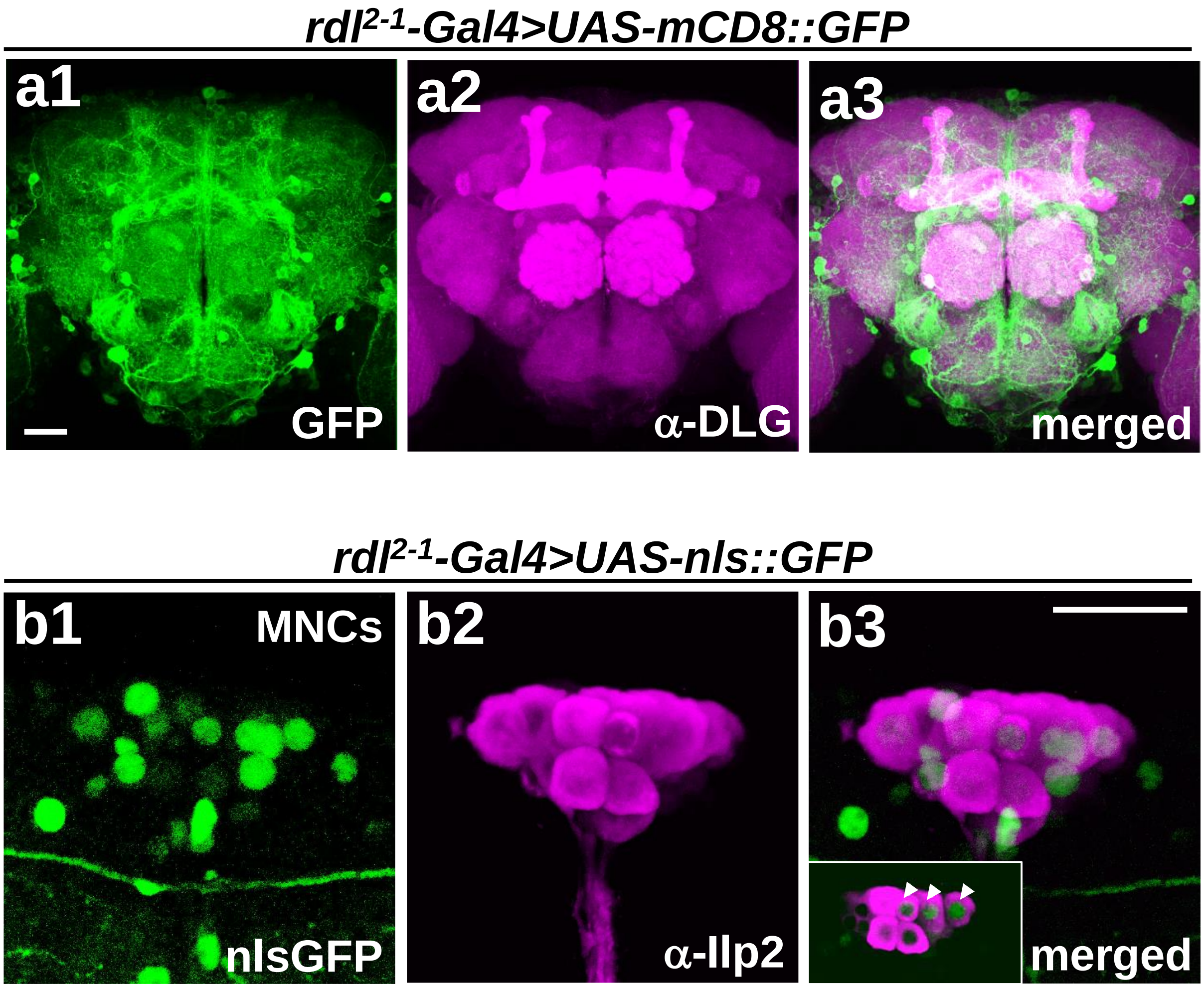


**Supplementary Figure 9. Expression pattern of the *rdl*$^{2\text{-}1}$*-Gal4* driver in male brains.** (**a**) GFP expression driven by *rdl*$^{2\text{-}1}$*-Gal4* (green in **a1**). The neuropil was immunostained using an anti-DLG antibody (magenta in **a2**); merged in **a3** (n = 6 for each). (**b**) Representative image showing that the cell bodies of medium neurosecretory cells (MNCs) are driven by *rdl*$^{2\text{-}1}$*-Gal4* due to *UAS-nls::GFP* for the nuclei (green in **b1**) and the Ilp2-immunolabeled IPCs (magenta in **b2)**; merged in **b3** (n = 8 for each). The embedded image in **b3** shows a single section after superposition, with arrowheads indicating the representative co-localization signals. Scale bar, 20 μm.

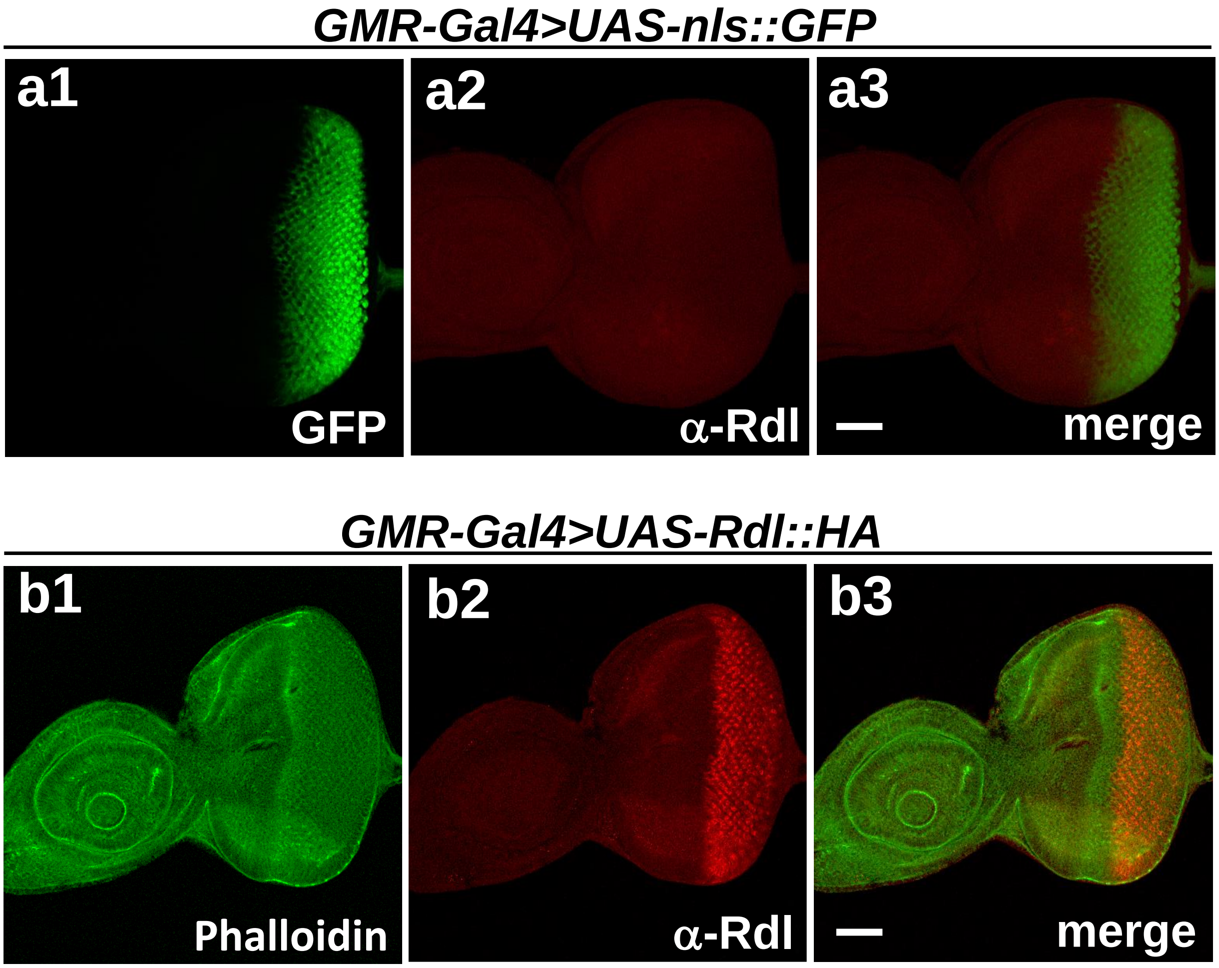


**Supplementary Figure 10. Ectopic expression of Rdl::HA in eye discs can be identified for immunolabeling using the generated Rdl antibody.** (**a**) Eye discs with an expression of nlsGFP under the *GMR-Gal4* (green in **a1**). A negative test result implies that the anti-Rdl antibody was not detected in this specimen (**a2**), and merged in **a3** (n = 10 for each). (**b**) Rdl protein (Red) was detected in eye disc cells with expression of Rdl::HA under the *GMR-Gal4* (**b2**), demonstrating the specificity of anti-Rdl antibody; (**b1**) Phalloidin (Green) was used to mark the overall shape of the tissue and merged in **b3** (n = 10 for each). Scale bar, 50 μm.

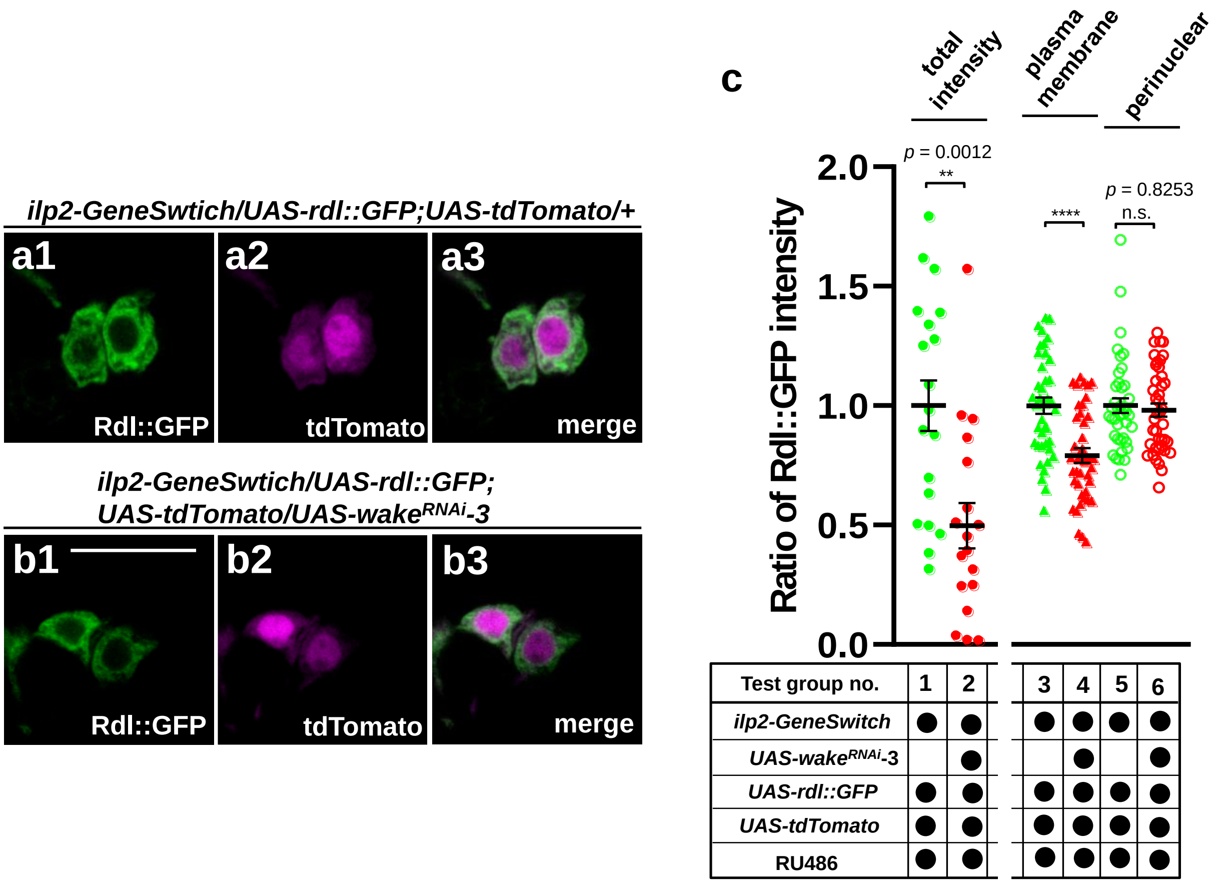


**Supplementary Figure 11**. **WAKE affects Rdl levels and trafficking in the IPCs**. Quantification of total and plasma membrane versus perinuclear Rdl::GFP levels in the IPCs were analyzed on (**a**) normal and (**b**) WAKE-deficiency backgrounds. The expression of Rdl::GFP was driven by *ilp2-GeneSwitch* due to RU486 induction (green in **a1** and **b1**), and anatomical landmarks of IPCs was illustrated by tdTomato (magenta in **a2** and **b2**) and merged in **a3** and **b3** (n = 10 for each). Scale bar denotes 20 μm. There were significant differences in the total and plasma membrane intensity of Rdl::GFP when compared with that in flies with WAKE downregulation in IPCs. Data quantified in (**c**); n = 19, 18, 40, and 41 for test group no. 1–4, $^{**}p < 0.01$ and $^{****}p < 0.001$, two-tailed unpaired *t*-test. n = 40, and 41 for test group no. 5 and 6, $p > 0.05$ (n.s.), two tailed Mann–Whitney U-test. Error bars indicate ± SEM. Source data are provided as a Source Data file.

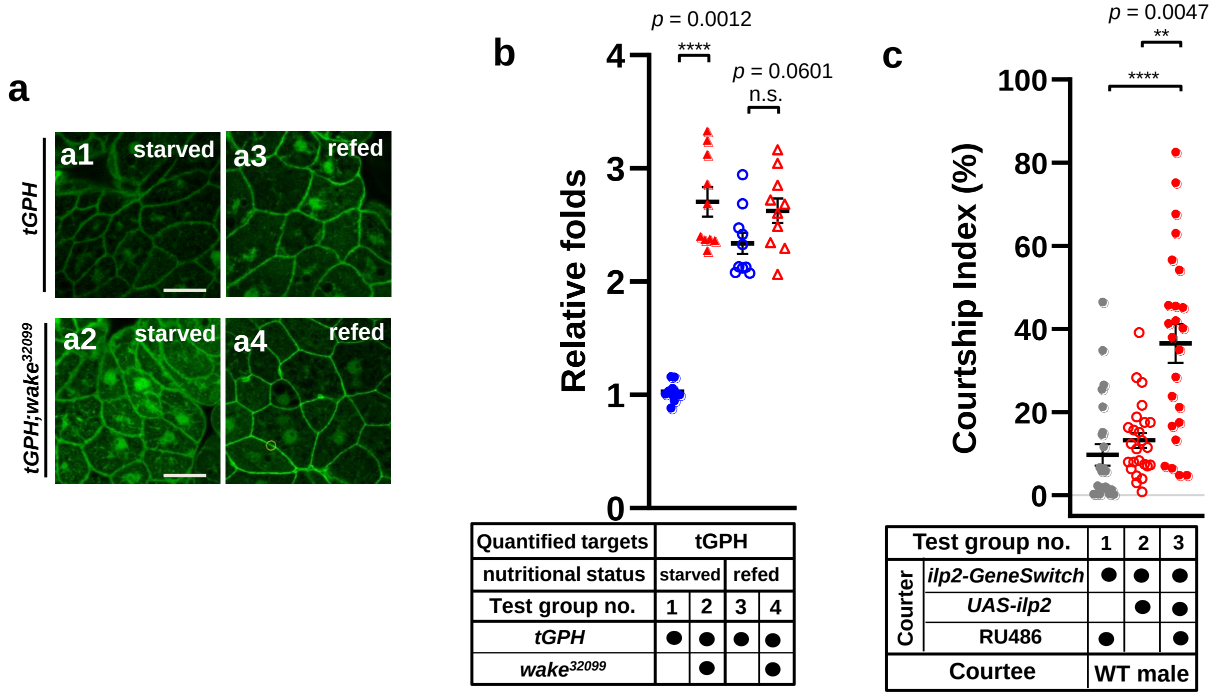


**Supplementary Figure 12. WAKE affects IIS and direct overexpression of Ilp2 in IPCs is sufficient to induce male-male courtship behaviour.** (**a**) Membrane localisation of *tGPH* was observed in the fat body of $wake^{32099}$ flies. These signals (green) were significantly lower in $wake^{32099}$ flies than in normal controls, suggesting that PI3K signalling is activated by IIS (**a1, 2**) (n = 10 for each). The membrane signals responded normally to feeding for 30 min (**a3, 4**) (n = 10 for each); quantification in (**b**); n = 10 for each, $^{****}p < 0.001$ and $p > 0.05$ (n.s.), two-tailed unpaired *t*-test. (**c**) Expression of Ilp2 was induced in IPCs after eclosion using an *ilp2-GeneSwitch* driver and RU486 treatment. Over-expression of Ilp2 significantly induced male-male courtship behaviour when compared with that in corresponding RU486-untreated controls. n = 24, 25 and 24 (from left to right) for each test group. $p < 0.05$, Kruskal–Wallis test. $^{***}p < 0.005$ and $^{****}p < 0.001$, post hoc Dunn's multiple comparisons test. Scatterplots include ± SEM. Source data are provided as a Source Data file. Scale bars, 20 µm.

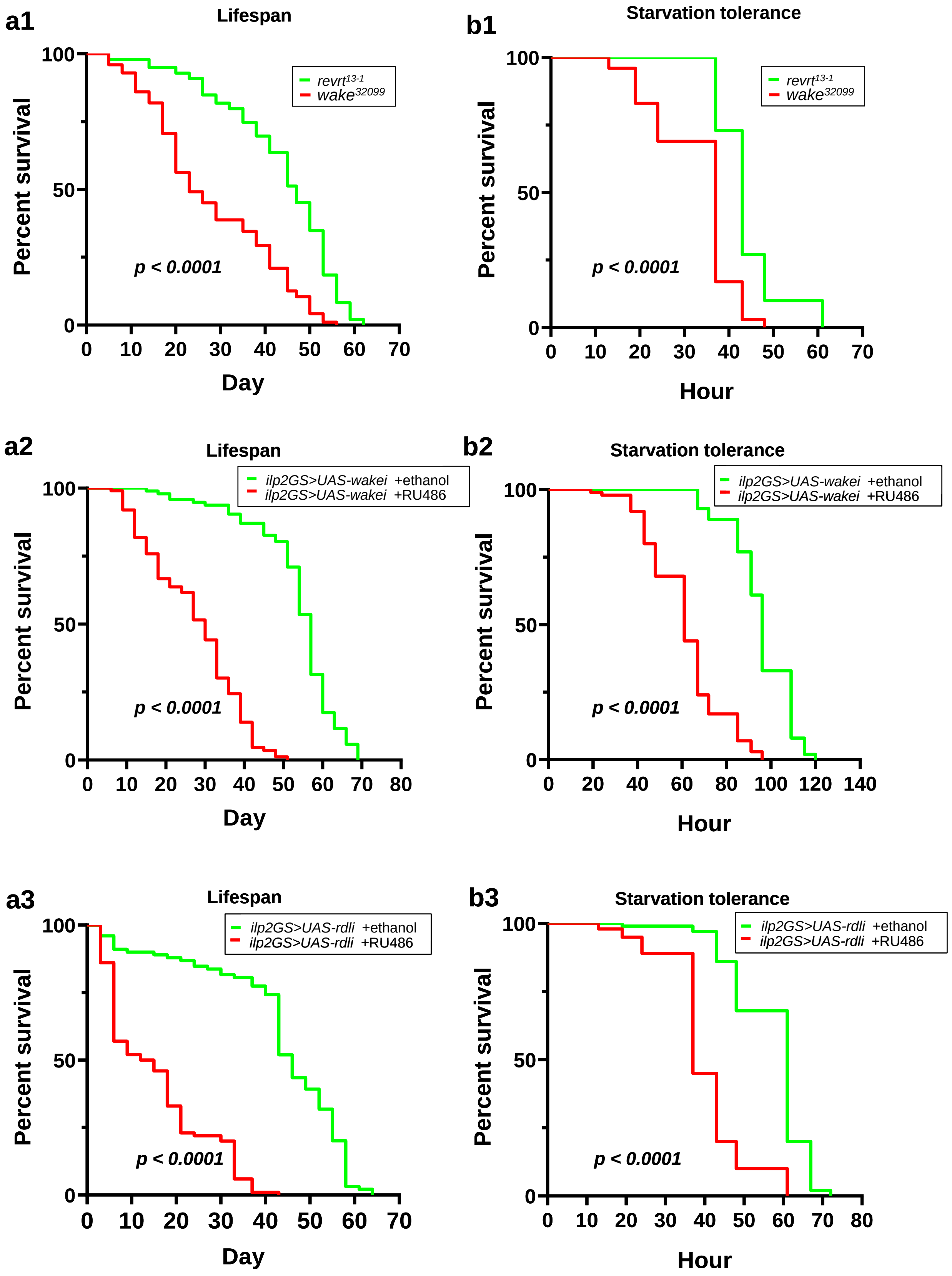
a1
Lifespan
revrt13-1
wake32099
p < 0.0001
Percent survival
Day
b1
Starvation tolerance
revrt13-1
wake32099
p < 0.0001
Percent survival
Hour
a2
Lifespan
ilp2GS>UAS-wakei +ethanol
ilp2GS>UAS-wakei +RU486
p < 0.0001
Percent survival
Day
b2
Starvation tolerance
ilp2GS>UAS-wakei +ethanol
ilp2GS>UAS-wakei +RU486
p < 0.0001
Percent survival
Hour
a3
Lifespan
ilp2GS>UAS-rdli +ethanol
ilp2GS>UAS-rdli +RU486
p < 0.0001
Percent survival
Day
b3
Starvation tolerance
ilp2GS>UAS-rdli +ethanol
ilp2GS>UAS-rdli +RU486
p < 0.0001
Percent survival
Hour

**Supplementary Figure 13. WAKE or Rdl in IPCs of adult males is necessary to maintain the life span and survival in starved flies.** Differences in lifespan (**a1-3**) and starvation tolerance (**b1-3**) in *wake*$^{32099}$ males (red in a1, b1) and in those in which *ilp2-GeneSwitch* driven *wake* or *rdl* dsRNA was used to downregulate *wake* (red in **a2, b2**) or *rdl* (red in **a3, b3**), respectively, in adult IPCs after RU486 treatment. All values were lower than those for a revertant (*revrt*$^{13-1}$) or an RU486-untreated control male (green), respectively. *P* values were obtained based on the log-rank test. Source data are provided as a Source Data file.

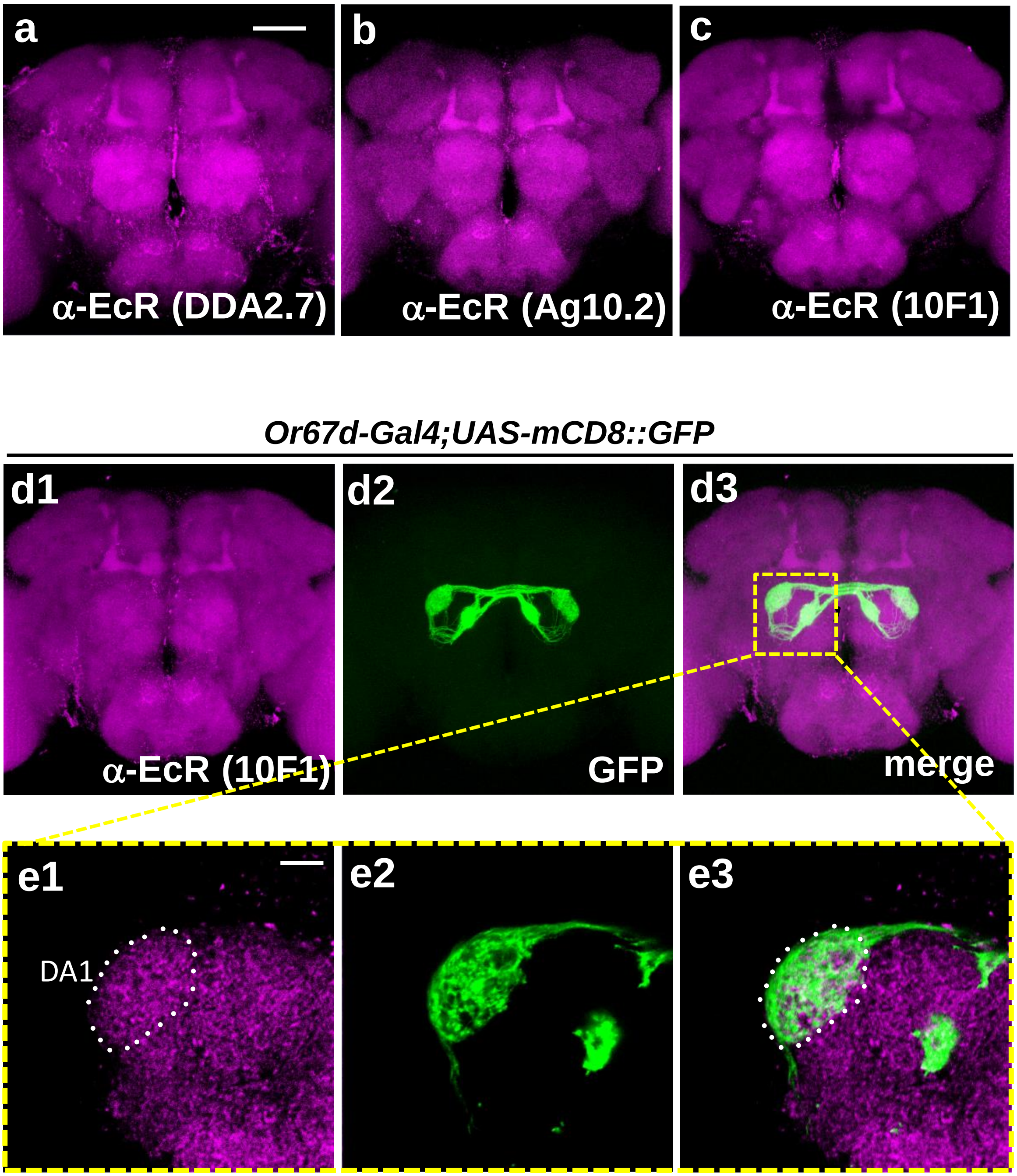


**Supplementary Figure 14. Immunohistochemical detection of EcRs in the adult brain.** Expression of *UAS-mCD8::*GFP controlled by the *Or67d-Gal4* driver. EcR localization in the adult brain was performed using (**a**) DDA2.7, (**b**) Ag10.2, or (**c**)10F1 (shown as magenta). Anterior views of the whole adult brain are shown, respectively. (**d1-3**) Visualization of the GFP expressing OR67d neurons (*Or67d-Gal4>UAS-mCD8::GFP*) in the adult brain (green) and immunolabelling of EcR with anti-EcR antibody 10F1 (magenta). (**e1-3**) Representative zoomed-in images showing an Or67d-expressing OSN that projects to the DA1 glomerulus (white dotted circles) and EcR-immunolabeled signals. n = 6 for each test. Scale bar, 20 μm.

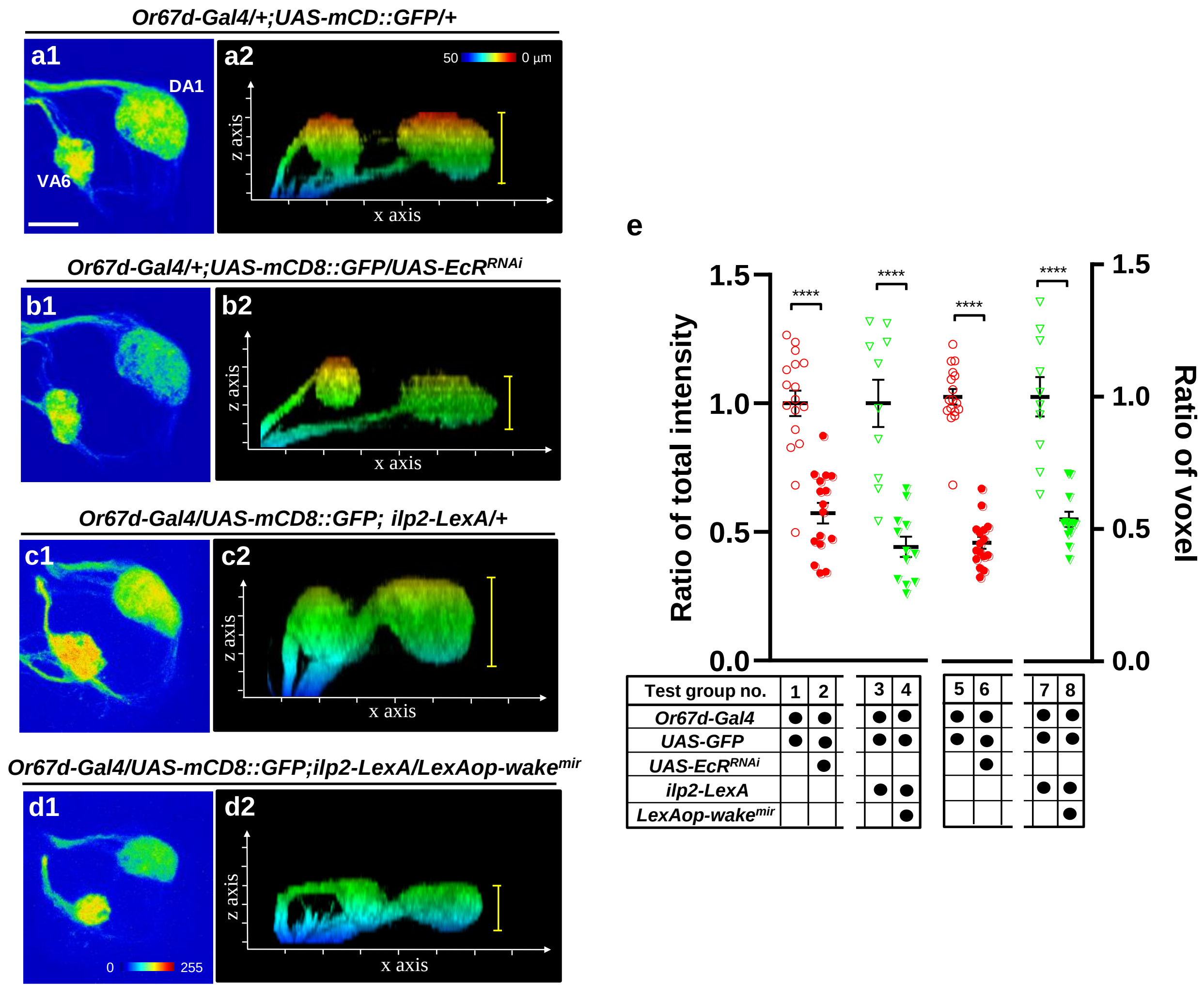


**Supplementary Figure 15. Effect of WAKE and ecdysone signalling on the morphological variation of Or67d neurons evaluated based on volume and neural fibre density in the DA1 olfactory glomerulus.** High magnification confocal stacks of a single mCD8::GFP-labelled DA1 glomerulus in the brain of an adult male (8 d-old) (*Or67d-Gal4*>*UAS-mCD8::GFP*). (**a1-d1**) There were significant differences in the intensity of GFP signals when compared with those in flies simultaneously expressing $EcR^{RNAi}$ or with WAKE downregulation in IPCs, n = 17, 16, 10 and 12; Scale bar, 20 µm. (**a2-d2**) Screenshots of three-dimensional renderings turn to show Z-stack images. There were significant differences in the thickness in the z-axis when compared with that in flies simultaneously expressing $EcR^{RNAi}$ or with WAKE downregulation in IPCs, n = 17, 16, 10 and 12. Data quantified in (**e**); n = 17, 16, 10, 12, 17, 16, 10, and 12 (from left to right) for test group no. 1–8. $^{****}p < 0.001$, two-tailed unpaired *t*-test. A scatterplot with error bars indicating ± SEM. Source data are provided as a Source Data file.

**Supplementary Tables**

**Supplementary Table 1. The effects of WAKE- or Rdl-deficiency in IPCs on lifespan and starvation resistance in adult *Drosophila* males**

| test | fly | Sample size (N=) | mean of lifespan (day/h) | shorten (%) |
|---|---|---|---|---|
| lifespan | wake$^{32099}$ | 97 | 28.15 d | -35.41 |
| | revrt$^{13-1}$ | 98 | 43.59 d | |
| | ilp2-GeneSwitch>UAS-wake$^{RNAi}$ RU486 (+) | 87 | 27.43 d | -48.9 |
| | ilp2-GeneSwitch>UAS-wake$^{RNAi}$ RU486 (-) | 94 | 53.68 d | |
| | ilp2-GeneSwitch>UAS-rdl$^{RNAi}$ RU486 (+) | 95 | 15.63 d | -65.22 |
| | ilp2-GeneSwitch>UAS-rdl$^{RNAi}$ RU486 (-) | 100 | 44.95 d | |
| starvation stress tolerence | wake$^{32099}$ | 100 | 33.05 h | -24.94 |
| | revrt$^{13-1}$ | 100 | 44.03 h | |
| | ilp2-GeneSwitch>UAS-wake$^{RNAi}$ RU486 (+) | 100 | 61.67 h | -35.6 |
| | ilp2-GeneSwitch>UAS-wake$^{RNAi}$ RU486 (-) | 100 | 95.76 h | |
| | ilp2-GeneSwitch>UAS-rdl$^{RNAi}$ RU486 (+) | 100 | 40.20 h | -29.57 |
| | ilp2-GeneSwitch>UAS-rdl$^{RNAi}$ RU486 (-) | 100 | 57.08 h | |

**Supplementary Table 2. Fly strains used in this study and their sources**

| Classification | Genotypes | Sources | Number |
|---|---|---|---|
| Gal4 lines | | | |
| wake$^{NP3168}$ | w*; P{w$^{+mW.hs}$=GawB}NP3168 | DGRC | #113136 |
| wake$^{NP3624}$ | w*; P{w+mW.hs=GawB}NP3624 | DGRC | #104560 |
| wake$^{NP1350}$ | y* w*; P{w+mW.hs=GawB}NP1350 / TM6, P{w-=UAS-lacZ.UW23- | DGRC | #112640 |
| wake$^{15185}$-Gal4 | - | This study | - |
| iIp2-Gal4 | - | PY Wang[1] | #104560 |
| rdl$^{2-1}$-Gal4 | - | J Simpson[2] | - |
| Aug21-Gal4 | w*; P{w[+mW.hs]=GawB}Aug21/CyO | BDSC | #30137 |
| fruP1-Gal4 | w*; TI{GAL4}fru[GAL4.P1.D]/TM3, Sb[1] | BDSC | #66696 |
| Or67d-Gal4 | - | AS Chiang[3] | - |
| Jhamt-Gal4 | - | L Sheng[4] | - |
| pdf-Gal4 | - | AS Chiang[3] | - |
| elav-Gal4 | - | CL Wu[5] | - |
| GMR-Gal4 | w[*]; P{w[+mC]=GMR-GAL4.Y}YH3/TM3, Sb[1] | BDSC | #84247 |
| GeneSwitch lines | | | |
| actin-GeneSwitch | w; P{Act5C(-FRT)GAL4.Switch.PR}255A | J Tower[6] | - |
| iIp2-GeneSwitch | - | PY Wang[1] | - |
| UAS lines | | | |
| UAS-wake$^{RNAi}$-1 | P{KK111313}VIE-260B | VDRC | #102272 |
| UAS-wake$^{RNAi}$-2 | P{KK112014}VIE-260B | VDRC | #102564 |
| UAS-wake$^{RNAi}$-3 | w$^{1118}$; P{GD12087}v27760 | VDRC | #27760 |
| UAS-wake-RG$_{HA}$ | - | This study | - |
| UAS-mCD8::GFP | P{UAS-mCD8::GFP.L}LL5;P{UAS-mCD8::GFP.L}LL6 | BDSC | #5137;5130 |
| UAS-nlsGFP | w$^{1118}$; P{w[+mC]=UAS-GFP.nls}8 | BDSC | #4776 |
| UAS-Ilp2 | | PY Wang[1] | |
| UAS-Rdl::GFP | w[*]; P{y[+t7.7] w[+mC]=20XUAS-Rdl.GFP}su(Hw)attP5/CyO | BDSC | #92150 |
| UAS-tdTomato | w[*]; P{w[+mC]=UAS-tdTom.S}3 | BDSC | #36328 |
| UAS-Rdl$_{HA}$ | w[*]; P{w[+mC]=UAS-Rdl.HA}C; MKRS/TM6B | BDSC | #29038 |
| UAS-rdl$^{RNAi}$-1 | w$^{1118}$; P{GD4609}v41101/CyO | VDRC | #41101 |
| UAS-rdl$^{RNAi}$-2 | y$^{1}$ v1; P{TRiP.JF01455}attP2 | BDSC | #31662 |
| UAS-rdl$^{RNAi}$-3 | y$^{1}$ v1; P{TRiP.JF01227}attP2 | BDSC | #31286 |

| Classification | Genotypes | Sources | Number |
|---|---|---|---|
| Gal4 lines | | | |
| UAS-rdl | w[*] P{w[+mC]=UAS-Rdl.S}8.2; sna[Sco]/CyO | BDSC | #29036 |
| UAS-InR$^{DN}$ | - | PY Wang[1] | - |
| UAS-PI3K$^{DN}$ | y[1] w[1118]; P{w[+mC]=UAS-Pi3K92E.A2860C}1 | BDSC | #8288 |
| UAS-chico$^{RNAi}$ | P{KK107581}VIE-260B | VDRC | #101329 |
| UAS-InR$^{CA}$ | - | PY Wang[1] | - |
| UAS-jhamt$^{RNAi}$ | P{KK103237}VIE-260B | VDRC | #103958 |
| UAS-hmgcr$^{RNAi}$ | P{KK101807}VIE-260B | VDRC | #106817 |
| UAS-EcR$^{RNAi}$ | w$^{1118}$; P{GD1428}v37059 | VDRC | #37059 |
| UAS-EcR.A | w*; P{w[+mC]=UAS-EcR.A}3a | BDSC | #6470 |
| UAS-Jhamt | - | D Yamamoto[7] | - |
| UAS-brp::GFP | w*; P{w[+mC]=UAS-Brp.GFP}TR722 | BDSC | #36291 |
| UAS-GCamp6 | w$^{1118}$; P{y[+t7.7] w[+mC]=20XUAS-IVS-GCaMP6m}attP40 | CL Wu[5] | - |
| LexA line | | | |
| iIp2-LexA | - | ZF Gong[8] | - |
| LexAop lines | | | |
| LexAop-wake$^{mir}$ | P{LexAop-wake-mir1} | This study | - |
| Wild type | | | |
| 2U | - | AS Chiang[3] | - |
| w1118 | - | Fly Core[9] | - |
| Canton-S | - | Fly Core[9] | - |
| Oregon-R | - | Fly Core[9] | - |
| Others | | | |
| wake$^{32099}$ | P{UAS-Mob2.eCFP}3 | BDSC | #32099 |
| wake$^{GS17103}$ | y$^{1}$ w$^{67c23}$; P{w$^{+mC}$=GSV6}GS17103 / TM3, Sb$^{1}$ Ser$^{1}$ | DGRC | #206904 |
| revrt$^{13-1}$ | - | This study | - |
| Ilp2HF | - | S. Park[10] | - |
| Ilp2,3,5 | w$^{1118}$; Df(3L)Ilp2-3, TI{TI}Ilp2-3 TI{TI}Ilp5$^{3}$/TM3, Sb1 | BDSC | #30889 |
| LexAop- | PBac{LexAop2-myr(Bxb.HA-STOP)4xSNAPf}VK00018; | BDSC | #67632 |
| tub-GPH (tGPH) | w[118]; P{w[+mC]=tGPH}2; Sb[1]/TM3, Ser[1] | BDSC | #8163 |
| tub-Gal80$^{ts}$ | w*; P{w[+mC]=tubP-GAL80[ts]}20; TM2/TM6B, Tb[1] | BDSC | #7019 |

1: Pei-Yu Wang, NTU; 2: Julie Simpson, UCSB; 3: Ann-Shyn Chiang, NTHU; 4: Sheng Li, SCNU; 5: Chia-Lin Wu, CGU; 6: John Tower, USC; 7: Daisuke Yamamoto, NICT; 8:Zhefeng Gong, ZJU; 9: Fly Core Taiwan; 10: Sangbin Park, SU

**Supplementary Table 3. List of primer sequences used for inverse-PCR, transgenic and real-time PCR analysis in this study**

| Targets | Primer name | Sequence (5'→3') |
|---|---|---|
| Inverse PCR | Pry1 | CCTTAGCATGTCCGTGGGGTTTGAAT |
| | Pry2 | CTTGCCGACGGGACCACCTTATGTTATT |
| | Pry4 | CAATCATATCGCTGTCTCACTCA |
| Revertant PCR | 2F | GCGATCGTCTTTTGAGTGGAGAGCAC |
| | 5R | CTGTTCATTGTGCGGGGAGCGTA |
| wake$^{15185}$-Gal4 | OWG2478 | ATCCGTTTCTGCTTGTGACC |
| | OWG2482 | GGCGACTCTCCACTTCAGTC |
| | OWG1824 | CCATCGTGTTTACTGTTTATTGCC |
| | OWG2479 | CTAACGAGCTGCTGCCTTCT |
| wake | wake-F | GTGCCCAGTGTCACATATCG |
| | wake-R | GCAGGATCCAAAGTCAGCTC |
| Br-C | BrC-F | GATGTCAACTTCATGGACCT |
| | BrC-R | ATGGCTGTGTGTGTCCTC |
| E75 | E75-F | ACCACAGCACCACCCATTT |
| | E75-R | TGTTTGGCGGTAGTTTCAGG |
| Kr-h1 | Kr-h1-5F | GATTCCCAGCGTCGGCAGC |
| | Kr-h1-6R | TGGCCGTTCACCAGTGTGGG |
| rpl32 | rpl32-F | AGTATCTGATGCCCAACATCG |
| | rpl32-R | CAATCTCCTTGCGCTTCTTG |

## Supplementary Methods

**Inverse PCR.** *wake*$^{32099}$ is a pUAST-*Mob2-eCFP* transgenic line with *P*-element insertion at 3R: 22666899. For *P*-insertion site mapping using inverse PCR, *wake*$^{32099}$ genomic DNA was digested using *Sau3A*I, and genomic fragments were then self-ligated using T4 DNA ligase, with the *P*-element 3'-flanking sequence rescued by inverse PCR. The first PCR was performed using Pry1 and Pry4 primers and the *Sau3A*I self-ligated fragments as templates. The second PCR was performed using Pry1 and Pry2 primers. All primer sequences used are listed in Supplementary Table 3.

**Transgenic fly generation.** A *wake-Gal4* knock-in fly, *wake-Gal4*$^{15185}$, was created using CRISPR/Cas9-mediated genome editing by WellGenetics, Inc (Taipei, Taiwan). Bases 1 to 7 after ATG of *wake* were deleted (a 7 bp deletion) and replaced with the RMCE-Gal4-w knock-in cassette, containing an attP, a Gal4::VP16, a loxed *white*, and an inverted attP. The sequence with the homology arms and RMCE-Gal4-w is detailed in Supplementary Fig. 5 For *LexAop-wake*$^{mir}$, a 22mer microRNA sequence (CAAATTGCTGCATCAATCAAGC) in exon 4 of the *wake* was artificially synthesized [1] (see Supplementary Data 3) (commissioned in MDBio, Inc.; Taipei, Taiwan) and subcloned into the pP[LexAop-AI] [2] vector using *Kpn*I. For *UAS-wake-RG*$_{HA}$, a full-length *wake* cDNA (GenBank accession no. KJ131166) fused with an HA-tagged coding sequence at its carboxy terminus was synthesized (commissioned in MDBio, Inc., Taiwan) (see Supplementary Data 4) and subcloned into the pJFRC81-10×UAS-IVS-Syn21-GFP-p10 (Addgene plasmid no. 36432) vector using *KpnI*.

**Immunohistochemistry.** Whole mount immunolabeling of adult brains was modified as previously described [3]. The brains were dissected in ice-cold phosphate-buffered saline (PBS) and fixed in 4% paraformaldehyde in 0.01 M PBS on ice for 30 min. Next, the brains were transferred into penetration/blocking buffer (2% Triton X-100, 0.02% $NaN_3$, and 10% normal goat serum in PBS) at room temperature and maintained under vacuum conditions for 1 h. The brains were then mounted in FocusClear (CelExplorer Labs, Taiwan) or incubated overnight with primary antibodies—mouse 4F3 anti-Discs large antibody (anti-DLG; 1:90 dilution; Cat no. AB_528203, Hybridoma Bank, University of Iowa), rabbit anti-Ilp2[4] (1:2000 dilution; gifted by Dr. Veenstra), mouse monoclonal clones, 10F1s [5] (1:5, Developmental Studies Hybridoma Bank at the University of Iowa), DD2.7 [6] (1:10, Developmental Studies Hybridoma Bank at the University of Iowa) and Ag10.2 [7] (1:100, Developmental Studies Hybridoma Bank at the University of Iowa)—and then with biotin-conjugated goat anti-mouse IgG (1:200 dilution; sc-516142, Santa Cruz Biotechnology) or biotin-conjugated goat anti-rabbit IgG (1:200 dilution; sc-2491, Santa Cruz Biotechnology), which were probed using Alexa Fluor streptavidin 635 (1:1000 dilution, S-32364, Invitrogen). Immunostained brains were mounted in FocusClear and imaged using a Zeiss LSM700 Confocal Microscope under a 40 × C-Apochromat water-immersion objective lens.

**SNAP-tagged protein labelling.** To assess driver activity in IPCs, chemical labelling was used to confirm the results of two-colour immunolabelling. A fusion protein myr::SNAP-tag was expressed under the control of appropriate drivers and reacted with its specific fluorescent SNAP substrate to replace traditional immunolabeling. This method is advantageous as it is rapid, has high sensitivity and low background activity, which help to overcome the limitations of traditional immunostaining for thick biological tissues [8, 9]. The method for chemical labelling in

the *Drosophila* brain was modified from a previously described protocol [8]. Briefly, brains were dissected in ice-cold PBS and fixed in 4% paraformaldehyde in 0.1 M PBS at room temperature for 20 min. Thereafter, the brains were transferred to PBS containing 0.3% Triton X-100 (PBST) for 20 min for penetration and then to 1 μM SNAP substrate (SNAP-Surface 488, no. BG-488, New England Biolabs Inc.) in PBST for 1 h. After three 10-min washes with PBST, the brains were mounted in RapiClear CS solution (SunJin Lab Co. Taiwan) and imaged using a Zeiss LSM700 confocal microscope and a 40 × C-Apochromat water-immersion objective lens.

***In situ* Proximity Ligation Assay (*in situ* PLA).** To clarify the interaction between Rdl and WAKE in IPCs, the DuoLink Mouse Rabbit *in situ* PLA kit (Sigma-Aldrich, Cat. no. DUO92101) was utilized in accordance with the manufacturer's protocol. After degassing and blocking described above, brains were incubated with primary antibodies, Rabbit anti-Rdl (1:2000, commissioned in LTK BioLaboratories), mouse anti-HA (1:200, Sigma-Aldrich, Cat. no. H9658), and rat anti-Ilp2 antibody[10] at 1:500 (gift from Dr. Léopold) for 1 day. Next, brains were incubated with secondary antibodies. Following the last wash, the brains were incubated in the anti-mouse and anti-rabbit PLA probes (1:5 dilution in dilution buffer) for 2 hr at 37°C. Brains were then washed three times for 10 min with 1x Wash Buffer A, following which they were incubated in Ligation solution (1:40 ligase in Ligation buffer) for 1 hr at 37°C. Brains were washed three times in 1x Wash buffer A (10 min each time) and then incubated in Amplification solution (1:80 dilution of polymerase in Amplification buffer) for 2 hr at 37°C. Finally, the brains were washed three times in 1x Wash Buffer B (10 min each time), following which they are mounted in RapiClear CS solution (SunJin Lab Co. Taiwan). Negative controls were established via treatment with only anti-Rdl or anti-HA antibodies in the first step. Brains were imaged as described via Zeiss LSM700 confocal microscopy under a 63 × Plan-Apochromat oil-immersion objective lens.

**Antibody production.** A peptide ($H_2N$-CLHVSDVVADDLVLLGEE-OH) was artificially synthesized and applied for rabbit Rdl polyclonal antiserum production (commissioned in LTK BioLaboratories, Inc.; Taiwan). We also performed immunolabelling using the third instar eye discs with exogeneous overexpressed Rdl::HA (*GMR-Gal4>UAS-Rdl::HA*) to assess Rdl antibody specificity (see Supplementary Fig. 10).

***In vivo* calcium imaging.** Flies were immobilized in a 250-μl pipette tip, and a window was opened on the head capsule using fine tweezers, immediately following which a drop of adult haemolymph-like (AHL) saline [108 mM NaCl, 5 mM KCl, 2 mM $CaCl_2$, 8.2 mM $MgCl_2$, 4 mM $NaHCO_3$, 1 mM $NaH_2PO_4$, 5 mM trehalose, 10 mM sucrose and 5 mM HEPES (pH 7.5, 265 mOsm)] was added. After removing the small trachea and excessive fat, the fly and pipette tip were fixed to a coverslip with tape, and a 40X water immersion objective (W Plan-Apochromat 40 X/1.0 DIC M27) was used for *in vivo* calcium imaging. Different concretions (i.e., 100% and 10%) of cVA diluted with ethanol were delivered to the fly head via a custom-made air delivery device. To correct the motion artefacts, frames were aligned using a lightweight SIFT implementation. Response amplitudes were calculated as the mean change in fluorescence ($\Delta F/F_0$) in the 0.1–5 s window after cVA stimulus onset. Time-lapse recordings of changes in GCaMP intensity before and after odor delivery were performed using a Zeiss LSM700 microscope with a 40X water immersion objective, an excitation laser (488 nm), and a detector for emissions passing through a 555-nm short-pass filter. An optical slice with a resolution of

512 X 512 pixels was continuously monitored for 45 s at 2 frames per second. Regions of interest were manually assigned to the DA1 region with GCaMP expression. To evaluate the DA1 responses to cVA, we calculated the changes in GCaMP6 fluorescence as $\Delta F$ $(Ft–F_0)/F_0$. The intensity maps were generated using ImageJ software (National Institutes of Health (Bethesda, MD, USA).

**Pharmacological manipulations.** The working concentrations used in this study were as follows: stock solution of 0.2% precocene I (Cat. no. 195855, Sigma-Aldrich), 0.2% dissolved in ethanol; 3 mM 20-hydroxyecdysone (20E; Cat. no. 16145, Cayman) and 3 mM chromafenozide (Cat. no. 71747, TM standard, China), the latter two of which were also prepared dissolving in ethanol. The same treatment regimen was used for all three drugs—10 µL of drug solution was applied to the food surface and air dried in a laminar flow cabinet for 1 h, and flies were introduced to fresh drug-treated food every 24 h. In addition, IIS inhibitors A-443654 (Cat. no. 16499, Cayman), wortmannin (Cat. no. 10010591, Cayman), and LY294002 (Cat. no. 70920, Cayman) were prepared in DMSO and mixed with fly food to a working concentration of 20 µM, 5µM and 300nM, respectively. Animals in the vehicle control group(s) should be handled identically and treated with the same volume of solvent.

**Quantification of 20E.** For quantification, 20E was extracted from whole bodies of unmated male *D. melanogaster* flies (5–8 days old), as previously described[11]. Flies were collected, frozen, and lyophilized (3,000 specimens). Then, 1 mL of methanol was added to 20 mg of the lyophilized sample, which was then homogenized. The homogenate was stored at –20°C for 5 d, with brief vortexing for 30 s and mixing by tube inversion once a day, then following which it was centrifuged at 13,300 x g and 4°C for 10 min. The supernatant was evaporated to dryness using a nitrogen blow-down evaporator, re-dissolved in 0.1 mL of methanol, and subjected to liquid chromatography (LC)-MS/MS analysis in the multiple reaction monitoring (MRM) mode using an Agilent 1260 Infinity LC system coupled to a ThermoFinnigan LCQ™ advantage mass spectrometer (Thermo Fisher Scientific) equipped with an atmospheric pressure chemical ionization source. High-performance liquid chromatography (HPLC) separation was performed on an Agilent TC-C18 (2) column (150 × 4.6 mm) using water (A) and acetonitrile (B) (0.6 mL/min flow rate at room temperature; gradient conditions: 22% B for 5 min, 22–24% B for 5 min, 24–25.3% B for 5 min, and 25.3–95% B for 7.75 min). The injection volume of the sample was 10 µL. A post-column T splitter was used to split the flow so that the flow rate to the mass spectrometer decreased to 0.18 mL/min. MS/MS analysis was performed under the following conditions: sheath gas, 35 arb; auxiliary gas, 10 arb; discharge voltage, 3,500 V; source temperature, 450°C; source-induced dissociation, 10 V; capillary temperature, 150 °C; and capillary voltage, 14.3 V. MRM analysis was performed using the fragments specific for 20E and makisterone A (as the internal control). The obtained data were processed using Xcalibur 1.3 software (Thermo Fisher Scientific). The concentrations of the compounds in the whole bodies of the flies were estimated using the peak areas of the MRM chromatogram based on a standard calibration curve.

**Lifespan and starvation stress tolerance analysis.** Lifespan and starvation stress tolerance analyses were performed as previously described [12,13]. Five males and five females newly eclosed flies were placed in a food-containing vial. Two days later, 10 flies of the same sex were transferred into fresh food-containing vials. This procedure was repeated every 2 days until no

survivors remained, and the numbers of dead flies in the old vials were counted. For the starvation tolerance test, newly eclosed flies of the same sex were kept in a food-containing vial for 5 to 8 days, after which 10 flies were transferred into vials containing 0.5% agarose only. The number of surviving flies was counted at Zeitgeber time 0 (ZT0; is defined as lights on), ZT6, and ZT11 every day until no survivors remained.

**Locomotor activity assay.** For the negative geotaxis assay of locomotor activity, 10 naïve males were group housed in a 25°C incubator on a 12:12 h light:dark cycle for 5 days. The flies were then transferred into a 250-mL glass graduated cylinder without food, and each cylinder was marked at the 8 cm height to avoid variations in mL marking among the cylinders. After a 10 min adaptation period, the cylinder was lightly tapped 3–5 times, and the climbing ability was recorded. Climbing ability was defined as the percentage of the 20 s recording period that the flies spent above 8 cm target line. At least, six replicates were used for each genotype. For spontaneous mobility assay, flies were transferred into a 5 mm tube containing 2% agarose with 2% sucrose individually. After adaptation to a 12:12 h light:dark cycle, experiments were conducted using a *Drosophila* Activity Monitoring System (DAMS). Spontaneous mobility was assessed for a consecutive 1 min period over 24 h, excluding periods of sleep (0 counts per min lasting >5 min).

**Quantitative RT-PCR.** Fifty adult flies starved for 16 h were homogenized in 1 mL of TriPure Isolation Reagent (Cat. no. 11667165001, Roche Inc.) using a glass-grinder, and total RNA was quantified using a spectrophotometer. cDNA was reverse transcribed using SuperScript™ III Reverse Transcriptase (Cat. no. 11752-050, Invitrogen), and qRT-PCR was performed using the KAPA SYBR FAST qPCR Master Mix (2X) Universal (Cat. no. KK4600, Kapa Biosystems). Data were obtained using Rotor-Gene Q model (QIAGEN) and processed using Rotor-Gene Q Series Software 2.1.0 (QIAGEN). The relative mRNA expression level was normalized to that of Rpl32 and calculated using the $2^{\wedge-\Delta\Delta CT}$method. The primer sequences used are listed in Supplementary Table 3.

**Enzyme-linked immunosorbent assay (ELISA).** ELISA was performed to evaluate circulating Ilp2 levels, as previously described [14]. For the assay, 100 µL of anti-Flag antibody (Cat. no. F1804, Sigma-Aldrich) was diluted to a working concentration of 5 µg/mL with sodium carbonate/bicarbonate buffer (pH 9.4) and incubated overnight in Immuno Clear Standard Modules (Cat. no. 468667, Thermo Scientific). Following incubation, the wells were emptied, washed twice in PBST (PBS with 0.1% Triton X-100), blocked with 350 µL of PBST with 4% non-fat dry milk for 30 min, and then washed three times to remove the residual blocking buffer. Then, 50 µL of anti-HA-peroxidase 3F10 antibody (Cat. no. 12013819001, Roche) was added with block buffer at a dilution of 1:5,000. The sample was then incubated with 3 µL of haemolymph 50 µL of PBST containing 4% BSA overnight. In the last step, wells were washed six times and incubated with 100 µL of TMB ELISA Substrate (Cat. no. 34028, Thermo Scientific) at room temperature for 30 min. The reaction was stopped using 100 µL of 2M sulfuric acid, and absorbance at 450 nm was measured to estimate the circulating concentration of Ilp2.

**Image analysis.** To clarify WAKE affects Rdl levels and trafficking in IPCs, the *UAS-Rdl::GFP* [15] was expressed and analyzed as previously described [1]. The ImageJ software is used for

quantification of Rdl levels on each stack comprising of all images displayed in IPCs and standardized with the intensity of tdTomato to eliminate individual variations. ZEN software (Carl Zeiss MicroImaging GmbH, Germany) is used to calculate the GFP signals around the plasma membrane and perinuclear area for Rdl trafficking analysis. The image stacks are randomly chosen in three or four cells with the maximum nuclear cross-section from an IPCs of each brain, which is then each stack was used to measure total GFP fluorescent intensity across an appropriate region of interest (ROI). For plasma membrane, a region around the entire cell was drawn as $ROI_1$, and then define another area of about 500 nm within $ROI_1$ as $ROI_2$. The intensity of plasma membrane GFP signals of each slide was calculated as $ROI_1$-$ROI_2$. Similarly, the perinuclear area is drawn as $ROI_3$, and the nuclear membrane area is defined as $ROI_4$ within $ROI_3$ about 500 nm. The value, $ROI_3$-$ROI_4$ is regarded as the distribution of Rdl::GFP in the perinuclear membrane. The intensities of the plasma membrane and perinuclear signals converted to the proportion of each stack, ($ROI_1$-$ROI_2$/ $ROI_1$) and ($ROI_3$-$ROI_4$/ $ROI_1$) to verify the effects of Rdl::GFP trafficking in IPCs with WAKE-deficiency. To verify Ilp2 secretion from IPCs, the cells were imaged using a 40× C-Apochromat water-immersion objective at an optical zoom of 2X from the Ilp2-immunolabeled adult brain; identical laser power and scan settings were used for each group compared. A random circular area (diameter, 5 µm) in the plasma of an IPC and ten IPCs from each brain were chosen for measurements of total fluorescence intensity using ZEN software. To compare relative Ilp2 accumulation in IPCs under different treatments or genetic manipulations, the average value of Ilp2 in flies with WAKE downregulation in IPCs was treated as 1. All recorded Ilp2 values were converted to corresponding fold changes for statistical analysis. To investigate InR/PI3K signalling activity, we utilized a previously described method using tGPH flies [16]. The indicator tGPH was used to allow for quantification of PI3K activity by measuring membrane associated GFP fluorescence. Adult fat bodies with tGPH and *wake* mutant flies (*tGHP; wake*$^{32099}$) were used to clarify IIS activity. After dissection and mounting, imaging was obtained using 1.4 NA 40× C-Apochromat water-immersion objective at an optical zoom 1X. The fluorescence intensity of the membrane was measured in eight random circular areas (diameter 5 µm) in each sample. All parts of the plasma membrane were covered in different cells. We demonstrated that brp-GFP labelling can be used to verify the connectivity of Or67d pheromone-sensing neurons. Higher-resolution images of the DA1 olfactory glomerulus were centred on the glomerulus, and Z-boundaries were set according to the confines of the DLG label. Raw image stacks for quantification were previously described [17] and were modified as follows: A region of interest was drawn around the base of individually labelled glomeruli to duplication using Fiji software, and the threshold was manually adjusted within 5% among different samples. Thereafter, background subtraction was selected, and each image was visually examined to ensure that the borders of the spot were sharpened using a watershed. Finally, the numbers of spots were counted and marked using Particles Analysis. To reveal the morphological changes in the DA1 during endocrine disbalance, the GFP intensity and numbers of voxels were calculated. As described above, high-resolution imaging of OSNs was performed using ZEN software. The GFP intensities of DA1 regions in each slide were combined to determine the total GFP intensity, and the GFP pixels in the DA1 region in each slide were considered to represent one DA1 voxel.

## References

1. Liu, S. *et al.* WIDE AWAKE mediates the circadian timing of sleep onset. *Neuron.* **82**, 151–166 (2014).

2. Kuo, S. Y. *et al.* A hormone receptor-based transactivator bridges different binary systems to precisely control spatial-temporal gene expression in *Drosophila*. *PLoS One.* **7**, e50855 (2012).
3. Kuo, S. Y. *et al.* PPL2ab neurons restore sexual responses in aged *Drosophila* males through dopamine. *Nat. Commun.* **6**, 7490 (2015).
4. Veenstra, J. A., Agricola, H. J. & Sellami, A. Regulatory peptides in fruit fly midgut. *Cell Tissue Res.* **334**, 499–516 (2008).
5. Monteiro, A. *et al.* Differential Expression of Ecdysone Receptor Leads to Variation in Phenotypic Plasticity across Serial Homologs. *PLoS Genet.* **11**, e1005529 (2015).
6. Ishimoto, H., Sakai, T. & Kitamoto, T. Ecdysone signaling regulates the formation of long-term courtship memory in adult *Drosophila melanogaster*. *Proc. Natl. Acad. Sci. USA.* **106**, 6381-6386 (2009).
7. Colombani, J. *et al.* Antagonistic actions of ecdysone and insulins determine final size in *Drosophila*. *Science.* **310**, 667–670 (2005).
8. Kohl, J. *et al.* Ultrafast tissue staining with chemical tags. *Proc. Natl. Acad. Sci. USA.* **111**, E3805–E3814 (2014).
9. Sutcliffe, B. *et al.* Second-generation *Drosophila* Chemical Tags: Sensitivity, Versatility, and Speed. *Genetics.* **205**, 1399–1408 (2017).
10. Geminard, C., Rulifson, E. J. & Leopold, P. Remote control of insulin secretion by fat cells in *Drosophila*. *Cell Metab.* **10**, 199–207 (2009).
11. Harshman, L. G., Loeb, A. M. & Johnson, B. A. Ecdysteroid titers in mated and unmated *Drosophila melanogaster* females. *J. Insect Physiol.* **45**, 571–577 (1999).
12. Linford, N. J., Bilgir, C., Ro, J. & Pletcher, S. D. Measurement of lifespan in *Drosophila melanogaster*. *J. Vis. Exp*. **71**, 50068.
13. Zandawala, M. *et al.* Modulation of *Drosophila* post-feeding physiology and behavior by the neuropeptide leucokinin. *PLoS Genet.* **14**, e1007767 (2018).
14. Park, S., Alfa, R. W., Topper, S. M., Kim, G. E., Kockel, L. & Kim, S. K. A genetic strategy to measure circulating *Drosophila* insulin reveals genes regulating insulin production and secretion. *PLoS Genet.* **10**, e1004555 (2014).
15. Fendl, S., Vieira, R. M. & Borst, A. Conditional protein tagging methods reveal highly specific subcellular distribution of ion channels in motion-sensing neurons. *Elife.* **9**, e62953 (2020).
16. Britton, J. S., Lockwood, W. K., Li, L., Cohen, S. M. & Edgar, B. A. *Drosophila*'s insulin/PI3-kinase pathway coordinates cellular metabolism with nutritional conditions. *Dev. Cell.* **2**, 239–249 (2002).
17. Mosca, T. J. & Luo, L. Synaptic organization of the *Drosophila* antennal lobe and its regulation by the Teneurins. *Elife.* **3**, e03726 (2014).
