# Supplementary material for: WAKE-mediated modulation of cVA perception via a hierarchical neuro-endocrine axis in Drosophila male-male courtship behaviour
Source: Nat Commun. 2022 May 6;13:2518. doi: 10.1038/s41467-022-30165-2 (PMC9076693; doi:10.1038/s41467-022-30165-2)
Supplement: Supplementary file 2 — Description to Supplementary Information [file 41467_2022_30165_MOESM2_ESM.pdf]

The legends for Supplementary Data:

Supplementary Data 1. The genotypes and behavior-analytical sample sizes used in each Figure

Supplementary Data 2. The sequence of the knock-in cassette is displayed and coloured as specified to indicate the different segments.

Supplementary Data 3. DNA sequence synthesized for *LexAop-wake*^{mir} generation. *Kpn*I restriction sites are indicated using boxes.

Supplementary Data 4. DNA sequence synthesized for *UAS-wake-RG*$_{HA}$ generation. *Kpn*I restriction sites are indicated using boxes. The HA epitope tag coding region is underlined, and the start and stop codons are indicated in bold.

The legends for Supplementary Video:

Supplementary Movie 1. This movie shows examples of a *wake*^{32099} male courting a wild type 2U male, as referred to in Figure 1b.

Supplementary Movie 2. This movie shows examples of *wake*^{32099} males forming courtship chains, as referred to in Figure 1c.

Supplementary Movie 3. This movie shows examples of *wake*^{32099}/*wake*^{GS17103} trans-heterozygous males forming courtship chains, as referred to in Figure 1c.
