# Supplementary material for: WAKE-mediated modulation of cVA perception via a hierarchical neuro-endocrine axis in Drosophila male-male courtship behaviour
Source: Nat Commun. 2022 May 6;13:2518. doi: 10.1038/s41467-022-30165-2 (PMC9076693; doi:10.1038/s41467-022-30165-2)
Supplement: Supplementary file 4 — Supplementary Data 2 [file 41467_2022_30165_MOESM4_ESM.pdf]

nnnn: vector backbone
NNN: homology arm/intergenic region
NNN: homology arm/coding region
NNN: attP
NNN: Gal4
NNN: mini-white

GTCCGAACAACATGGTCATAGTGCAGGTATTATAGTGTAAAAGCCAAAAGTGAGAAAACCA
GCAGCCAAAGGACTACGCCACGAGCATACAAATGCCAGCCATGCCGTCGCAACTTGTCATA
AGCGGCACCGCCGGCTTACCAGCAAAATAAACAGCGAACACAGGGACACCAGCCAGCAAAC
TCGAGACGCAGCAGCAGCAAACGCAGCAGGATACCAGCCAGGAGCAGCAGCAGCAGCAGCA
GCAGCATCAGCATTCGCACAGCCAACtctagaCCCAGGTCAGAAGCGGTTTTCGGGAGTAG
TGCCCCAACTGGGGTAACCTTTGAGTTCTCTCAGTTGGGGGCGTAGGGTCGCCGACATGAC
ACAAGGGGTTagatctATGAAGCTGCTGAGTAGTATTGAACAAGCGTGTGACATATGCCGA
CTTAAGAAGCTGAAGTGCAGCAAGGAAAAGCCGAAGTGCGCCAAATGTCTTAAGAACAATT
GGGAGTGCCGGTACTCGCCAAAGACGAAGCGAAGCCCTTTGACACGCGCTCATTTGACTGA
AGTGGAGAGTCGCCTGGAGCGCCTTGAGCAGTTGTTCCTCCTCATTTTTCCCCGTGAAGAC
CTCGACATGATACTGAAGATGGATAGTCTCCAAGATATTAAGGCCCTGCTCACTGGCCTGT
TTGTTCAGGATAACGTCAACAAGGATGCCGTGACGGACCGCCTTGCCTCGGTGGAGACAGA
TATGCCACTTACCTTGCGGCAGCATCGCATCTCGGCTACCAGCAGTAGTGAAGAAAGTTCC
AATAAGGGTCAACGGCAACTGACGGTATCGATCGACAGTGCGGCCCACCACGACAACAGCA
CCATTCCACTGGACTTCATGCCTCGTGACGCACTGCATGGATTTGACTGGAGCGAGGAGGA
TGATATGAGCGACGGACTCCCATTCCTGAAGACCGACCCCAATAACAATGGTTTCTTTGGC
GACGGCAGCCTGCTGTGTATCCTGCGAAGTATTGGTTTTAAGCCGGAAAATTACACGAACA
GTAATGTCAATCGTCTTCCCACAATGATCACCGACCGTTACACGCTGGCCAGTCGCAGTAC
TACAAGCCGCCTGCTGCAAAGCTATCTTAACAACTTCCACCCCTACTGCCCCATAGTGCAT
AGTCCTACTCTCATGATGCTTTACAACAATCAGATTGAGATAGCATCGAAGGACCAGTGGC
AGATCCTCTTCAACTGCATTCTCGCCATCGGCGCTTGGTGTATTGAAGGCGAGAGCACAGA
TATCGACGTGTTTTACTATCAAAACGCCAAGTCGCACCTCACCTCGAAGGTCTTCGAAAGT
GGAAGCATTATCCTCGTGACAGCCCTCCATCTGCTGAGTCGTTATACCCAGTGGCGTCAGA
AAACTAATACATCCTACAACTTTCATTCGTTCAGCATTCGGATGGCGATTAGCCTGGGACT
TAATCGTGACCTCCCAAGTAGCTTCAGCGATTCGTCGATCCTGGAACAGCGCCGGAGGATT
TGGTGGAGTGTGTACAGCTGGGAGATCCAGCTGTCCCTCTTGTACGGACGCAGCATTCAGC
TGAGTCAGAACACGATTTCCTTCCCGTCCAGCGTGGACGACGTTCAACGGACAACTACGGG
CCCGACCATCTACCACGGTATCATTGAGACGGCCCGCTTGCTTCAAGTGTTTACGAAGATT
TATGAGCTGGATAAGACAGTGACCGCGGAGAAGAGTCCGATCTGCGCCAAGAAATGCCTGA
TGATCTGCAATGAGATAGAGGAGGTGAGCCGCCAGGCACCCAAGTTCCTGCAGATGGACAT
AAGCACTACGGCGCTGACTAATCTCCTCAAAGAACACCCCTGGCTGAGTTTCACCCGCTTC
GAGCTGAAGTGGAAACAGCTGAGTCTGATTATATACGTTTTGCGGGACTTTTTCACAAATT
TTACGCAGAAGAAGTCGCAGCTTGAGCAAGATCAGAACGACCATCAGTCCTACGAGGTAAA
GAGGTGCAGCATAATGCTCTCCGACGCAGCCCAGCGCACCGTGATGTCGGTGTCGTCCTAT
ATGGACAACCATAATGTGACCCCTTATTTCGCCTGGAATTGCTCCTACTATTTGTTCAACG
CCGTGTTGGTCCCAATCAAGACTCTTCTGAGCAACTCCAAATCGAACGCCGAGAATAACGA
GACAGCACAGCTCTTGCAGCAAATTAACACCGTGTTGATGCTGTTGAAGAAACTCGCTACA
TTCAAGATTCAGACTTGCGAAAAGTACATCCAGGTGTTGGAAGAGGTGTGCGCCCCATTCC
TTCTTTCGCAATGCGCAATTCCCCTCCCGCACATAAGTTACAATAATTCCAACGGCTCCGC
TATCAAGAATATCGTCGGCTCCGCTACAATCGCGCAGTATCCCACGCTGCCTGAGGAAAAT
GTTAACAACATATCGGTGAAGTATGTAAGTCCAGGCAGCGTCGGCCCGAGCCCTGTGCCGC

```
TGAAAAGCGGAGCCTCCTTCAGCGATCTGGTCAAGCTGCTCAGCAACCGCCCACCATCCCG
CAACTCCCCTGTCACTATACCCCGATCGACCCCGTCGCATCGTAGCGTTACCCCATTCCTG
GGACAACAGCAGCAACTGCAGAGTCTGGTGCCACTTACGCCCTCGGCATTGTTTGGCGGAG
CCAACTTCAACCAGAGTGGTAACATTGCGGACAGCTCCTTGAGCTTCACGTTTACCAACAG
CTCCAACGGTCCAAACTTGATCACGACTCAGACAAACTCCCAGGCATTGTCCCAGCCCATT
GCCAGCTCGAATGTGCATGATAACTTTATGAATAATGAAATTACTGCCTCCAAGATTGACG
ACGGCAACAACAGCAAGCCCCTTTCCCCAGGCTGGACTGACCAGACGGCCTACAATGCTTT
CGGTATCACCACTGGCATGTTCAACACCACAACGATGGACGACGTATATAACTATCTTTTT
GATGATGAGGATACTCCACCGAACCCAAAGAAGGAGTAGgatctataacttcgtataatgt
atgctatacgaagttatggtaccttgcatgccccgttattcTCTATTCTCTATTCGTTTTG
TGACTCTCCCTCTCTGTACTATTGCTCTCTCACTCTGTCGCACAGTAAACGGCACGCTATT
CTCGTTGCTTCGAGAGAGCGCGCCTCGAATGTTCGCGAAAAGAGCGCCGGAGTATAAATAG
AGGCGCTTCGTCGACGGAGCGTCAATTCAATTCAAACAAGCAAAGTGAACACATCGCGAAG
CGTAAGCTGAGCAAACAAACAAGCGCAGCTGAACAAGCTAAACAATCTGCAATAAAGTGCA
AGTTAAAGTGAATCAATTAAAAGTAACCAACAACCAAGTAATTAAACTAAAAACTGCAACT
ACTGAAATCAACCAAGAAGTCATTATTGAAGACAAGAAGAGAACTCTgaataggtcgatag
cgtcaattcggcacgagcggtttcgtgacgaagctccaagcggtttacgccatcaattaaa
cacaaagtgctgtgccaaaactcctctcgcttcttattttttGTTTGTTTTTTGAGTGATTG
GGTGGGTGATTGGTTTTGGGTGGGTAAGCAGGGGAAAGTGTGAAAAATCCCGGCAATGGGC
CAAGAGGATCAGGAGCTATTAATTCGCGGAGGCAGCAAACACCCATCTGCCGAGCATCTGA
ACAATGGTGACAGCGGAGCGGCTTCGCAGAGCTGCATTAACCAGGGCTTCGGGCAGGCCAA
AAACTACGGCACGCTCCTGCCACCCAGTCCGCCGGAGGACTCCGGTTCAGGGAGCGGCCAA
CTAGCCGAGAACCTCACCTATGCCTGGCACAATATGGACATCTTTGGGGCGGTCAATCAGC
CGGGCTCCGGATGGCGGCAGCTGGTCAACCGGACACGCGGACTATTCTGCAACGAGCGACA
CATACCGGCGCCCAGGAAACATTTGCTCAAGAACGTTTGCGGCGTGGCCTATCCGGGCGAA
CTTTTGGCCGTGATGGGCAGTTCCGGTGCCGGAAAGACGACCCTGCTGAATGCCCTTGCCT
TTCGATCGCCGCAGGGCATCCAAGTATCGCCATCCGGGATGCGACTGCTCAATGGCCAACC
TGTGGACGCCAAGGAGATGCAGGCCAGGTGCGCCTATGTCCAGCAGGATGACCTCTTTATC
GGCTCCCTAACGGCCAGGGAACACCTGATTTTCCAGGCCATGGTGCGGATGCCACGACATC
TGACCTATCGGCAGCGAGTGGCCCGCGTGGATCAGGTGATCCAGGAGCTTTCGCTCAGCAA
ATGTCAGCACACGATCATCGGTGTGCCCGGCAGGGTGAAAGGTCTGTCCGGCGGAGAAAGG
AAGCGTCTGGCATTCGCCTCCGAGGCACTAACCGATCCGCCGCTTCTGATCTGCGATGAGC
CCACCTCCGGACTGGACTCATTTACCGCCCACAGCGTCGTCCAGGTGCTGAAGAAGCTGTC
GCAGAAGGGCAAGACCGTCATCCTGACCATTCATCAGCCGTCTTCCGAGCTGTTTGAGCTC
TTTGACAAGATCCTTCTGATGGCCGAGGGCAGGGTAGCTTTCTTGGGCACTCCCAGCGAAG
CCGTCGACTTCTTTTCCTACGTGGGTGCCCAGTGTCCTACCAACTACAATCCGGCGGACTT
TTACGTACAGGTGTTGGCCGTTGTGCCCGGACGGGAGATCGAGTCCCGTGATCGGATCGCC
AAGATATGCGACAATTTTGCCATTAGCAAAGTAGCCCGGGATATGGAGCAGTTGTTGGCCA
CCAAAAATTTGGAGAAGCCACTGGAGCAGCCGGAGAATGGGTACACCTACAAGGCCACCTG
GTTCATGCAGTTCCGGGCGGTCCTGTGGCGATCCTGGCTGTCGGTGCTCAAGGAACCACTC
CTCGTAAAAGTGCGACTTATTCAGACAACGATGGTTGCCATCTTGATTGGCCTCATCTTTT
TGGGCCAACAACTCACGCAAGTGGGCGTGATGAATATCAACGGAGCCATCTTCCTCTTCCT
GACCAACATGACCTTTCAAAACGTCTTTGCCACGATAAATGTGTTCACCTCAGAGCTGCCA
GTTTTTATGAGGGAGGCCCGAAGTCGACTTTATCGCTGTGACACATACTTTCTGGGCAAAA
CGATTGCCGAATTGCCGCTTTTTCTCACAGTGCCACTGGTCTTCACGGCGATTGCCTATCC
GATGATCGGACTGCGGGCCGGAGTGCTGCACTTCTTCAACTGCCTGGCGCTGGTCACTCTG
GTGGCCAATGTGTCAACGTCCTTCGGATATCTAATATCCTGCGCCAGCTCCTCGACCTCGA
```

TGGCGCTGTCTGTGGGTCCGCCGGTTATCATACCATTCCTGCTCTTTGGCGGCTTCTTCTT
GAACTCGGGCTCGGTGCCAGTATACCTCAAATGGTTGTCGTACCTCTCATGGTTCCGTTAC
GCCAACGAGGGTCTGCTGATTAACCAATGGGCGGACGTGGAGCCGGGCGAAATTAGCTGCA
CATCGTCGAACACCACGTGCCCCAGTTCGGGCAAGGTCATCCTGGAGACGCTTAACTTCTC
CGCCGCCGATCTGCCGCTGGACTACGTGGGTCTGGCCATTCTCATCGTGAGCTTCCGGGTG
CTCGCATATCTGGCTCTAAGACTTCGGGCCCGACGCAAGGAGTAGCCGACATATATCCGAA
ATAACTGTTTGTTTTTTTTTTTTACCATTATTACCATCGTGTTTACTGTTTATTGCCCCCT
CAAAAAGCTAATGTAATTATATTTGTGCCAATAAAAACAAGATATGACCTATAGAATACAA
AAAAAAAAAAAAAAAAAAAAAAAActcgaaggggcccggctgcagataacttcgtataat
gtatgctatacgaagttatgctagcAACCCCTTGTGTCATGTCGGCGACCCTACGCCCCCA
ACTGAGAGAACTCAAAGGTTACCCCAGTTGGGGCACTACTCCCGAAAACCGCTTCTGACCT
GGGctcgagggcgcgAGGTCCTGCAAAGAAAAGCGGCACCACCCTATCCGTCGACCGGGCC
GCCTTCCTTTTGCTGCGCGTCAGGAAAGTGTTCAAGAAGAAGCGACAGCAGCGCAAGGAGC
GACA

Supplementary Data 2. The sequence of the knock-in cassette is displayed and coloured as specified to indicate the different segments.
