# Supplementary material for: WAKE-mediated modulation of cVA perception via a hierarchical neuro-endocrine axis in Drosophila male-male courtship behaviour
Source: Nat Commun. 2022 May 6;13:2518. doi: 10.1038/s41467-022-30165-2 (PMC9076693; doi:10.1038/s41467-022-30165-2)
Supplement: Supplementary file 5 — Supplementary Data 3 [file 41467_2022_30165_MOESM5_ESM.pdf]

GGTACCCGCCAGATCTTTTAAAGTCCACAACTCATCAAGGAAAATGAAAGTCAAAGTTGGCAGC
TTACTTAAACTTAATCACAGCCTTTAATGTCAAATTGCTGCATCAATCACGCTAAGTTAATATA
CCATATCTAGCTTGATTGATGCAGCAATTTGGTAgCTAAAGTGCCTAACATCATTATTTAATTT
TTTTTTTTTTTGGCACACGAATAACCATGCCGTTTTGGATCTTTTAAAGTCCACAACTCATCAA
GGAAAATGAAAGTCAAAGTTGGCAGCTTACTTAAACTTAATCACAGCCTTTAATGTAAGCTGAC
CAGAATCATAACCGTAAGTTAATATACCATATCTACGTTTATGATTCTGGTCAGCTTGTACCTA
AAGTGCCTAACATCATTATTTAATTTTTTTTTTTTTTGGCACACGAATAACCATGCCGTTTTGG
ATCCGTGGTACC

Supplementary Data 3. DNA sequence synthesized for *LexAop-wake*^{mir} generation. *Kpn*I restriction sites are indicated using boxes.
