# Supplementary material for: WAKE-mediated modulation of cVA perception via a hierarchical neuro-endocrine axis in Drosophila male-male courtship behaviour
Source: Nat Commun. 2022 May 6;13:2518. doi: 10.1038/s41467-022-30165-2 (PMC9076693; doi:10.1038/s41467-022-30165-2)
Supplement: Supplementary file 6 — Supplementary Data 4 [file 41467_2022_30165_MOESM6_ESM.pdf]

GGTACC**ATG**GAGCAGGATGCCGCAGCAGGTCCTGCAAAGAAAAGCGGCACCACCCTATCCGTCG
ACCGGGCCGCCTTCCTTTTGCTGCGCGTCAGGAAAGTGTTCAAGAAGAAGCGACAGCAGCGCAA
GGAGCGACAAGCCCAGCAAAGTGCCGCCGCAGCGGCGAGTGGTGCCGGCGGTGCGGGTGGTGCC
GGTGGTGGTGGCGGTGGCTCCGGGTCACAATCGCGACCAGGCAGCCGGAAGCTGCCGCCGCCAT
TGTTACGGTCGCGCACACTGCCCGCCATCATTGTGCCAGGACTTCCGGTCGTCTCGCTGCACAC
GGATAAGCAAACATTTCAGCTGGACGAACGTTTGTTGGGCAGCAAAAGCCGCAGTGGCAGCGCC
AGTGGCCATCGATGGTCGCTGCTCACACGTGCCACCAACAGCAGCAGCATGTGCAGCAACAACA
ACCCGGCCAGCAGTAGCAGCAGCAGCAGCAACAACAATAACAATTTACACATGGCCAACAACAA
TAACACACTGATGGTGAAATCATTGAATTTGCCCAAAGACGATAGCAATTTGGTCTATCGCCGC
AAATCCTCGGGCAGCACGCCCCACCAACATCAGAATGCGCACCATCATCATCAACAGCATCATC
ACCTGCAGCAGCAGCAGCAACAGCAGCAGCAGCATCAGCAGCAGCAACTGCAACAGCAGCAGAT
GCTGCAGCATCTGGGGGACAGCTCGCTGTTTCAGCTGTCCGACGATTTCGAGTGCCACGCGGAT
GGTTCCCTGCTCCTCAGAATTCCAGCTCCTCATGTGGTCGCCGCACGAGCCTATCGACTGGCAG
CTCGAAAGCGAGCTGCCGCCTCCTCGGAGGCCTCCACACCGATGACCCGTGAGCACACGCTCCA
CGAGATGCCGCTGGCGGAGTCGGCCTGCTCCGGATCTGCCACTGGTTCGGGCACGACCTTCACG
CGTCTGGCCAAATTGCTGCATCAATCAAGCACGCTGGCGGGCAGGTCCAATCTGCAGCCACCGC
CCATGGCTGACAATGCGGTCCAGAGCCTGGGCAGAGGATTGGGCCTGGGACTGGGCGAGGAGGC
GCCACGTCGTCTGTCCTGGGAGAGGCGCAAGGACAGCAGTGCTCTGCAGCGTTCGGCCAGCATT
GATTCCTTTGCCGAGATTGTGTTTAGTGAGTCGCCACGCCCCTCGCTGGCTGTCACGGGACCAG
GTGCCTGCGCCTCCGCATCCGGCGGCCCTTCGCCATTCAGCAAACGTCCTTCGGCCAGCAGCCT
GTACTCCACCTCAACGTCCAGCTCCTTCGCATCGCAGATGCAGGCCCAGCTGAATGTCAATTAC
GGCGACTCGGCACTGGGATCCGGATCAGCAACGGGTGGACCGGGCTCGGGTCACCACCATGGGG
CATCTGGTGGCGGCAACGGGGGCAGCAGTGCCAGCAGCAGTCGGCGCGAAAGCATGCTCAGCCC
CTCATCCACGCGCCGCTCCAAGCTGACCAGAATCATAAACGCTCTCTTCTCGGCAGTGGAGCAT
GGTCACCTGGAGAAGGCGCGCACTATTCTAGAGTCCACCGACGTAGATGTAAACAGCATCAATA
ACGATGGACTATCTGCTCTGGACTTGGCGGTGTTAAGCAATAATCGCTCCATGACCAGGATGCT
GCTTCAGCATGGCGCTGTGGAGGGCTCTCAGTTCTCTGTGGACACCATTGGCACCAAACTGAAC
GGCTTGCTCAAGGACGCCGAGTCAAGAATTCACGATCTAAGTGGACCGGAGGGCCTCTGTCCGC
CGGTGTTTGCATCGCGTCCCTCCATCTCGAGCATCATAATTGGCAATTCCAGTGCATCGGTAAC
TGGCTGCACGGGCAGCGAGGTGGAAAAACAGATAGGCATCTGGGAGCGCCGAGTTAAGGGACTG
CGTCGCTTGCAGCTCGGTTGGGATCAGGCCAGGCCGCCAGATGCACCCGCCTCCGTGGTCGTCG
ACGTCACTGGCGACAATTCCATCAGCGTACAAATCCTAGAGCCCTTCGAGGGGGCCATCGGCAC
GAAATTTAAAGTACAATGGTCGACCCGCGCGGATTTCAACAACGTTGTGGGCGAGAGTGAGCTA
CTGGAATGGATCAGCTTCCATGGCACGATGGGTGCCCAGTGTCACATATCGGGCCTCACCCAGG
GTCGTCGCTACTTCCTGCGAGCCGCGTGCGGCAATGTGAAGGGATGGGGCACCTATCGAACCTC
TGTGCCGGCTAGTGTAGTGCCCTCAACTTGGCGGGATTTAGACAATCGAGAGGACCGATTCGTG
GGTCGTCACCGAATCCTGGACAATCTATTTACCGCCGTGCGTTTGGCAAGACCCGCCGACGTAT
CCGAGCTGACTTTGGATCCTGCGAGCGCCCAACGACGTAATCCCAAAAAGAAGACTACCATTAA
GCAGTTGTTCTCGGTAACCACCAAGTTTCAAAAGACACTGAGACGCGGCATTTACTTCTCTTGT
ATTATTCATTGCGATGATAAAGTGCTGGTCACCAGCGAAGACTTTCCACCGGTCATCGAGGTGG
ATGAATCCTATCCGAGTGCTTTGCACATGGATTACTACTGGCTCATGAAAGTTGCCTGCACCTG
GGAGGATGTTAAGTCTCTACGTTCGGATATGGAGCGCAATCTTACCTCCGCTGTTCACTTCCGC
ACTAAGCTTCTATCGGCGGTCTGTCAGATGCAATCGGCACTAGGCATTACGGACTTGGGCCAAC
TTTACTATAAACCATTGCGGGATGCTCAGGGCACTGTGGTCCTAACCTGTGTGCAGTCGGTAAA
GAGCCAAAAGGCCGTCTCCATCTTAAACTCGAGATGGGTTCCAGTCAGCAAGCTGCAAAAGAAG
CTAGGTGCTCTACACGAGGACTACACCATTAACGAGCTGCTCATCTCCTCGATTGGAGATCAAT
TGCACTATCAACAGGCGGCGCTGCAGCGTTTGGAGCCCGGTCTTTACCTGGGCTACCTAAAGAT

GCAATGCTCCATAGACCAGATCCAGGTGGTTGTGCCCGTGAAAACGCCGAATGTCTTGCCCCAC
TGCAAGGTGCGCGAAAACAGTCACATCACGGCCGAAGAGTGGCAGGTGCTCCACCGTTACAGTA
GCGATCCTCTACGCCTGCCGTTGGACTTTAGCGCTCAAGGAGGAGATGGAGCCTCCGGAGCAAC
AGCTACTACGGAGGTGCAACGCCTATTTTTATACGATCTCACCAATGCAATGCACAAACTGTTT
GCCAGTATGAACATCAAAATCGCTGATGCAACCACTCACCGCTTGTACGACGTGGAGGTGATTG
AGCATAGTCCGGATATTAGTTTCCTCGTGGTGTGTCCATCCGCCGAGTCCTCATGCGCTGTGCC
CGGTCAGTCTGAGTTACTGCTACAGCGAGATGACTTGGCCAGCTTGAGTATCCAGGCCTTTGAG
ATGATTCACCTGCGCACTTATCAGCCGGCCATCATTCAGAAGTACGCCCGTTTATCTTGCATCC
TTGAATTGGATACCGCACTGGCCACACATTCGCTTCGCGAGGCATTCTCCAGCAGTGAACTGCA
GGCGGCCAAGGAACGTTTGGCCACGCTGCAGGAGCTCAGTGCGAGTCTTACGATTGTGTGGAAA
AGTGTGCGATGGCTAATGGATGTGGTGGCATATGCGCGAAACAAAAACGCTCAACCCTCGTTGG
CTATGCGAGAAATTCTGGACTTTGCACAGCAAAGACAGGACGAAGCGGTAGCGACATCGGCAGG
AGGTTCCGCGAACAAACAGCTGCTGCAGCTGCCCATCCGCGAGAGCAAGTTCTCCAAGACGGGC
CAGGGAAGAGGCAGTTGGCCGGGACCGGGAACTAGTGAAGATCAATCGCAGAACAAGCCGGAGC
ACTCCAAGTCTGAACAGAATCTGGAACTGAGTGCTATCACGCCAGTGGAACCTGCATCCAATCA
AGTTCCTACACAGCAGCAGCAGCCGCAGCAGCAATCGCAGCAACAGCAACTCTTACAAGTTTCC
AACATCAGCGAATACGCGGGATCGATTTGCTCAGAAGTATCATTTAGGAAGAATAGTGGCGATT
CCATGAGCTCCACCTACACGTCCCGGAGCTTCTACTCTGCCGTGGACTCAGCCTCAGATGGAAA
TAGCACAAACAGTGTGTTTGCCATTCCGCCCTCTCGTTCCGACGACACTCTGGCGGATGCACTG
CGCCACTCGCAAGCGGTAGCTGCGCAACGCAAACGAACCAGCTCTAATATCGCATCTCATACCA
ATCCCTTGATTACGGTGCACAGCTCTAGTTCGGCTCCGTACCTGGCTGGATCCAGTGTTTCCTT
GAGAAGCGGCAACGGAGCCTTCGCAACGGATTTGGAGCACAAACCGCTGAAGCCAGCGACAAAG
GCTGCATCTAGTGCAAACCTGCGCGAAGGTGGACCCTATTTGAAAAGCACGTTAGCTGAACTAC
GAGCTGTTGGAGGTGAGGAGCCGGTGGCCAGTACTTCAAAGGCCTCTTCGATGAAGAGCTTGTC
GCGGCAGAGCAGCGAAGAGGATTCCGCCAGCTGTTCCAGTCTCAACGCAGAGCAGACATCGGGG
ATCATTCAAGTCTATACCGCCTACAGTACGGGTCTGGCCAGTGGAACCAGCTTAAAGATTCACG
TAACTCCGAAGACGACTGCCCGCGAAGTGATCAACCTGGTGGTGAAGCAACTTAACATGGCCGT
GGTTCTCAAAGGGAACAATGGCCCCATCTATGGACCCGAGATGCTAGAGAACTTTTGCCTGGTT
GCGGTGATTGGAGCAAGGGAACGCTGCCTGCGAGATGACTTCAAACCGCTGCAACTGCAGAATC
CCTGGAAGAAGGGCCGTCTGTACGTCCGCAAGAAGCATGAGCTGCTGGCGGCCATCGAACACTC
GAATCGGAAATCGCATTTGTACCCTTACGACGTGCCTGACTACGCAATC**TGA**GGTACC

Supplementary Data 3. DNA sequence synthesized for *UAS-wake-RG*$_{HA}$ generation. *Kpn*I restriction sites are indicated using boxes. The HA epitope tag coding region is underlined, and the start and stop codons are indicated in bold.
