# Supplementary material for: WAKE-mediated modulation of cVA perception via a hierarchical neuro-endocrine axis in Drosophila male-male courtship behaviour
Source: Nat Commun. 2022 May 6;13:2518. doi: 10.1038/s41467-022-30165-2 (PMC9076693; doi:10.1038/s41467-022-30165-2)
Supplement: Supplementary file 10 — Reporting Summary [file 41467_2022_30165_MOESM10_ESM.pdf]

nature research

Corresponding author(s): Yu-Chen Tsai, Huey-Fen Tzeng, Tsai-Feng Fu

Last updated by author(s): Apr 7, 2022

# Reporting Summary

Nature Research wishes to improve the reproducibility of the work that we publish. This form provides structure for consistency and transparency in reporting. For further information on Nature Research policies, see our Editorial Policies and the Editorial Policy Checklist.

## Statistics

For all statistical analyses, confirm that the following items are present in the figure legend, table legend, main text, or Methods section.

| n/a | Confirmed | |
|---|---|---|
| ☐ | ☒ | The exact sample size (*n*) for each experimental group/condition, given as a discrete number and unit of measurement |
| ☐ | ☒ | A statement on whether measurements were taken from distinct samples or whether the same sample was measured repeatedly |
| ☐ | ☒ | The statistical test(s) used AND whether they are one- or two-sided<br>*Only common tests should be described solely by name; describe more complex techniques in the Methods section.* |
| ☒ | ☐ | A description of all covariates tested |
| ☐ | ☒ | A description of any assumptions or corrections, such as tests of normality and adjustment for multiple comparisons |
| ☐ | ☒ | A full description of the statistical parameters including central tendency (e.g. means) or other basic estimates (e.g. regression coefficient) AND variation (e.g. standard deviation) or associated estimates of uncertainty (e.g. confidence intervals) |
| ☐ | ☒ | For null hypothesis testing, the test statistic (e.g. *F*, *t*, *r*) with confidence intervals, effect sizes, degrees of freedom and *P* value noted<br>*Give P values as exact values whenever suitable.* |
| ☒ | ☐ | For Bayesian analysis, information on the choice of priors and Markov chain Monte Carlo settings |
| ☐ | ☒ | For hierarchical and complex designs, identification of the appropriate level for tests and full reporting of outcomes |
| ☒ | ☐ | Estimates of effect sizes (e.g. Cohen's *d*, Pearson's *r*), indicating how they were calculated |

*Our web collection on statistics for biologists contains articles on many of the points above.*

## Software and code

Policy information about availability of computer code

| | |
|---|---|
| Data collection | Zeiss Zen software (2010B SP1) were used to acquire images.<br>Behavioral test were collected by manual.<br>qPCR raw data were collected using Rotor-Gene Q Series Software 2.1.0.<br>ELISA data were obtained using Microplate Manager 5.2.1.<br>liquid chromatography (LC)-MS/MS analysis in the multiple reaction monitoring (MRM) mode using an Agilent 1260 Infinity LC system coupled to a ThermoFinnigan LCQTM advantage mass spectrometer (Thermo Fisher Scientific) equipped with an atmospheric pressure chemical ionization source |
| Data analysis | ZEN software (Carl Zeiss MicroImaging GmbH, Germany) was used to verify the Ilp2 secretion from IPCs (Fig. 6) and quantity GFP intensity and Volex (Supplementary Fig. 14) as described in the Supplementary Methods section.<br>ImageJ2/FIJI (Version 2.1.0), Analyze Particles, were used to count Puncta number (Fig. 9) as described in the Supplementary Methods section.<br>(LC)-MS/MS obtained data were processed using Xcalibur 2.0 software (Thermo Fisher Scientific)<br>All data were analyzed using GraphPad Prism 9. |

For manuscripts utilizing custom algorithms or software that are central to the research but not yet described in published literature, software must be made available to editors and reviewers. We strongly encourage code deposition in a community repository (e.g. GitHub). See the Nature Research guidelines for submitting code & software for further information.

## Data

Policy information about availability of data

All manuscripts must include a data availability statement. This statement should provide the following information, where applicable:

- Accession codes, unique identifiers, or web links for publicly available datasets
- A list of figures that have associated raw data
- A description of any restrictions on data availability

All data generated or analysed during this study are included in this published article (and its supplementary information files). The raw data are provided as a Source Data file and available from the corresponding author upon request.

# Field-specific reporting

Please select the one below that is the best fit for your research. If you are not sure, read the appropriate sections before making your selection.

☒ Life sciences ☐ Behavioural & social sciences ☐ Ecological, evolutionary & environmental sciences

For a reference copy of the document with all sections, see nature.com/documents/nr-reporting-summary-flat.pdf

# Life sciences study design

All studies must disclose on these points even when the disclosure is negative.

| | |
|---|---|
| Sample size | Sample size were equal to or more than 6 for chaining test and 15 for pair test, and 12 for preference test, each qPCR test were least 3 replicates and 4 for ELISA test.<br>Exact Sample size were indicated in Source Data.<br>For image quantitative experiments involving quantification of ilp2 secretion (Fig.6), puncta, GCamP (Fig. 9), and GFP intensity and Voxel (Supplementary Fig. 14) n=8 was chosen as the minimal replicate number; and image qualitative experiments including wake-Gal4 drivers (Fig. 3b-n), ilp2-Geneswtich (Fig.4a-b) and Rdl-Gal4 (Fig. 5a, b), sample sizes more than 6 for image acquire.<br>Exact sample size were indicated in Supplementary Table 3.<br>All sample size was determined base on literatures in the field. |
| Data exclusions | No data were excluded from analysis. |
| Replication | All replication were repeated on different days with different batch of flies. |
| Randomization | For behavioural experiments, courter flies were collected into separate 2.0 cc centrifuge, and courtee flies were collected collected into common vials, all samples were randomly selected for experiments.<br>For the rest experiments, flies were collected into common vials.<br>All experimental and control flies were collected, treated, and experiment were conducted in the same period of time. |
| Blinding | Researchers were blinded during data collection and analysis. |

# Reporting for specific materials, systems and methods

We require information from authors about some types of materials, experimental systems and methods used in many studies. Here, indicate whether each material, system or method listed is relevant to your study. If you are not sure if a list item applies to your research, read the appropriate section before selecting a response.

### Materials & experimental systems

| n/a | Involved in the study | |
|---|---|---|
| ☐ | ☒ | Antibodies |
| ☒ | ☐ | Eukaryotic cell lines |
| ☒ | ☐ | Palaeontology and archaeology |
| ☐ | ☒ | Animals and other organisms |
| ☒ | ☐ | Human research participants |
| ☒ | ☐ | Clinical data |
| ☒ | ☐ | Dual use research of concern |

### Methods

| n/a | Involved in the study | |
|---|---|---|
| ☒ | ☐ | ChIP-seq |
| ☒ | ☐ | Flow cytometry |
| ☒ | ☐ | MRI-based neuroimaging |

## Antibodies

| | |
|---|---|
| Antibodies used | Antibodies used for imaging<br>1. Anti-Discs large (Hybridoma Bank, University of Iowa, Cat. AB_528203), used at 1:90<br>2. Anti-dilp2 (gifted by Dr. Veenstra which was shown as supplementary data reference 4), used at 1:2000 |


April 2020

3. Goat anti-mouse-IgG-Biotin (Santa Cruz Biotechnology, Inc., Cat. sc-2039), used at 1:200
4. Mouse anti-Rabbit-IgG-Biotin (Santa Cruz Biotechnology, Inc., Cat. sc-2491), used at 1:200
5. Alexa Fluor streptavidin 633 (Invitrogen, Cat. #S32364), used at 1:1000
6. Mouse anti-EcR (Developmental Studies Hybridoma Bank at the University of Iowa, Cat. 10F1s), used at 1:5
7. Mouse anti-EcR (Developmental Studies Hybridoma Bank at the University of Iowa, Cat. DD2.7), used at 1:10
8. Mouse anti-EcR (Developmental Studies Hybridoma Bank at the University of Iowa, Cat. Ag10.2), used at 1:100

Antibodies used for PLA
1. Mouse anti-HA (Sigma , Cat. #9658), used at 1:200
2. Rabbit anti-Rdl (commissioned in LTK BioLaboratories), used in 1:2000
3. Anti-dilp2 (gifted by Dr. Veenstra which was shown as supplementary data reference 4), used at 1:500

Antibodies used for ELISA
1. Anti-Flag (Sigma-Aldrich; F1804), used at 1:200
2. Anti-HA-peroxidase 3F10 antibody (Roche, 12013819001), used at 1:5000

Validation

1. Anti-Discs large validated in previous literature (Kuo, S. Y. et al., 2015; DOI: 10.1038/ncomms8490)
2. Anti-dilp2 for image experiments validated in publish literature (Veenstra, J. A. et al., 2008; DOI: 10.1007/s00441-008-0708-3)
3. Anti-Flig, mouse monoclonal ANTI-FLAG ® M2 antibody and Anti-HA peroxidase 3F10 antibody validated in publish literature ( Park, S. et al., 2014; DOI: 10.1371/journal.pgen.1004555)
4. Mouse anti-EcR (Cat. 10F1s) validated in publish literature (Monteiro, A. et al., 2015; https://doi.org/10.1371/journal.pgen.1005529)
6. Mouse anti-EcR (Cat. DD2.7) validated in publish literature (Ishimoto, H. et al., 2009; DOI: 10.1073/pnas.0810213106)
7. Mouse anti-EcR (Cat. Ag10.2) validated in publish literature (Colombani, J. et al., 2005; DOI: 10.1126/science.1119432)
8. Mouse anti-HA (H9658) validated in publish literature (Cohen, S. M. et al., 2018;https://doi.org/10.1038/s41467-018-04705-8)
9. Rabbit anti-Rdl validated in this study (Fig. S10)
10. anti-dilp2 antibody for PLA assay validated in publish literature (Géminard, C. et al., 2009; DOI: 10.1016/j.cmet.2009.08.002)

## Animals and other organisms

Policy information about studies involving animals; ARRIVE guidelines recommended for reporting animal research

Laboratory animals

Drosophila melanogaster wild type and all transgenic strains used is provided in Supplementary Table 2.
Most strains used in this study were from Bloomington Drosophila Stock Center and Vienna Drosophila Resource Center. ilp2-Gal4, ilp2-GeneSwitch, UAS-Ilp2, UAS-InRDN, UAS-InRCA were from Dr. Pei-Yu Wang at NTU; rdl2-1-Gal4 were from Dr. Julie Simpson at UCSB; Or67d-Gal4, pdf-Gal4, 2U were from Dr. Ann-Shyn Chiang at NTHU; Jhamt-Gal4 were from Dr. Li Sheng at SCNU; elav-Gal4, UAS-GCamp6 were from Dr. Chia-Lin Wu at CGU; actin-GeneSwitch were from Dr. John Tower at USC; UAS-Jhamt were from Dr. Daisuke Yamamoto at NICT; ilp2-LexA were from Dr. Zhefeng Gong at ZJU; w1118, Canton-S, Oregon-R were from FlyCore Taiwan; Ilp2HF were from Dr. Sangbin Park at SU.

Wild animals

This study did not involve wild animals.

Field-collected samples

This study did not involve samples collect from the field.

Ethics oversight

As a general practice, no ethics guidance was required for the insect included in this study.

Note that full information on the approval of the study protocol must also be provided in the manuscript.
